# Supplementary material for: Assessing Genotype-Phenotype Correlations with Deep Learning in Colorectal Cancer: A Multi-Centric Study
Source: medRxiv. 2025 Feb 8:2025.02.04.25321660. Preprint. [Version 1] doi: 10.1101/2025.02.04.25321660 (PMC11838662; doi:10.1101/2025.02.04.25321660)
Supplement: Supplement 1 [file media-1.pdf]

Supplementary Methods

Data Analysis of Genetic Alterations

We applied hierarchical clustering[1,2] to group genes and conditions, such as MSI and hypermutation, based on their mutational patterns. This method organizes data into a hierarchy of clusters, where similarities are quantified using the commonly applied 'Euclidean' metric, which measures distances in a multi-dimensional space. To optimize cluster formation, we used the 'Ward' method[3], which minimizes within-cluster variance, resulting in more balanced and interpretable clusters. To mitigate the influence of missing values, we restricted clustering to prediction targets with complete data (Table S1), ensuring robust identification of clusters. This approach revealed groups of related genetic alterations sharing similar mutational patterns among the prediction targets.

Subsequently, we employed association rule mining[4] to identify patterns of co-occurrence among gene mutations and conditions, such as MSI. This method evaluates the relationship between an initiating genetic alteration, referred to as the ‘antecedent’, and a potentially resulting alteration, termed the ‘consequent’, using several established metrics. Support[5] quantifies the frequency with which the antecedent and consequent co-occur within the dataset, while confidence[5] measures the conditional probability of observing the consequent given the presence of the antecedent, thereby reflecting the predictive strength of the association. Lift[5] assesses the degree of association by comparing the observed frequency of co-occurrence to what would be expected under statistical independence. Leverage[6] calculates the difference between the observed co-occurrence frequency and the expected frequency, highlighting the actual impact of the association. Conviction[7] gauges the reliability of the rule, with higher values indicating greater dependence of the consequent on the antecedent. Zhang’s Metric[8] further evaluates the relevance of the relationship by accounting for both the presence and absence of the antecedent and consequent, offering a more balanced assessment of the association's significance. These metrics must be interpreted cautiously and in conjunction to ensure meaningful conclusions, particularly given the inherent complexity of biological data and potential limitations arising from missing or sparse data.

Supplementary Tables

Tab. S1: Sociodemographic and clinicopathological patient characteristics of all cohorts. We selected relevant and representative targets from all targets within the study as described in the manuscript. The use of the dataset as a train or test dataset is also indicated. 'N' denotes quantity, 'IQR' is interquartile range, 'WT' is wild type, 'MUT' is mutated, and 'NaN' indicates missing information.

		**GECCO (primary dataset)**					**Public Cohorts**	
		**EPIC**	**CORSA**	**IWHS**	**CRA**	**WHI**	**TCGA**	**CPTAC**
Dataset		train	train	train	test	test	test	test
N Patients		183	158	390	321	324	426	110
Age	Median	62	69	63	67	65	67	66
	IQR	13	16	6	15	10	18	18
	<50	17 (9%)	10 (6%)	0 (0%)	29 (9%)	0 (0%)	59 (14%)	5 (5%)
	NaN	0 (0%)	1 (1%)	0 (0%)	0 (0%)	0 (0%)	1 (0%)	2 (2%)
Sex	Male	83 (45%)	99 (63%)	0 (0%)	183 (57%)	0 (0%)	212 (50%)	44 (40%)
	Female	100 (55%)	59 (37%)	**390 (100%)**	138 (43%)	**324 (100%)**	213 (50%)	66 (60%)
	NaN	0 (0%)	0 (0%)	0 (0%)	0 (0%)	0 (0%)	1 (0%)	0 (0%)
Race	White	183 (100%)	158 (100%)	385 (99%)	106 (33%)	292 (90%)	244 (57%)	81 (74%)
	Black/African-American	0 (0%)	0 (0%)	0 (0%)	4 (1%)	14 (4%)	57 (13%)	7 (6%)
	American Indian/Alaska Native	0 (0%)	0 (0%)	0 (0%)	0 (0%)	1 (0%)	1 (0%)	1 (1%)
	Asian	0 (0%)	0 (0%)	0 (0%)	0 (0%)	3 (1%)	12 (3%)	18 (16%)
	NaN	0 (0%)	0 (0%)	5 (1%)	211 (66%)	14 (4%)	112 (26%)	3 (3%)
Cancer Site	Colon	96 (52%)	99 (63%)	323 (83%)	223 (69%)	287 (89%)	317 (74%)	110 (100%)
	Rectum	47 (26%)	50 (32%)	63 (16%)	98 (31%)	32 (10%)	105 (25%)	0 (0%)
	NaN	40 (22%)	9 (6%)	4 (1%)	0 (0%)	5 (2%)	4 (1%)	0 (0%)
T-Stage	T1	1 (1%)	11 (7%)	0 (0%)	23 (7%)	24 (7%)	14 (3%)	0 (0%)
	T2	1 (1%)	29 (18%)	0 (0%)	43 (13%)	56 (17%)	72 (17%)	17 (15%)
	T3	4 (2%)	95 (60%)	0 (0%)	172 (54%)	170 (52%)	292 (69%)	79 (72%)
	T4	3 (2%)	18 (11%)	0 (0%)	20 (6%)	68 (21%)	47 (11%)	14 (13%)
	TX	0 (0%)	1 (1%)	0 (0%)	2 (1%)	0 (0%)	0 (0%)	0 (0%)
	NaN	174 (95%)	4 (3%)	390 (100%)	61 (19%)	6 (2%)	1 (0%)	0 (0%)
M-Stage	M0	8 (4%)	72 (46%)	0 (0%)	214 (67%)	292 (90%)	311 (73%)	102 (93%)
	M1	1 (1%)	20 (13%)	0 (0%)	34 (11%)	26 (8%)	55 (13%)	7 (6%)
	MX	0 (0%)	0 (0%)	0 (0%)	12 (4%)	0 (0%)	54 (13%)	0 (0%)
	NaN	174 (95%)	66 (42%)	390 (100%)	61 (19%)	6 (2%)	6 (1%)	1 (1%)
N-Stage	N0	8 (4%)	74 (47%)	0 (0%)	157 (49%)	191 (59%)	235 (55%)	59 (54%)
	N1	1 (1%)	48 (30%)	0 (0%)	59 (18%)	68 (21%)	115 (27%)	35 (32%)
	N2	0 (0%)	27 (17%)	0 (0%)	43 (13%)	51 (16%)	74 (17%)	16 (15%)
	NX	0 (0%)	3 (2%)	0 (0%)	1 (0%)	0 (0%)	1 (0%)	0 (0%)
	NaN	174 (95%)	6 (4%)	390 (100%)	61 (19%)	14 (4%)	1 (0%)	0 (0%)
ACVR1B	WT	177 (97%)	150 (95%)	369 (95%)	306 (95%)	300 (93%)	405 (95%)	107 (97%)

		GECCO (primary dataset)					Public Cohorts	
		**EPIC**	**CORSA**	**IWHS**	**CRA**	**WHI**	**TCGA**	**CPTAC**
	MUT	6 (3%)	8 (5%)	21 (5%)	15 (5%)	24 (7%)	21 (5%)	3 (3%)
	NaN	0 (0%)	0 (0%)	0 (0%)	0 (0%)	0 (0%)	0 (0%)	0 (0%)
AKT1	WT	176 (96%)	150 (95%)	383 (98%)	310 (97%)	305 (94%)	417 (98%)	109 (99%)
	MUT	7 (4%)	8 (5%)	7 (2%)	11 (3%)	19 (6%)	9 (2%)	1 (1%)
	NaN	0 (0%)	0 (0%)	0 (0%)	0 (0%)	0 (0%)	0 (0%)	0 (0%)
ALK	WT	169 (92%)	148 (94%)	362 (93%)	295 (92%)	297 (92%)	398 (93%)	102 (93%)
	MUT	14 (8%)	10 (6%)	28 (7%)	26 (8%)	27 (8%)	28 (7%)	8 (7%)
	NaN	0 (0%)	0 (0%)	0 (0%)	0 (0%)	0 (0%)	0 (0%)	0 (0%)
APC	WT	64 (35%)	72 (46%)	163 (42%)	90 (28%)	174 (54%)	91 (21%)	27 (25%)
	MUT	119 (65%)	86 (54%)	227 (58%)	231 (72%)	150 (46%)	335 (79%)	83 (7y5%)
	NaN	0 (0%)	0 (0%)	0 (0%)	0 (0%)	0 (0%)	0 (0%)	0 (0%)
ATM	WT	162 (89%)	148 (94%)	331 (85%)	289 (90%)	297 (92%)	369 (87%)	101 (92%)
	MUT	21 (11%)	10 (6%)	59 (15%)	32 (10%)	27 (8%)	57 (13%)	9 (8%)
	NaN	0 (0%)	0 (0%)	0 (0%)	0 (0%)	0 (0%)	0 (0%)	0 (0%)
BMPR2	WT	157 (86%)	145 (92%)	324 (83%)	276 (86%)	263 (81%)	395 (93%)	96 (87%)
	MUT	26 (14%)	13 (8%)	66 (17%)	45 (14%)	61 (19%)	31 (7%)	14 (13%)
	NaN	0 (0%)	0 (0%)	0 (0%)	0 (0%)	0 (0%)	0 (0%)	0 (0%)
BRAF	WT	132 (72%)	148 (94%)	271 (69%)	242 (75%)	268 (83%)	370 (87%)	92 (84%)
	MUT	51 (28%)	10 (6%)	119 (31%)	79 (25%)	56 (17%)	56 (13%)	18 (16%)
	NaN	0 (0%)	0 (0%)	0 (0%)	0 (0%)	0 (0%)	0 (0%)	0 (0%)
CCDC40	WT	176 (96%)	152 (96%)	373 (96%)	312 (97%)	310 (96%)	411 (96%)	103 (94%)
	MUT	7 (4%)	6 (4%)	17 (4%)	9 (3%)	14 (4%)	15 (4%)	7 (6%)
	NaN	0 (0%)	0 (0%)	0 (0%)	0 (0%)	0 (0%)	0 (0%)	0 (0%)
CDK12	WT	178 (97%)	152 (96%)	378 (97%)	307 (96%)	309 (95%)	399 (94%)	107 (97%)
	MUT	5 (3%)	6 (4%)	12 (3%)	14 (4%)	15 (5%)	27 (6%)	3 (3%)
	NaN	0 (0%)	0 (0%)	0 (0%)	0 (0%)	0 (0%)	0 (0%)	0 (0%)
CHD1	WT	169 (92%)	0 (0%)	356 (91%)	310 (97%)	0 (0%)	404 (95%)	103 (94%)
	MUT	14 (8%)	0 (0%)	34 (9%)	11 (3%)	0 (0%)	22 (5%)	7 (6%)
	NaN	0 (0%)	158 (100%)	0 (0%)	0 (0%)	324 (100%)	0 (0%)	0 (0%)
CTNND1	WT	177 (97%)	156 (99%)	369 (95%)	310 (97%)	307 (95%)	406 (95%)	108 (98%)
	MUT	6 (3%)	2 (1%)	21 (5%)	11 (3%)	17 (5%)	20 (5%)	2 (2%)
	NaN	0 (0%)	0 (0%)	0 (0%)	0 (0%)	0 (0%)	0 (0%)	0 (0%)
DUSP16	WT	179 (98%)	155 (98%)	377 (97%)	312 (97%)	317 (98%)	425 (100%)	104 (95%)
	MUT	4 (2%)	3 (2%)	13 (3%)	9 (3%)	7 (2%)	1 (0%)	6 (5%)
	NaN	0 (0%)	0 (0%)	0 (0%)	0 (0%)	0 (0%)	0 (0%)	0 (0%)
ELL2	WT	176 (96%)	0 (0%)	378 (97%)	317 (99%)	0 (0%)	416 (98%)	105 (95%)
	MUT	7 (4%)	0 (0%)	12 (3%)	4 (1%)	0 (0%)	10 (2%)	5 (5%)
	NaN	0 (0%)	158 (100%)	0 (0%)	0 (0%)	324 (100%)	0 (0%)	0 (0%)
FHOD3	WT	164 (90%)	0 (0%)	335 (86%)	285 (89%)	0 (0%)	395 (93%)	96 (87%)
	MUT	19 (10%)	0 (0%)	55 (14%)	36 (11%)	0 (0%)	31 (7%)	14 (13%)
	NaN	0 (0%)	158 (100%)	0 (0%)	0 (0%)	324 (100%)	0 (0%)	0 (0%)
Hypermutated (HM)	not HM	143 (78%)	127 (80%)	286 (73%)	254 (79%)	213 (66%)	358 (84%)	0 (0%)
	HM	40 (22%)	31 (20%)	104 (27%)	67 (21%)	111 (34%)	68 (16%)	0 (0%)
	NaN	0 (0%)	0 (0%)	0 (0%)	0 (0%)	0 (0%)	0 (0%)	110 (100%)
KIF1A	WT	163 (89%)	135 (85%)	353 (91%)	286 (89%)	296 (91%)	399 (94%)	101 (92%)
	MUT	20 (11%)	23 (15%)	37 (9%)	35 (11%)	28 (9%)	27 (6%)	9 (8%)
	NaN	0 (0%)	0 (0%)	0 (0%)	0 (0%)	0 (0%)	0 (0%)	0 (0%)
KRAS	WT	124 (68%)	101 (64%)	266 (68%)	205 (64%)	226 (70%)	238 (56%)	74 (67%)

		GECCO (primary dataset)					Public Cohorts	
		**EPIC**	**CORSA**	**IWHS**	**CRA**	**WHI**	**TCGA**	**CPTAC**
	MUT	59 (32%)	57 (36%)	124 (32%)	116 (36%)	98 (30%)	188 (44%)	36 (33%)
	NaN	0 (0%)	0 (0%)	0 (0%)	0 (0%)	0 (0%)	0 (0%)	0 (0%)
MECOM	WT	173 (95%)	0 (0%)	350 (90%)	295 (92%)	0 (0%)	403 (95%)	102 (93%)
	MUT	10 (5%)	0 (0%)	40 (10%)	26 (8%)	0 (0%)	23 (5%)	8 (7%)
	NaN	0 (0%)	158 (100%)	0 (0%)	0 (0%)	324 (100%)	0 (0%)	0 (0%)
MSS/ MSI	MSS	143 (78%)	147 (93%)	283 (73%)	256 (80%)	212 (65%)	365 (86%)	84 (76%)
	MSI	40 (22%)	11 (7%)	107 (27%)	65 (20%)	112 (35%)	61 (14%)	25 (23%)
	NaN	0 (0%)	0 (0%)	0 (0%)	0 (0%)	0 (0%)	0 (0%)	1 (1%)
NRAS	WT	174 (95%)	148 (94%)	376 (96%)	305 (95%)	312 (96%)	395 (93%)	103 (94%)
	MUT	9 (5%)	10 (6%)	14 (4%)	16 (5%)	12 (4%)	31 (7%)	7 (6%)
	NaN	0 (0%)	0 (0%)	0 (0%)	0 (0%)	0 (0%)	0 (0%)	0 (0%)
PIK3CA	WT	148 (81%)	143 (91%)	291 (75%)	262 (82%)	278 (86%)	307 (72%)	87 (79%)
	MUT	35 (19%)	15 (9%)	99 (25%)	59 (18%)	46 (14%)	119 (28%)	23 (21%)
	NaN	0 (0%)	0 (0%)	0 (0%)	0 (0%)	0 (0%)	0 (0%)	0 (0%)
PLEKHA6	WT	169 (92%)	0 (0%)	346 (89%)	292 (91%)	0 (0%)	402 (94%)	102 (93%)
	MUT	14 (8%)	0 (0%)	44 (11%)	29 (9%)	0 (0%)	24 (6%)	8 (7%)
	NaN	0 (0%)	158 (100%)	0 (0%)	0 (0%)	324 (100%)	0 (0%)	0 (0%)
RFX5	WT	176 (96%)	0 (0%)	369 (95%)	308 (96%)	0 (0%)	411 (96%)	105 (95%)
	MUT	7 (4%)	0 (0%)	21 (5%)	13 (4%)	0 (0%)	15 (4%)	5 (5%)
	NaN	0 (0%)	158 (100%)	0 (0%)	0 (0%)	324 (100%)	0 (0%)	0 (0%)
RNF43	WT	149 (81%)	148 (94%)	294 (75%)	268 (83%)	234 (72%)	386 (91%)	93 (85%)
	MUT	34 (19%)	10 (6%)	96 (25%)	53 (17%)	90 (28%)	40 (9%)	17 (15%)
	NaN	0 (0%)	0 (0%)	0 (0%)	0 (0%)	0 (0%)	0 (0%)	0 (0%)
SMAD2	WT	174 (95%)	153 (97%)	376 (96%)	306 (95%)	306 (94%)	404 (95%)	100 (91%)
	MUT	9 (5%)	5 (3%)	14 (4%)	15 (5%)	18 (6%)	22 (5%)	10 (9%)
	NaN	0 (0%)	0 (0%)	0 (0%)	0 (0%)	0 (0%)	0 (0%)	0 (0%)
SMG1	WT	172 (94%)	150 (95%)	377 (97%)	308 (96%)	311 (96%)	408 (96%)	102 (93%)
	MUT	11 (6%)	8 (5%)	13 (3%)	13 (4%)	13 (4%)	18 (4%)	8 (7%)
	NaN	0 (0%)	0 (0%)	0 (0%)	0 (0%)	0 (0%)	0 (0%)	0 (0%)
TBX3	WT	174 (95%)	150 (95%)	376 (96%)	302 (94%)	306 (94%)	407 (96%)	102 (93%)
	MUT	9 (5%)	8 (5%)	14 (4%)	19 (6%)	18 (6%)	19 (4%)	8 (7%)
	NaN	0 (0%)	0 (0%)	0 (0%)	0 (0%)	0 (0%)	0 (0%)	0 (0%)
TGFBR2	WT	176 (96%)	152 (96%)	364 (93%)	304 (95%)	297 (92%)	411 (96%)	104 (95%)
	MUT	7 (4%)	6 (4%)	26 (7%)	17 (5%)	27 (8%)	15 (4%)	6 (5%)
	NaN	0 (0%)	0 (0%)	0 (0%)	0 (0%)	0 (0%)	0 (0%)	0 (0%)
TP53	WT	64 (35%)	57 (36%)	158 (41%)	109 (34%)	194 (60%)	150 (35%)	51 (46%)
	MUT	119 (65%)	101 (64%)	232 (59%)	212 (66%)	130 (40%)	276 (65%)	59 (54%)
	NaN	0 (0%)	0 (0%)	0 (0%)	0 (0%)	0 (0%)	0 (0%)	0 (0%)
TRPS1	WT	171 (93%)	0 (0%)	353 (91%)	293 (91%)	0 (0%)	381 (89%)	92 (84%)
	MUT	12 (7%)	0 (0%)	37 (9%)	28 (9%)	0 (0%)	45 (11%)	18 (16%)
	NaN	0 (0%)	158 (100%)	0 (0%)	0 (0%)	324 (100%)	0 (0%)	0 (0%)
WNT16	WT	175 (96%)	0 (0%)	371 (95%)	307 (96%)	0 (0%)	420 (99%)	99 (90%)
	MUT	8 (4%)	0 (0%)	19 (5%)	14 (4%)	0 (0%)	6 (1%)	11 (10%)
	NaN	0 (0%)	158 (100%)	0 (0%)	0 (0%)	324 (100%)	0 (0%)	0 (0%)
ZHX2	WT	174 (95%)	152 (96%)	381 (98%)	313 (98%)	310 (96%)	413 (97%)	106 (96%)
	MUT	9 (5%)	6 (4%)	9 (2%)	8 (2%)	14 (4%)	13 (3%)	4 (4%)

		GECCO (primary dataset)					Public Cohorts	
		**EPIC**	**CORSA**	**IWHS**	**CRA**	**WHI**	**TCGA**	**CPTAC**
	NaN	0 (0%)	0 (0%)	0 (0%)	0 (0%)	0 (0%)	0 (0%)	0 (0%)
ZNRF3	WT	162 (89%)	150 (95%)	340 (87%)	288 (90%)	287 (89%)	413 (97%)	101 (92%)
	MUT	21 (11%)	8 (5%)	50 (13%)	33 (10%)	37 (11%)	13 (3%)	9 (8%)
	NaN	0 (0%)	0 (0%)	0 (0%)	0 (0%)	0 (0%)	0 (0%)	0 (0%)

Tab. S2: List of included targets from data provided by GECCO. The table includes target names and explanations where clarification is required. These targets were used in both the primary and secondary models.

Target 1	Explanation 1	Target 2	Explanation 2	Target 3	Explanation 3
ABCA8		LIMCH1		SETD2	
ACVR1B		LMO7		SIN3A	
ACVR2A		LRRN3		SLC12A5	
AKAP7		MAMDC4		SLC1A3	
AKT1		MAML2		SMAD2	
ALK		MAP2K4		SMAD3	
AMER1		MAP2K7		SMAD4	
APC		MAST2		SMARCA4	
APC_NM_000038_truncated_first1600AA	Mutated yes/no. Truncating mutations within the first 1,600 amino acids for transcript NM000038 exclusively.	MBD6		SMG1	
ARID1A		MECOM		SOS1	
ARID1B		MLH1		SOX9	
ARID2		MLH3		SYNE1	
ARID3A		MMR	Pathway mutated yes/no in any genes:MLH1, MLH3, MSH2, MSH6, PMS2	SYT3	
ASXL1		MSH6		T2A	Number of T -> A and A -> T transversions
ATG2A		MSI		T2C	Number of T -> C and A -> G transitions/transversions
ATM		MTOR		T2G	Number of T -> G and A -> C transversions
ATXN1		MTUS2		TAF1L	
AXIN1		MUC4		TAF3	
AXIN2		MUTYH		TBX3	
B2M		MXRA5		TCERG1	
BCL9		MYH9		TCF7L2	
BCL9L		NCAPD3		TCHH	
BCOR		NFE2L3		TET1	
BIRC6		NLGN4X		TET2	
BMPR2		NOD2		TET3	
BRAF		NRAS		TEX14	
BRAF_NM_004333_V600	Mutated yes/no of all nonsynonymous SNVs in BRAF transcript NM_004333 at codon 600.	NRAS_NM_002524_Oncogenic_known	Mutated yes/no of all nonsynonymous SNVs in NRAS transcript NM_002524 codons 12,13 and 61.	TGFBR1	
C2A	Number of C -> A and G -> T transversions	NRG1		TGFBR2	
C2G	Number of C -> G and G -> C transversions	OSBPL6		TGF_beta	Pathway mutated yes/no in any genes: ACVR1B, ACVR2A, BMPR1A, BMPR2, GDF5, SMAD2, SMAD3, SMAD4, TGFBR1, TGFBR2
C2T	Number of C -> T and G -> A transitions	PATIENT		TGIF1	
CACNG3		PAX5		TLR9	
CALD1		PBRM1		TNRC6B	
CASP8		PCBP1		TP53	
CCDC13		PCDH10		TP53BP1	
CCDC40		PCDHA3		TP53_NM_000546_non_silent	TP53_NM_000546_Oncogenic_known or TP53_NM_000546_other_non_silent mutated yes/no.
CDH1		PCDHGA7		TPR	
CDK12		PCDHGA9		TRPS1	
CDKN2A		PCDHGB1		TSHZ2	
CHD1		PIK3CA		TYRO3	
CHD4		PIK3CA_NM_006218_nonsynonymous_SNV	Mutated yes/no of all nonsynonymous SNVs in transcript NM_006218 (PIK3CA).	USP9X	
CPEB2		PIK3R1		UTP20	
CRTC1		PIK3R1_NM_181523_non_silent	Mutated yes/no of all non-silent mutations called for PIK3R1 transcript NM_181523.	WNT	Pathway mutated yes/no in any genes: AMER1, APC, ARID1A, AXIN1, AXIN2, CTNNB1, FBXW7, RNF43, SOX9, TCF7, TCF7L2, ZNRF3
CSMD1		PLEKHA6		WNT16	
CTCF		PLK1		XPO6	
CTNNB1		POLD1		XYLT2	
CTNND1		POLE		ZBTB20	
CUX1		POLQ		ZBTB7A	
DAB2		PTEN		ZDHHC8	

DAPK1		PTEN_NM_000314_non_silent	Mutated yes/no of all non-silent mutations called for PTEN transcript NM_000314.	ZFHX3	
DCAF4L1		RB1		ZFP36L2	
DCC		RBM10		ZHX2	
DCHS1		RECQL5		ZNF512B	
DNMT1		RFX5		ZNF521	
DOCK3		RGMB		ZNRF3	
DPYD		RGS12		alcohol_ref	Alcohol use, at the reference time
DUSP16		RNF43		alcoholc2	Alcohol use (nondrinker vs 1-28g/day)
DYNC1H1		RNF43_NM_001305544_truncating	Mutated yes/no of all truncating mutations called for transcript NM_001305544.	alcoholc2_1-28g/d	
ELF3		RTK_RAS	Pathway mutated yes/no in any genes:BRAF, ERBB2, ERBB3, KRAS, NRAS	alcoholc2_nondrinker	
ELL2		RTK_RAS_EM_TS_combined		asp_ref(2)	Regular aspirin/NSAID use at referent time, definition1
ELMO1		RYR1		asp_ref_missing	
ENAM		S1		aspirin	Aspirin use?
EP300		S10		aspirin_ever	Aspirin, ever used regularly?
EP400		S11		diab	Ever diagnosed with diabetes by a doctor?
ERBB2		S12		horm_ref	Any postmenopausal hormone use?
ERBB3		S14		hrt_ref_pm(x)	Any Post-menopausal HRT use at reference time, definition1
ERCC5		S15		hypermutated	
ESR1		S16		log_n_snv	Log transformed number of SNVs called from tumor-normal bam pair
FAN1		S17		log_total_mutations	Log transformed total number of SNVs and InDels mutations
FAT1		S18		n_indel	Total number of InDels mutations
FBLN2		S19		n_snv	Total number of SNVs called from tumor-normal bam pair (only pass-filter SNVs)
FBXW7		S1PR4		non_silent_2_silent_ratio	Non-silent to silent ratio.
FGFR1		S20		nsaids	Non-aspirin NSAIDS use?
FHOD3		S21		nsaids_ever	Non-aspirin NSAIDS, ever used regularly?
GDF5		S22		p53	Pathway mutated yes/no in any genes: ATM, TP53
GNAS		S23		smk_ever	Ever smoked cigarettes?
GPATCH8		S24		smoke	Smoking status
GPC5		S25		smoke_Former smoker	
HCN1		S26		smoke_Never smoker	
HGF		S28		smoke_Smoker	
IGF2_PI3K	Pathway mutated yes/no in any genes: IGF2, PIK3CA, PIK3R1, PTEN	S29		white_nonhisp	Non-Hispanic white
ING1		S3			
KDM6A		S30			
KIF1A		S4			
KLF3		S5			
KMT2B		S6			
KMT2C		S7			
KMT2D		S8			
KRAS		S9			
KRAS_NM_033360_Oncogenic_known	Mutated yes/no of all nonsynonymous SNVs in KRAS transcript NM_033360 codons 12, 13, 61, 117, 146.	SALL4			
		SCN5A			

Tab. S3: Links to directories containing the code used for the study and trained models.

Process	Link
Tessellation	https://github.com/KatherLab/preprocessing-ng/tree/1f5fdebf669363cf67bb422bb4cb0f91218d9c29
Feature extraction with CTransPath	https://github.com/KatherLab/marugoto/tree/d401e1157635273cd4a99ca6e60c83db7ea09a22
Multi-Target Transformer model, Heatmaps	https://github.com/gustavmarco/barspoon-transformer/tree/08b30a6cf80c8ed44c59fecb5a367b307da3284a
Top-tiles	https://github.com/LocalToasty/barspoon-transformer/tree/276d288cfeaaebf44c4d7f4e03dfe003c5bc7bc3
Trained models from 7 folds	https://github.com/gustavmarco/barspoon-transformer/releases/tag/gustav2024

Tab. S4: Comparative analysis of the internal and external performance of Multi-Target and Single-Target Transformers. The performance is evaluated using the mean Area Under Precision Recall Curve (AUROC) from the 7 folds of the cross-validation for relevant selected prediction targets. AUROCs greater than 0·75 are highlighted in bold. The table includes results for the Single-Target Transformer for high relevance prediction targets from genetic Clusters 1-2 (Fig. 2). A two-sided DeLong test was conducted, indicating the fold results of Multi-Target versus Single-Target Transformers.

	**Multi-Target Transformer**			**Single-Target Transformer**			**DeLong test p-value**
**Target**	**AUROC$_{internal}$**	**Mean (std) AUROC$_{external}$**	**Median AUROC$_{external}$**	**AUROC$_{internal}$**	**Mean (std) AUROC$_{external}$**	**Median AUROC$_{external}$**	
ACVR1B	0·68	**0·75 (± 0·03)**	**0·77**				
AKT1	**0·75**	0·70 (± 0·03)	0·70				
ALK	0·63	0·70 (± 0·02)	0·71				
APC	0·6	0·66 (± 0·02)	0·67	0·58	0·65 (± 0·03)	0·66	0·7142
ATM	0·59	0·63 (± 0·01)	0·63				
BMPR2	**0·82**	**0·87 (± 0·01)**	**0·87**	**0·80**	**0·81 (± 0·03)**	**0·82**	0·0001
BRAF	**0·79**	**0·78 (± 0·01)**	**0·78**	**0·75**	**0·72 (± 0·06)**	**0·75**	<0·0001
CCDC40	0·68	0·76 (± 0·04)	**0·78**				
CDK12	0·69	0·78 (± 0·02)	**0·78**				
CHD1	**0·78**	**0·87 (± 0·02)**	**0·88**				
CTNND1	**0·75**	0·75 (± 0·03)	0·73				
DUSP16	0·61	0·74 (± 0·04)	0·74				
ELL2	**0·76**	**0·79 (± 0·05)**	**0·81**				
FHOD3	**0·77**	**0·84 (± 0·02)**	**0·84**				
hypermu-tated	**0·80**	**0·88 (± 0·01)**	**0·88**	**0·82**	**0·86 (± 0·03)**	**0·87**	0·2383
KIF1A	0·67	0·67 (± 0·03)	0·67				
KRAS	0·63	0·65 (± 0·03)	0·65	0·61	0·65 (± 0·02)	0·65	0·5590
MECOM	**0·76**	**0·78 (± 0·04)**	**0·78**				
MSI	**0·84**	**0·93 (± 0·01)**	**0·94**	**0·87**	**0·91 (± 0·02)**	**0·91**	0·0015
NRAS	0·52	0·56 (± 0·04)	0·55				
PIK3CA	0·61	0·56 (± 0·03)	0·55				
PLEKHA6	0·74	**0·90 (± 0·01)**	**0·90**				
RFX5	**0·79**	**0·77 (± 0·02)**	**0·77**				
RNF43	**0·81**	**0·86 (± 0·01)**	**0·86**	**0·80**	**0·80 (± 0·05)**	**0·83**	0·0021
SMAD2	0·50	0·65 (± 0·03)	0·65				
SMG1	0·58	**0·76 (± 0·07)**	**0·78**				
TBX3	0·58	0·74 (± 0·03)	0·73				
TGFBR2	0·67	**0·77 (± 0·02)**	**0·77**				
TP53	0·65	0·72 (± 0·02)	0·72	0·64	0·69 (± 0·05)	0·70	0·3747
TRPS1	0·63	0·73 (± 0·03)	0·72				
WNT16	**0·75**	**0·82 (± 0·02)**	**0·82**				
ZHX2	0·66	0·74 (± 0·02)	0·74				
ZNRF3	**0·78**	**0·75 (± 0·01)**	**0·75**	0·67	0·67 (± 0·04)	0·68	0·0042

Tab. S5: Performance metrics of Multi-Target Transformers including MSI as a target (primary model) for external validation on CRA. Results are presented as mean and standard deviation across seven cross-validation folds for selected prediction targets. Metrics include Matthews Correlation Coefficient (MCC), Area Under the Receiver Operating Characteristic Curve (AUROC), and Area Under the Precision-Recall Curve (AUPRC), with mutation rates in the cohort. Binary classification thresholds were pre-defined at 0·5. Data is sorted by AUROC.

**Target**	**Accuracy**	**Precision**	**Sensitivity**	**Specificity**	**F1 Score**	**MCC**	**AUROC**	**AUPRC**	**Mutation Rate**	**(Target MUT + MSI) / Target MUT**
MSI	0·85 (±0·05)	0·61 (±0·11)	0·85 (±0·09)	0·85 (±0·08)	0·70 (±0·06)	0·63 (±0·08)	0·92 (±0·02)	0·75 (±0·06)	0·20	1·00
hypermutated	0·79 (±0·08)	0·52 (±0·10)	0·88 (±0·06)	0·77 (±0·11)	0·65 (±0·07)	0·56 (±0·08)	0·90 (±0·01)	0·73 (±0·05)	0·21	0·97
PLEKHA6	0·77 (±0·05)	0·28 (±0·04)	0·91 (±0·08)	0·76 (±0·06)	0·42 (±0·04)	0·42 (±0·03)	0·90 (±0·01)	0·38 (±0·06)	0·09	0·97
BMPR2	0·80 (±0·06)	0·41 (±0·07)	0·87 (±0·10)	0·78 (±0·08)	0·55 (±0·06)	0·50 (±0·06)	0·89 (±0·02)	0·48 (±0·02)	0·14	0·98
CHD1	0·76 (±0·07)	0·12 (±0·03)	0·84 (±0·15)	0·76 (±0·08)	0·20 (±0·03)	0·26 (±0·02)	0·87 (±0·02)	0·17 (±0·06)	0·03	0·91
RNF43	0·80 (±0·04)	0·45 (±0·06)	0·74 (±0·10)	0·81 (±0·06)	0·55 (±0·03)	0·46 (±0·04)	0·87 (±0·02)	0·48 (±0·02)	0·17	0·81
FHOD3	0·79 (±0·06)	0·33 (±0·06)	0·77 (±0·14)	0·79 (±0·08)	0·45 (±0·04)	0·40 (±0·04)	0·84 (±0·02)	0·38 (±0·03)	0·11	0·81
BRAF	0·80 (±0·02)	0·59 (±0·07)	0·66 (±0·10)	0·84 (±0·05)	0·61 (±0·03)	0·48 (±0·02)	0·83 (±0·02)	0·61 (±0·03)	0·25	0·65
CDK12	0·64 (±0·09)	0·09 (±0·01)	0·84 (±0·13)	0·63 (±0·10)	0·17 (±0·02)	0·20 (±0·04)	0·82 (±0·03)	0·18 (±0·06)	0·04	0·86
WNT16	0·77 (±0·08)	0·14 (±0·04)	0·76 (±0·14)	0·77 (±0·09)	0·24 (±0·05)	0·26 (±0·04)	0·82 (±0·02)	0·18 (±0·02)	0·04	0·93
SMG1	0·67 (±0·11)	0·10 (±0·03)	0·80 (±0·16)	0·67 (±0·12)	0·17 (±0·04)	0·20 (±0·04)	0·81 (±0·03)	0·14 (±0·02)	0·04	0·77
ZNRF3	0·77 (±0·04)	0·28 (±0·04)	0·75 (±0·06)	0·77 (±0·05)	0·41 (±0·04)	0·36 (±0·04)	0·81 (±0·02)	0·32 (±0·03)	0·10	0·91
AKT1	0·57 (±0·22)	0·08 (±0·04)	0·82 (±0·14)	0·57 (±0·23)	0·14 (±0·07)	0·16 (±0·11)	0·79 (±0·08)	0·14 (±0·05)	0·03	0·82
ELL2	0·71 (±0·08)	0·03 (±0·00)	0·75 (±0·14)	0·71 (±0·09)	0·06 (±0·01)	0·11 (±0·01)	0·79 (±0·05)	0·06 (±0·01)	0·01	0·75
DUSP16	0·46 (±0·21)	0·05 (±0·02)	0·89 (±0·13)	0·45 (±0·22)	0·09 (±0·03)	0·12 (±0·06)	0·78 (±0·08)	0·11 (±0·04)	0·03	0·78
MECOM	0·75 (±0·05)	0·20 (±0·04)	0·70 (±0·09)	0·75 (±0·06)	0·31 (±0·05)	0·28 (±0·07)	0·78 (±0·04)	0·21 (±0·04)	0·08	0·88
RFX5	0·71 (±0·09)	0·11 (±0·02)	0·77 (±0·11)	0·71 (±0·10)	0·18 (±0·03)	0·21 (±0·04)	0·77 (±0·02)	0·12 (±0·01)	0·04	0·85
ACVR1B	0·63 (±0·12)	0·09 (±0·02)	0·73 (±0·09)	0·62 (±0·13)	0·16 (±0·03)	0·16 (±0·05)	0·74 (±0·04)	0·22 (±0·06)	0·05	0·53
CTNND1	0·70 (±0·08)	0·08 (±0·02)	0·68 (±0·05)	0·71 (±0·08)	0·14 (±0·03)	0·15 (±0·05)	0·73 (±0·06)	0·11 (±0·03)	0·03	0·82
ALK	0·46 (±0·24)	0·13 (±0·04)	0·86 (±0·12)	0·43 (±0·27)	0·22 (±0·05)	0·17 (±0·08)	0·73 (±0·05)	0·22 (±0·05)	0·08	0·73
TGFBR2	0·65 (±0·10)	0·10 (±0·01)	0·69 (±0·17)	0·65 (±0·11)	0·18 (±0·02)	0·16 (±0·04)	0·73 (±0·03)	0·14 (±0·03)	0·05	0·65
TRPS1	0·48 (±0·17)	0·14 (±0·05)	0·80 (±0·07)	0·45 (±0·20)	0·23 (±0·07)	0·15 (±0·09)	0·73 (±0·03)	0·24 (±0·05)	0·09	0·68
CCDC40	0·65 (±0·12)	0·05 (±0·01)	0·67 (±0·20)	0·65 (±0·13)	0·10 (±0·02)	0·11 (±0·04)	0·72 (±0·07)	0·08 (±0·05)	0·03	0·67
TBX3	0·59 (±0·13)	0·11 (±0·01)	0·75 (±0·17)	0·58 (±0·14)	0·18 (±0·02)	0·17 (±0·03)	0·72 (±0·04)	0·15 (±0·05)	0·06	0·68
APC	0·68	0·81	0·75	0·52	0·75	0·28	0·71	0·85	0·72	0·12

	(±0·10)	(±0·05)	(±0·22)	(±0·24)	(±0·14)	(±0·05)	(±0·02)	(±0·01)		
TP53	0·62 (±0·03)	0·78 (±0·05)	0·61 (±0·16)	0·65 (±0·22)	0·67 (±0·07)	0·26 (±0·03)	0·69 (±0·02)	0·79 (±0·03)	0·66	0·10
KIF1A	0·47 (±0·19)	0·16 (±0·03)	0·80 (±0·15)	0·43 (±0·23)	0·26 (±0·03)	0·16 (±0·06)	0·67 (±0·04)	0·22 (±0·06)	0·11	0·57
ZHX2	0·59 (±0·08)	0·04 (±0·01)	0·62 (±0·23)	0·59 (±0·09)	0·07 (±0·02)	0·07 (±0·05)	0·67 (±0·03)	0·07 (±0·05)	0·02	0·62
KRAS	0·59 (±0·06)	0·46 (±0·07)	0·53 (±0·24)	0·62 (±0·21)	0·46 (±0·11)	0·17 (±0·07)	0·65 (±0·04)	0·51 (±0·05)	0·36	0·08
ATM	0·65 (±0·08)	0·16 (±0·03)	0·57 (±0·10)	0·66 (±0·10)	0·25 (±0·03)	0·15 (±0·05)	0·63 (±0·02)	0·19 (±0·03)	0·10	0·62
PIK3CA	0·55 (±0·10)	0·24 (±0·02)	0·66 (±0·20)	0·52 (±0·17)	0·34 (±0·04)	0·15 (±0·06)	0·62 (±0·03)	0·25 (±0·02)	0·18	0·39
SMAD2	0·44 (±0·16)	0·06 (±0·01)	0·70 (±0·15)	0·42 (±0·17)	0·11 (±0·02)	0·06 (±0·05)	0·61 (±0·06)	0·09 (±0·04)	0·05	0·33
NRAS	0·48 (±0·16)	0·05 (±0·01)	0·53 (±0·20)	0·48 (±0·17)	0·09 (±0·02)	0·00 (±0·05)	0·51 (±0·06)	0·06 (±0·02)	0·05	0·00

Tab. S6: Performance metrics of Multi-Target Transformers including MSI as a target (primary model) for external validation on WHI. Results are presented as mean and standard deviation across seven cross-validation folds for selected prediction targets. Metrics include Matthews Correlation Coefficient (MCC), Area Under the Receiver Operating Characteristic Curve (AUROC), and Area Under the Precision-Recall Curve (AUPRC), with mutation rates in the cohort. Binary classification thresholds were pre-defined at 0·5. Data is sorted by AUROC.

Target	Accuracy	Precision	Sensitivity	Specificity	F1 Score	MCC	AUROC	AUPRC	Mutation Rate	(Target MUT + MSI) / Target MUT
MSI	0·84 (±0·04)	0·73 (±0·07)	0·91 (±0·04)	0·81 (±0·07)	0·80 (±0·03)	0·69 (±0·05)	0·94 (±0·01)	0·91 (±0·02)	0·35	1.00
hypermutated	0·78 (±0·02)	0·64 (±0·04)	0·84 (±0·03)	0·75 (±0·05)	0·72 (±0·02)	0·56 (±0·03)	0·86 (±0·01)	0·76 (±0·02)	0·34	0·89
RNF43	0·75 (±0·04)	0·53 (±0·05)	0·86 (±0·03)	0·70 (±0·07)	0·66 (±0·03)	0·51 (±0·04)	0·84 (±0·01)	0·65 (±0·02)	0·28	0·89
BMPR2	0·71 (±0·05)	0·39 (±0·04)	0·90 (±0·07)	0·67 (±0·07)	0·54 (±0·02)	0·45 (±0·02)	0·84 (±0·01)	0·46 (±0·03)	0·19	0·97
TGFBR2	0·63 (±0·05)	0·17 (±0·01)	0·88 (±0·08)	0·60 (±0·06)	0·28 (±0·01)	0·27 (±0·02)	0·79 (±0·01)	0·21 (±0·02)	0·08	0·78
CCDC40	0·60 (±0·10)	0·08 (±0·01)	0·83 (±0·23)	0·59 (±0·12)	0·15 (±0·02)	0·17 (±0·05)	0·78 (±0·03)	0·15 (±0·07)	0·04	0·93
TBX3	0·47 (±0·16)	0·09 (±0·02)	0·91 (±0·05)	0·44 (±0·17)	0·17 (±0·03)	0·17 (±0·06)	0·78 (±0·03)	0·17 (±0·05)	0·06	0·83
ZHX2	0·61 (±0·05)	0·09 (±0·01)	0·91 (±0·09)	0·60 (±0·05)	0·17 (±0·01)	0·21 (±0·03)	0·78 (±0·00)	0·11 (±0·01)	0·04	0·86
BRAF	0·66 (±0·04)	0·32 (±0·02)	0·81 (±0·06)	0·63 (±0·06)	0·45 (±0·02)	0·34 (±0·02)	0·77 (±0·01)	0·41 (±0·04)	0·17	0·80
ACVR1B	0·60 (±0·06)	0·13 (±0·01)	0·80 (±0·17)	0·58 (±0·07)	0·23 (±0·02)	0·20 (±0·05)	0·75 (±0·04)	0·17 (±0·03)	0·07	0·50
CDK12	0·56 (±0·08)	0·09 (±0·01)	0·89 (±0·08)	0·54 (±0·09)	0·16 (±0·02)	0·18 (±0·03)	0·75 (±0·03)	0·13 (±0·02)	0·05	0·87
CTNND1	0·63 (±0·06)	0·11 (±0·02)	0·79 (±0·08)	0·62 (±0·07)	0·19 (±0·03)	0·19 (±0·04)	0·75 (±0·02)	0·11 (±0·02)	0·05	0·94
TP53	0·67 (±0·03)	0·60 (±0·05)	0·62 (±0·16)	0·70 (±0·15)	0·59 (±0·04)	0·33 (±0·03)	0·73 (±0·01)	0·64 (±0·03)	0·40	0·18
SMG1	0·48 (±0·18)	0·07 (±0·02)	0·85 (±0·17)	0·47 (±0·19)	0·12 (±0·04)	0·13 (±0·08)	0·72 (±0·10)	0·10 (±0·04)	0·04	0·77
DUSP16	0·45 (±0·18)	0·03 (±0·01)	0·76 (±0·14)	0·44 (±0·19)	0·06 (±0·01)	0·06 (±0·03)	0·70 (±0·05)	0·09 (±0·06)	0·02	0·71
SMAD2	0·38 (±0·19)	0·08 (±0·01)	0·88 (±0·09)	0·35 (±0·20)	0·14 (±0·02)	0·11 (±0·05)	0·70 (±0·02)	0·14 (±0·04)	0·06	0·44
ZNRF3	0·62 (±0·05)	0·19 (±0·02)	0·71 (±0·05)	0·61 (±0·07)	0·30 (±0·02)	0·21 (±0·03)	0·70 (±0·01)	0·23 (±0·02)	0·11	0·84
ALK	0·39 (±0·23)	0·11 (±0·02)	0·83 (±0·17)	0·35 (±0·26)	0·19 (±0·03)	0·11 (±0·06)	0·68 (±0·01)	0·16 (±0·02)	0·08	0·81
KIF1A	0·45 (±0·20)	0·12 (±0·02)	0·80 (±0·15)	0·42 (±0·23)	0·21 (±0·03)	0·13 (±0·05)	0·67 (±0·02)	0·19 (±0·02)	0·09	0·71
KRAS	0·62 (±0·05)	0·42 (±0·04)	0·61 (±0·13)	0·63 (±0·12)	0·49 (±0·04)	0·23 (±0·04)	0·66 (±0·02)	0·43 (±0·03)	0·30	0·08
AKT1	0·39 (±0·16)	0·08 (±0·01)	0·80 (±0·16)	0·37 (±0·18)	0·14 (±0·01)	0·09 (±0·03)	0·64 (±0·02)	0·09 (±0·01)	0·06	0·53
ATM	0·57 (±0·10)	0·13 (±0·02)	0·68 (±0·06)	0·55 (±0·11)	0·21 (±0·03)	0·13 (±0·05)	0·64 (±0·02)	0·14 (±0·02)	0·08	0·63
APC	0·57 (±0·03)	0·53 (±0·03)	0·59 (±0·24)	0·56 (±0·19)	0·53 (±0·18)	0·15 (±0·07)	0·60 (±0·02)	0·52 (±0·02)	0·46	0·21
NRAS	0·69 (±0·16)	0·05 (±0·02)	0·44 (±0·25)	0·70 (±0·18)	0·08 (±0·04)	0·05 (±0·05)	0·59 (±0·05)	0·09 (±0·06)	0·04	0·17
PIK3CA	0·43	0·15	0·66	0·39	0·25	0·04	0·51	0·15	0·14	0·46

	(±0·09)	(±0·01)	(±0·11)	(±0·12)	(±0·01)	(±0·03)	(±0·03)	(±0·02)		

Tab. S7: Performance metrics of Multi-Target Transformers including MSI as a target (primary model) for external validation on TCGA. Results are presented as mean and standard deviation across seven cross-validation folds for selected prediction targets. Metrics include Matthews Correlation Coefficient (MCC), Area Under the Receiver Operating Characteristic Curve (AUROC), and Area Under the Precision-Recall Curve (AUPRC), with mutation rates in the cohort. Binary classification thresholds were pre-defined at 0·5. Data is sorted by AUROC.

**Target**	**Accuracy**	**Precision**	**Sensitivity**	**Specificity**	**F1 Score**	**MCC**	**AUROC**	**AUPRC**	**Mutation Rate**	**(Target MUT + MSI) / Target MUT**
hypermutated	0·77 (±0·08)	0·42 (±0·09)	0·84 (±0·07)	0·75 (±0·11)	0·55 (±0·06)	0·47 (±0·06)	0·89 (±0·01)	0·63 (±0·02)	0·16	0·85
MSI	0·78 (±0·09)	0·40 (±0·10)	0·80 (±0·07)	0·77 (±0·11)	0·52 (±0·07)	0·46 (±0·07)	0·87 (±0·01)	0·62 (±0·02)	0·14	1.00
FHOD3	0·77 (±0·09)	0·23 (±0·06)	0·76 (±0·14)	0·77 (±0·10)	0·34 (±0·06)	0·32 (±0·05)	0·85 (±0·01)	0·34 (±0·04)	0·07	0·65
WNT16	0·75 (±0·07)	0·05 (±0·01)	0·79 (±0·13)	0·75 (±0·07)	0·09 (±0·03)	0·15 (±0·04)	0·84 (±0·03)	0·10 (±0·06)	0·01	0·67
ZHX2	0·73 (±0·04)	0·09 (±0·01)	0·82 (±0·11)	0·72 (±0·04)	0·16 (±0·03)	0·21 (±0·04)	0·83 (±0·05)	0·16 (±0·06)	0·03	0·69
ELL2	0·73 (±0·08)	0·07 (±0·01)	0·80 (±0·08)	0·73 (±0·09)	0·13 (±0·03)	0·18 (±0·04)	0·82 (±0·03)	0·15 (±0·07)	0·02	0·40
BMPR2	0·74 (±0·09)	0·20 (±0·05)	0·75 (±0·12)	0·74 (±0·11)	0·30 (±0·04)	0·29 (±0·04)	0·82 (±0·01)	0·31 (±0·03)	0·07	0·84
PLEKHA6	0·72 (±0·10)	0·15 (±0·05)	0·78 (±0·05)	0·71 (±0·11)	0·25 (±0·06)	0·25 (±0·06)	0·80 (±0·03)	0·22 (±0·04)	0·06	0·96
RNF43	0·73 (±0·08)	0·23 (±0·05)	0·72 (±0·09)	0·74 (±0·10)	0·35 (±0·05)	0·30 (±0·04)	0·80 (±0·01)	0·33 (±0·02)	0·09	0·75
MECOM	0·73 (±0·09)	0·14 (±0·03)	0·75 (±0·08)	0·73 (±0·10)	0·24 (±0·04)	0·25 (±0·04)	0·79 (±0·03)	0·17 (±0·03)	0·05	0·61
BRAF	0·73 (±0·07)	0·30 (±0·07)	0·72 (±0·07)	0·74 (±0·09)	0·42 (±0·05)	0·34 (±0·06)	0·78 (±0·02)	0·40 (±0·03)	0·13	0·64
ZNRF3	0·73 (±0·06)	0·08 (±0·02)	0·71 (±0·06)	0·73 (±0·06)	0·14 (±0·03)	0·17 (±0·04)	0·78 (±0·02)	0·12 (±0·05)	0·03	0·85
RFX5	0·72 (±0·11)	0·09 (±0·03)	0·65 (±0·13)	0·72 (±0·12)	0·15 (±0·03)	0·16 (±0·03)	0·75 (±0·02)	0·23 (±0·07)	0·04	0·80
CDK12	0·66 (±0·09)	0·12 (±0·03)	0·67 (±0·10)	0·66 (±0·10)	0·21 (±0·03)	0·17 (±0·03)	0·74 (±0·03)	0·21 (±0·05)	0·06	0·59
TGFBR2	0·67 (±0·09)	0·08 (±0·02)	0·68 (±0·06)	0·67 (±0·10)	0·13 (±0·04)	0·14 (±0·04)	0·74 (±0·03)	0·12 (±0·02)	0·04	0·60
DUSP16	0·52 (±0·20)	0·00 (±0·00)	0·86 (±0·38)	0·52 (±0·20)	0·01 (±0·01)	0·04 (±0·04)	0·73 (±0·18)	0·01 (±0·01)	0.00	1.00
TP53	0·63 (±0·06)	0·79 (±0·04)	0·59 (±0·15)	0·70 (±0·15)	0·67 (±0·08)	0·29 (±0·07)	0·72 (±0·03)	0·81 (±0·03)	0·65	0·08
ACVR1B	0·67 (±0·12)	0·10 (±0·03)	0·67 (±0·10)	0·67 (±0·13)	0·18 (±0·04)	0·16 (±0·04)	0·72 (±0·03)	0·15 (±0·07)	0·05	0·48
ALK	0·45 (±0·29)	0·11 (±0·05)	0·82 (±0·15)	0·43 (±0·32)	0·19 (±0·07)	0·14 (±0·09)	0·72 (±0·03)	0·19 (±0·03)	0·07	0·50
CTNND1	0·69 (±0·11)	0·10 (±0·03)	0·65 (±0·06)	0·69 (±0·12)	0·17 (±0·04)	0·16 (±0·04)	0·72 (±0·02)	0·14 (±0·03)	0·05	0·40
CCDC40	0·70 (±0·13)	0·08 (±0·02)	0·54 (±0·12)	0·71 (±0·14)	0·13 (±0·03)	0·11 (±0·04)	0·68 (±0·02)	0·10 (±0·03)	0·04	0·44
APC	0·68 (±0·11)	0·85 (±0·01)	0·73 (±0·17)	0·51 (±0·15)	0·77 (±0·11)	0·23 (±0·06)	0·67 (±0·01)	0·86 (±0·01)	0·79	0·08
CHD1	0·72 (±0·07)	0·09 (±0·01)	0·49 (±0·11)	0·73 (±0·08)	0·16 (±0·01)	0·11 (±0·02)	0·66 (±0·02)	0·09 (±0·01)	0·05	0·55
SMG1	0·65 (±0·17)	0·08 (±0·03)	0·59 (±0·18)	0·66 (±0·18)	0·13 (±0·05)	0·11 (±0·08)	0·64 (±0·11)	0·10 (±0·04)	0·04	0·50
ATM	0·66	0·22	0·55	0·68	0·30	0·17	0·64	0·24	0·13	0·37

	(±0·10)	(±0·03)	(±0·12)	(±0·13)	(±0·03)	(±0·04)	(±0·02)	(±0·03)		
NRAS	0·54 (±0·20)	0·09 (±0·04)	0·65 (±0·32)	0·54 (±0·24)	0·15 (±0·07)	0·09 (±0·06)	0·63 (±0·06)	0·11 (±0·03)	0·07	0·10
SMAD2	0·46 (±0·19)	0·07 (±0·01)	0·72 (±0·16)	0·44 (±0·21)	0·13 (±0·02)	0·08 (±0·03)	0·63 (±0·05)	0·09 (±0·02)	0·05	0·09
KIF1A	0·55 (±0·24)	0·09 (±0·02)	0·62 (±0·21)	0·55 (±0·27)	0·16 (±0·03)	0·09 (±0·05)	0·63 (±0·03)	0·11 (±0·03)	0·06	0·41
TRPS1	0·58 (±0·12)	0·16 (±0·03)	0·62 (±0·14)	0·58 (±0·15)	0·24 (±0·02)	0·13 (±0·03)	0·63 (±0·03)	0·19 (±0·04)	0·11	0·36
AKT1	0·60 (±0·16)	0·03 (±0·01)	0·59 (±0·17)	0·60 (±0·17)	0·06 (±0·02)	0·06 (±0·06)	0·62 (±0·11)	0·06 (±0·04)	0·02	0·67
TBX3	0·61 (±0·09)	0·06 (±0·01)	0·54 (±0·10)	0·62 (±0·10)	0·11 (±0·02)	0·07 (±0·04)	0·59 (±0·03)	0·08 (±0·02)	0·04	0·42
PIK3CA	0·47 (±0·08)	0·31 (±0·01)	0·71 (±0·16)	0·38 (±0·16)	0·43 (±0·03)	0·09 (±0·04)	0·58 (±0·03)	0·35 (±0·02)	0·28	0·20
KRAS	0·53 (±0·03)	0·48 (±0·04)	0·52 (±0·19)	0·55 (±0·20)	0·48 (±0·10)	0·07 (±0·05)	0·56 (±0·02)	0·52 (±0·03)	0·44	0·10

Tab. S8: Performance metrics of Multi-Target Transformers including MSI as a target (primary model) for external validation on CPTAC. Results are presented as mean and standard deviation across seven cross-validation folds for selected prediction targets. Metrics include Matthews Correlation Coefficient (MCC), Area Under the Receiver Operating Characteristic Curve (AUROC), and Area Under the Precision-Recall Curve (AUPRC), with mutation rates in the cohort. Binary classification thresholds were pre-defined at 0·5. Data is sorted by AUROC.

Target	Accuracy	Precision	Sensitivity	Specificity	F1 Score	MCC	AUROC	AUPRC	Mutation Rate	(Target MUT + MSI) / Target MUT
MSI	0·51 (±0·12)	0·32 (±0·06)	0·99 (±0·02)	0·36 (±0·16)	0·48 (±0·07)	0·33 (±0·11)	0·90 (±0·03)	0·75 (±0·05)	0·23	1.00
CCDC40	0·39 (±0·25)	0·11 (±0·04)	0·98 (±0·05)	0·35 (±0·27)	0·19 (±0·07)	0·18 (±0·11)	0·80 (±0·06)	0·26 (±0·08)	0·07	1.00
RNF43	0·32 (±0·08)	0·18 (±0·02)	0·98 (±0·05)	0·20 (±0·10)	0·30 (±0·03)	0·17 (±0·07)	0·80 (±0·03)	0·47 (±0·06)	0·15	0·88
BRAF	0·36 (±0·12)	0·18 (±0·03)	0·88 (±0·06)	0·27 (±0·15)	0·30 (±0·03)	0·13 (±0·06)	0·80 (±0·02)	0·53 (±0·08)	0·15	0·81
TGFBR2	0·34 (±0·17)	0·08 (±0·02)	0·95 (±0·08)	0·31 (±0·19)	0·15 (±0·03)	0·14 (±0·06)	0·79 (±0·08)	0·25 (±0·09)	0·06	1.00
FHOD3	0·42 (±0·16)	0·16 (±0·04)	0·89 (±0·10)	0·36 (±0·19)	0·27 (±0·04)	0·18 (±0·06)	0·78 (±0·01)	0·29 (±0·02)	0·11	0·92
PLEKHA6	0·25 (±0·11)	0·09 (±0·01)	0·96 (±0·06)	0·20 (±0·12)	0·17 (±0·02)	0·10 (±0·10)	0·76 (±0·10)	0·22 (±0·06)	0·08	0·88
BMPR2	0·37 (±0·08)	0·16 (±0·02)	0·99 (±0·03)	0·28 (±0·10)	0·28 (±0·02)	0·20 (±0·05)	0·76 (±0·03)	0·27 (±0·02)	0·12	0·85
ACVR1B	0·25 (±0·07)	0·03 (±0·01)	0·95 (±0·13)	0·22 (±0·07)	0·07 (±0·01)	0·07 (±0·06)	0·75 (±0·08)	0·29 (±0·22)	0·03	0·67
RFX5	0·33 (±0·22)	0·07 (±0·04)	0·94 (±0·10)	0·30 (±0·23)	0·13 (±0·06)	0·12 (±0·08)	0·75 (±0·05)	0·17 (±0·06)	0·05	1.00
KIF1A	0·20 (±0·11)	0·09 (±0·01)	0·98 (±0·05)	0·14 (±0·13)	0·16 (±0·02)	0·09 (±0·05)	0·75 (±0·05)	0·23 (±0·06)	0·08	0·88
TBX3	0·25 (±0·18)	0·10 (±0·03)	1·00 (±0·00)	0·19 (±0·20)	0·18 (±0·04)	0·12 (±0·10)	0·74 (±0·10)	0·25 (±0·10)	0·08	0·62
DUSP16	0·22 (±0·17)	0·07 (±0·01)	0·98 (±0·06)	0·18 (±0·18)	0·13 (±0·02)	0·09 (±0·07)	0·73 (±0·04)	0·15 (±0·06)	0·06	0·33
WNT16	0·35 (±0·15)	0·12 (±0·02)	0·89 (±0·09)	0·29 (±0·18)	0·21 (±0·04)	0·12 (±0·07)	0·73 (±0·03)	0·23 (±0·02)	0·09	0·80
ALK	0·23 (±0·19)	0·09 (±0·02)	0·91 (±0·16)	0·17 (±0·22)	0·16 (±0·02)	0·06 (±0·06)	0·72 (±0·09)	0·23 (±0·11)	0·08	0·75
CDK12	0·26 (±0·15)	0·04 (±0·01)	1·00 (±0·00)	0·24 (±0·15)	0·07 (±0·02)	0·09 (±0·04)	0·71 (±0·09)	0·10 (±0·02)	0·03	0·67
MECOM	0·32 (±0·11)	0·10 (±0·01)	0·96 (±0·06)	0·27 (±0·12)	0·18 (±0·02)	0·14 (±0·03)	0·71 (±0·09)	0·18 (±0·08)	0·08	0·88
ZNRF3	0·32 (±0·12)	0·10 (±0·02)	0·83 (±0·06)	0·27 (±0·13)	0·17 (±0·03)	0·06 (±0·06)	0·71 (±0·03)	0·30 (±0·06)	0·08	0·78
KRAS	0·70 (±0·02)	0·66 (±0·20)	0·23 (±0·14)	0·93 (±0·07)	0·32 (±0·14)	0·23 (±0·10)	0·69 (±0·06)	0·55 (±0·05)	0·33	0.00
SMG1	0·27 (±0·19)	0·08 (±0·01)	0·90 (±0·21)	0·23 (±0·21)	0·14 (±0·01)	0·08 (±0·05)	0·68 (±0·08)	0·16 (±0·06)	0·07	0·71
CHD1	0·33 (±0·12)	0·07 (±0·00)	0·88 (±0·16)	0·30 (±0·14)	0·13 (±0·01)	0·10 (±0·03)	0·68 (±0·04)	0·14 (±0·04)	0·06	0·83
APC	0·46 (±0·14)	0·83 (±0·04)	0·35 (±0·20)	0·81 (±0·08)	0·46 (±0·20)	0·15 (±0·13)	0·67 (±0·04)	0·83 (±0·02)	0·75	0·14
TRPS1	0·49 (±0·23)	0·21 (±0·05)	0·66 (±0·30)	0·46 (±0·33)	0·30 (±0·08)	0·10 (±0·09)	0·66 (±0·02)	0·32 (±0·07)	0·17	0·56
PIK3CA	0·45 (±0·15)	0·25 (±0·04)	0·78 (±0·23)	0·35 (±0·24)	0·38 (±0·06)	0·12 (±0·12)	0·64 (±0·04)	0·38 (±0·07)	0·22	0·43

SMAD2	0·15 (±0·04)	0·10 (±0·00)	1·00 (±0·00)	0·06 (±0·04)	0·18 (±0·01)	0·07 (±0·04)	0·62 (±0·05)	0·15 (±0·03)	0·09	0·40
ATM	0·28 (±0·11)	0·08 (±0·01)	0·88 (±0·22)	0·23 (±0·14)	0·15 (±0·02)	0·08 (±0·08)	0·59 (±0·08)	0·12 (±0·03)	0·08	0·50
TP53	0·54 (±0·03)	0·58 (±0·05)	0·43 (±0·24)	0·66 (±0·23)	0·46 (±0·19)	0·10 (±0·06)	0·59 (±0·01)	0·62 (±0·03)	0·53	0·14
AKT1	0·10 (±0·09)	0·01 (±0·00)	1·00 (±0·00)	0·09 (±0·09)	0·02 (±0·00)	0·03 (±0·02)	0·50 (±0·22)	0·02 (±0·01)	0·01	1.00
ELL2	0·18 (±0·09)	0·04 (±0·00)	0·79 (±0·09)	0·16 (±0·09)	0·07 (±0·01)	-0·05 (±0·09)	0·49 (±0·06)	0·09 (±0·09)	0·04	0·50
NRAS	0·81 (±0·16)	0·03 (±0·05)	0·07 (±0·13)	0·85 (±0·17)	0·03 (±0·06)	-0·05 (±0·07)	0·48 (±0·13)	0·07 (±0·03)	0·06	0·17
ZHX2	0·22 (±0·13)	0·04 (±0·01)	0·75 (±0·14)	0·20 (±0·13)	0·07 (±0·01)	-0·03 (±0·07)	0·46 (±0·05)	0·05 (±0·02)	0·04	0·50
CTNND1	0·38 (±0·18)	0·01 (±0·01)	0·43 (±0·45)	0·38 (±0·19)	0·02 (±0·02)	-0·05 (±0·08)	0·39 (±0·07)	0·02 (±0·00)	0·02	0.00

Tab. S9: Performance metrics of Multi-Target Transformers excluding MSI as a target (secondary model) for external validation on CRA. Results are presented as mean and standard deviation across seven cross-validation folds for selected prediction targets. Metrics include Matthews Correlation Coefficient (MCC), Area Under the Receiver Operating Characteristic Curve (AUROC), and Area Under the Precision-Recall Curve (AUPRC), with mutation rates in the cohort. Binary classification thresholds were pre-defined at 0·5. DeLong p-values, as detailed in Materials and Methods, compare performance to the primary model (Tab. S5). Data is sorted by AUROC.

Target	Accuracy	Precision	Sensitivity	Specificity	F1 Score	MCC	AUROC	AUPRC	Mutation Rate	(Target MUT + MSI) / Target MUT	DeLong test p-value
hypermutated	0·82 (±0·05)	0·58 (±0·11)	0·83 (±0·17)	0·82 (±0·09)	0·66 (±0·05)	0·58 (±0·05)	0·91 (±0·01)	0·74 (±0·04)	0·21	0·97	0·1534
PLEKHA6	0·79 (±0·09)	0·31 (±0·08)	0·85 (±0·15)	0·79 (±0·11)	0·44 (±0·07)	0·43 (±0·07)	0·90 (±0·02)	0·38 (±0·05)	0·09	0·97	0·6779
CHD1	0·78 (±0·07)	0·13 (±0·05)	0·86 (±0·16)	0·77 (±0·08)	0·22 (±0·06)	0·28 (±0·06)	0·89 (±0·04)	0·17 (±0·05)	0·03	0·91	0·1341
BMPR2	0·83 (±0·03)	0·45 (±0·06)	0·75 (±0·13)	0·84 (±0·06)	0·55 (±0·03)	0·49 (±0·04)	0·88 (±0·01)	0·47 (±0·03)	0·14	0·98	0·0929
RNF43	0·81 (±0·02)	0·46 (±0·03)	0·65 (±0·14)	0·85 (±0·05)	0·53 (±0·05)	0·44 (±0·05)	0·85 (±0·01)	0·48 (±0·03)	0·17	0·81	0·0613
FHOD3	0·81 (±0·04)	0·35 (±0·05)	0·73 (±0·13)	0·82 (±0·06)	0·47 (±0·03)	0·41 (±0·04)	0·84 (±0·01)	0·39 (±0·03)	0·11	0·81	0·3732
AKT1	0·64 (±0·22)	0·10 (±0·05)	0·83 (±0·26)	0·63 (±0·24)	0·16 (±0·06)	0·20 (±0·06)	0·82 (±0·07)	0·16 (±0·04)	0·03	0·82	0·1237
ELL2	0·77 (±0·08)	0·05 (±0·02)	0·79 (±0·17)	0·77 (±0·08)	0·09 (±0·03)	0·15 (±0·03)	0·82 (±0·06)	0·05 (±0·01)	0·01	0·75	0·5174
BRAF	0·79 (±0·01)	0·57 (±0·02)	0·64 (±0·08)	0·84 (±0·03)	0·60 (±0·03)	0·47 (±0·03)	0·82 (±0·03)	0·61 (±0·04)	0·25	0·65	0·1373
WNT16	0·79 (±0·07)	0·13 (±0·02)	0·65 (±0·23)	0·80 (±0·09)	0·21 (±0·04)	0·22 (±0·06)	0·81 (±0·03)	0·20 (±0·05)	0·04	0·93	0·9363
ZNRF3	0·77 (±0·06)	0·27 (±0·05)	0·68 (±0·09)	0·77 (±0·07)	0·38 (±0·04)	0·32 (±0·05)	0·80 (±0·02)	0·32 (±0·02)	0·1	0·91	0·0462
RFX5	0·79 (±0·06)	0·13 (±0·03)	0·65 (±0·15)	0·80 (±0·07)	0·21 (±0·04)	0·22 (±0·05)	0·78 (±0·05)	0·14 (±0·03)	0·04	0·85	0·6139
MECOM	0·77 (±0·06)	0·20 (±0·04)	0·57 (±0·17)	0·79 (±0·08)	0·29 (±0·04)	0·23 (±0·06)	0·77 (±0·02)	0·20 (±0·02)	0·08	0·88	0·0605
TRPS1	0·80 (±0·11)	0·28 (±0·09)	0·58 (±0·14)	0·82 (±0·14)	0·36 (±0·06)	0·30 (±0·06)	0·76 (±0·02)	0·30 (±0·03)	0·09	0·68	0·5339
DUSP16	0·61 (±0·15)	0·06 (±0·02)	0·79 (±0·23)	0·60 (±0·16)	0·11 (±0·03)	0·14 (±0·07)	0·74 (±0·11)	0·10 (±0·06)	0·03	0·78	0·8470
TBX3	0·62 (±0·23)	0·12 (±0·03)	0·70 (±0·21)	0·62 (±0·26)	0·20 (±0·04)	0·17 (±0·06)	0·74 (±0·04)	0·15 (±0·04)	0·06	0·68	0·8747
TGFBR2	0·73 (±0·07)	0·11 (±0·02)	0·55 (±0·13)	0·74 (±0·08)	0·18 (±0·02)	0·15 (±0·03)	0·74 (±0·02)	0·14 (±0·02)	0·05	0·65	0·6833
CDK12	0·69 (±0·10)	0·10 (±0·03)	0·69 (±0·20)	0·69 (±0·10)	0·17 (±0·05)	0·18 (±0·10)	0·73 (±0·14)	0·16 (±0·08)	0·04	0·86	0·0486
SMG1	0·52 (±0·25)	0·08 (±0·03)	0·84 (±0·08)	0·51 (±0·26)	0·15 (±0·06)	0·13 (±0·16)	0·72 (±0·18)	0·13 (±0·06)	0·04	0·77	0·2523
ALK	0·69 (±0·14)	0·17 (±0·03)	0·64 (±0·28)	0·70 (±0·17)	0·25 (±0·04)	0·20 (±0·06)	0·72 (±0·07)	0·20 (±0·02)	0·08	0·73	0·6855
ACVR1B	0·73 (±0·06)	0·11 (±0·02)	0·65 (±0·06)	0·73 (±0·06)	0·18 (±0·03)	0·18 (±0·04)	0·72 (±0·03)	0·23 (±0·05)	0·05	0·53	0·0493
APC	0·68 (±0·09)	0·81 (±0·05)	0·75 (±0·20)	0·52 (±0·22)	0·76 (±0·12)	0·28 (±0·05)	0·71 (±0·03)	0·85 (±0·02)	0·72	0·12	0·1261
KIF1A	0·58 (±0·09)	0·17 (±0·03)	0·71 (±0·07)	0·57 (±0·11)	0·28 (±0·03)	0·18 (±0·04)	0·70 (±0·04)	0·24 (±0·05)	0·11	0·57	0·1216

ZHX2	0·63 (±0·15)	0·04 (±0·01)	0·62 (±0·12)	0·63 (±0·16)	0·08 (±0·02)	0·09 (±0·03)	0·68 (±0·03)	0·06 (±0·02)	0·02	0·62	0·6861
TP53	0·58 (±0·10)	0·80 (±0·06)	0·51 (±0·25)	0·71 (±0·21)	0·58 (±0·21)	0·23 (±0·05)	0·68 (±0·02)	0·79 (±0·02)	0·66	0·10	0·2982
CCDC40	0·67 (±0·09)	0·05 (±0·01)	0·57 (±0·22)	0·68 (±0·10)	0·09 (±0·03)	0·09 (±0·06)	0·67 (±0·08)	0·06 (±0·01)	0·03	0·67	0·1356
CTNND1	0·73 (±0·14)	0·07 (±0·02)	0·52 (±0·17)	0·74 (±0·15)	0·13 (±0·04)	0·12 (±0·05)	0·67 (±0·06)	0·10 (±0·02)	0·03	0·82	0·0557
ATM	0·68 (±0·09)	0·16 (±0·02)	0·54 (±0·20)	0·70 (±0·13)	0·24 (±0·05)	0·15 (±0·06)	0·65 (±0·04)	0·18 (±0·03)	0·1	0·62	0·0495
SMAD2	0·64 (±0·10)	0·08 (±0·02)	0·57 (±0·17)	0·65 (±0·11)	0·13 (±0·04)	0·10 (±0·06)	0·64 (±0·07)	0·11 (±0·06)	0·05	0·33	0·0421
KRAS	0·50 (±0·03)	0·41 (±0·01)	0·84 (±0·06)	0·31 (±0·07)	0·55 (±0·02)	0·16 (±0·04)	0·63 (±0·02)	0·50 (±0·02)	0·36	0·08	0·0804
PIK3CA	0·60 (±0·10)	0·24 (±0·03)	0·55 (±0·24)	0·61 (±0·17)	0·32 (±0·07)	0·13 (±0·07)	0·60 (±0·03)	0·23 (±0·02)	0·18	0·39	0·0303
NRAS	0·63 (±0·18)	0·06 (±0·01)	0·42 (±0·23)	0·64 (±0·21)	0·10 (±0·02)	0·03 (±0·03)	0·57 (±0·08)	0·08 (±0·02)	0·05	0.00	0·0003

Tab. S10: Performance metrics of Multi-Target Transformers excluding MSI as a target (secondary model) for external validation on WHI. Results are presented as mean and standard deviation across seven cross-validation folds for selected prediction targets. Metrics include Matthews Correlation Coefficient (MCC), Area Under the Receiver Operating Characteristic Curve (AUROC), and Area Under the Precision-Recall Curve (AUPRC), with mutation rates in the cohort. Binary classification thresholds were pre-defined at 0·5. DeLong p-values, as detailed in Materials and Methods, compare performance to the primary model (Tab. S6). Data is sorted by AUROC.

**Target**	**Accuracy**	**Precision**	**Sensitivity**	**Specificity**	**F1 Score**	**MCC**	**AUROC**	**AUPRC**	**Mutation Rate**	**(Target MUT + MSI) / Target MUT**	**DeLong test p-value**
hypermutated	0·78 (±0·03)	0·66 (±0·06)	0·79 (±0·07)	0·78 (±0·07)	0·71 (±0·02)	0·55 (±0·03)	0·86 (±0·01)	0·76 (±0·00)	0·34	0·89	0·8738
BMPR2	0·74 (±0·03)	0·41 (±0·02)	0·84 (±0·08)	0·72 (±0·05)	0·55 (±0·01)	0·45 (±0·02)	0·84 (±0·01)	0·45 (±0·03)	0·19	0·97	0·5499
RNF43	0·77 (±0·04)	0·57 (±0·06)	0·80 (±0·07)	0·76 (±0·07)	0·66 (±0·02)	0·51 (±0·04)	0·84 (±0·01)	0·63 (±0·03)	0·28	0·89	0·7227
ZHX2	0·55 (±0·21)	0·09 (±0·02)	0·95 (±0·05)	0·53 (±0·22)	0·17 (±0·04)	0·20 (±0·08)	0·78 (±0·04)	0·12 (±0·03)	0·04	0·86	0·2049
TGFBR2	0·67 (±0·05)	0·17 (±0·01)	0·75 (±0·14)	0·66 (±0·07)	0·27 (±0·02)	0·24 (±0·05)	0·78 (±0·02)	0·20 (±0·02)	0·08	0·78	0·3054
TBX3	0·53 (±0·22)	0·11 (±0·03)	0·90 (±0·13)	0·51 (±0·24)	0·19 (±0·04)	0·20 (±0·08)	0·76 (±0·05)	0·18 (±0·05)	0·06	0·83	0·6648
ACVR1B	0·64 (±0·08)	0·15 (±0·02)	0·80 (±0·10)	0·62 (±0·09)	0·25 (±0·02)	0·23 (±0·03)	0·76 (±0·04)	0·19 (±0·05)	0·07	0·50	0·0459
BRAF	0·69 (±0·05)	0·33 (±0·03)	0·79 (±0·07)	0·66 (±0·07)	0·47 (±0·02)	0·35 (±0·03)	0·76 (±0·01)	0·42 (±0·01)	0·17	0·80	0·1273
CCDC40	0·59 (±0·12)	0·07 (±0·02)	0·71 (±0·16)	0·58 (±0·13)	0·13 (±0·03)	0·13 (±0·07)	0·74 (±0·07)	0·16 (±0·08)	0·04	0·93	0·2795
TP53	0·68 (±0·02)	0·62 (±0·04)	0·57 (±0·15)	0·76 (±0·10)	0·58 (±0·07)	0·34 (±0·04)	0·73 (±0·01)	0·63 (±0·01)	0·4	0·18	0·5195
CTNND1	0·68 (±0·09)	0·10 (±0·02)	0·65 (±0·25)	0·68 (±0·11)	0·17 (±0·04)	0·16 (±0·07)	0·72 (±0·06)	0·11 (±0·02)	0·05	0·94	0·1893
SMG1	0·46 (±0·18)	0·07 (±0·02)	0·87 (±0·16)	0·45 (±0·19)	0·12 (±0·03)	0·13 (±0·06)	0·71 (±0·11)	0·10 (±0·04)	0·04	0·77	0·2984
CDK12	0·66 (±0·13)	0·08 (±0·03)	0·65 (±0·31)	0·66 (±0·15)	0·13 (±0·06)	0·13 (±0·08)	0·69 (±0·12)	0·13 (±0·04)	0·05	0·87	0·1927
ZNRF3	0·63 (±0·06)	0·20 (±0·02)	0·74 (±0·06)	0·62 (±0·07)	0·32 (±0·02)	0·23 (±0·03)	0·69 (±0·02)	0·22 (±0·02)	0·11	0·84	0·8470
ALK	0·57 (±0·20)	0·13 (±0·02)	0·67 (±0·23)	0·56 (±0·24)	0·21 (±0·03)	0·14 (±0·05)	0·67 (±0·03)	0·15 (±0·01)	0·08	0·81	0·8384
KIF1A	0·58 (±0·11)	0·13 (±0·02)	0·67 (±0·13)	0·57 (±0·13)	0·22 (±0·02)	0·14 (±0·03)	0·67 (±0·01)	0·18 (±0·03)	0·09	0·71	0·6340
SMAD2	0·57 (±0·11)	0·09 (±0·01)	0·71 (±0·17)	0·56 (±0·12)	0·15 (±0·02)	0·12 (±0·05)	0·66 (±0·05)	0·10 (±0·02)	0·06	0·44	0·3794
KRAS	0·52 (±0·05)	0·37 (±0·01)	0·79 (±0·12)	0·41 (±0·12)	0·50 (±0·02)	0·20 (±0·03)	0·65 (±0·01)	0·41 (±0·01)	0·3	0·08	0·0342
ATM	0·60 (±0·13)	0·13 (±0·02)	0·65 (±0·13)	0·59 (±0·15)	0·22 (±0·02)	0·14 (±0·03)	0·64 (±0·02)	0·14 (±0·01)	0·08	0·63	0·8906
NRAS	0·68 (±0·23)	0·05 (±0·03)	0·46 (±0·33)	0·69 (±0·26)	0·09 (±0·04)	0·07 (±0·05)	0·62 (±0·04)	0·06 (±0·01)	0·04	0·17	0·1990
AKT1	0·56 (±0·16)	0·08 (±0·01)	0·59 (±0·23)	0·56 (±0·19)	0·13 (±0·02)	0·07 (±0·04)	0·62 (±0·03)	0·10 (±0·02)	0·06	0·53	0·2625
DUSP16	0·51 (±0·18)	0·03 (±0·01)	0·63 (±0·11)	0·50 (±0·19)	0·06 (±0·02)	0·04 (±0·06)	0·60 (±0·11)	0·05 (±0·02)	0·02	0·71	0·1360
APC	0·58 (±0·02)	0·54 (±0·02)	0·59 (±0·16)	0·57 (±0·12)	0·56 (±0·08)	0·17 (±0·06)	0·59 (±0·03)	0·52 (±0·03)	0·46	0·21	0·8926
PIK3CA	0·55 (±0·13)	0·13 (±0·03)	0·40 (±0·21)	0·58 (±0·19)	0·18 (±0·06)	-0·02 (±0·03)	0·51 (±0·04)	0·15 (±0·02)	0·14	0·46	0·6556

Tab. S11: Performance metrics of Multi-Target Transformers excluding MSI as a target (secondary model) for external validation on TCGA. Results are presented as mean and standard deviation across seven cross-validation folds for selected prediction targets. Metrics include Matthews Correlation Coefficient (MCC), Area Under the Receiver Operating Characteristic Curve (AUROC), and Area Under the Precision-Recall Curve (AUPRC), with mutation rates in the cohort. Binary classification thresholds were pre-defined at 0·5. DeLong p-values, as detailed in Materials and Methods, compare performance to the primary model (Tab. S7). Data is sorted by AUROC.

**Target**	**Accuracy**	**Precision**	**Sensitivity**	**Specificity**	**F1 Score**	**MCC**	**AUROC**	**AUPRC**	**Mutation Rate**	**(Target MUT + MSI) / Target MUT**	**DeLong test p-value**
hypermutated	0·77 (±0·07)	0·42 (±0·10)	0·82 (±0·10)	0·76 (±0·10)	0·54 (±0·05)	0·46 (±0·05)	0·88 (±0·01)	0·62 (±0·04)	0·16	0·85	0·7566
FHOD3	0·76 (±0·07)	0·21 (±0·04)	0·75 (±0·13)	0·76 (±0·09)	0·32 (±0·03)	0·30 (±0·02)	0·84 (±0·02)	0·33 (±0·05)	0·07	0·65	0·9096
BMPR2	0·77 (±0·06)	0·21 (±0·04)	0·72 (±0·09)	0·77 (±0·07)	0·32 (±0·04)	0·30 (±0·03)	0·83 (±0·01)	0·36 (±0·04)	0·07	0·84	0·2685
WNT16	0·76 (±0·10)	0·05 (±0·02)	0·69 (±0·12)	0·76 (±0·10)	0·08 (±0·03)	0·13 (±0·05)	0·80 (±0·06)	0·08 (±0·07)	0·01	0·67	0·0701
PLEKHA6	0·73 (±0·08)	0·15 (±0·03)	0·75 (±0·08)	0·72 (±0·09)	0·24 (±0·04)	0·25 (±0·03)	0·80 (±0·02)	0·21 (±0·03)	0·06	0·96	0·8039
ELL2	0·75 (±0·08)	0·05 (±0·01)	0·60 (±0·26)	0·76 (±0·09)	0·10 (±0·03)	0·12 (±0·06)	0·80 (±0·01)	0·11 (±0·04)	0·02	0·40	0·2476
MECOM	0·75 (±0·09)	0·15 (±0·02)	0·71 (±0·12)	0·75 (±0·10)	0·24 (±0·03)	0·24 (±0·02)	0·79 (±0·03)	0·17 (±0·03)	0·05	0·61	0·4972
BRAF	0·73 (±0·07)	0·30 (±0·05)	0·75 (±0·07)	0·73 (±0·08)	0·42 (±0·05)	0·34 (±0·05)	0·79 (±0·02)	0·40 (±0·03)	0·13	0·64	0·1631
RNF43	0·75 (±0·06)	0·23 (±0·03)	0·69 (±0·08)	0·75 (±0·07)	0·34 (±0·03)	0·29 (±0·03)	0·79 (±0·01)	0·32 (±0·03)	0·09	0·75	0·7658
ZHX2	0·65 (±0·20)	0·08 (±0·03)	0·80 (±0·14)	0·65 (±0·20)	0·14 (±0·04)	0·17 (±0·07)	0·78 (±0·08)	0·11 (±0·05)	0·03	0·69	0·1292
ZNRF3	0·69 (±0·10)	0·07 (±0·02)	0·73 (±0·08)	0·68 (±0·10)	0·13 (±0·03)	0·16 (±0·03)	0·77 (±0·02)	0·15 (±0·06)	0·03	0·85	0·5666
RFX5	0·76 (±0·09)	0·10 (±0·02)	0·67 (±0·11)	0·76 (±0·09)	0·17 (±0·03)	0·19 (±0·03)	0·76 (±0·01)	0·20 (±0·06)	0·04	0·80	0·5684
ALK	0·64 (±0·15)	0·14 (±0·05)	0·74 (±0·17)	0·63 (±0·17)	0·23 (±0·05)	0·20 (±0·04)	0·74 (±0·03)	0·19 (±0·04)	0·07	0·50	0·3049
TGFBR2	0·69 (±0·09)	0·08 (±0·02)	0·65 (±0·07)	0·69 (±0·10)	0·13 (±0·03)	0·14 (±0·04)	0·73 (±0·03)	0·14 (±0·04)	0·04	0·60	0·6144
ACVR1B	0·69 (±0·10)	0·10 (±0·02)	0·65 (±0·14)	0·69 (±0·11)	0·17 (±0·02)	0·16 (±0·03)	0·72 (±0·04)	0·15 (±0·05)	0·05	0·48	0·8886
TP53	0·59 (±0·08)	0·83 (±0·06)	0·49 (±0·19)	0·79 (±0·16)	0·59 (±0·15)	0·28 (±0·07)	0·72 (±0·03)	0·81 (±0·03)	0·65	0·08	0·2894
CDK12	0·67 (±0·09)	0·12 (±0·03)	0·63 (±0·15)	0·68 (±0·10)	0·20 (±0·05)	0·16 (±0·07)	0·70 (±0·07)	0·16 (±0·04)	0·06	0·59	0·1618
CCDC40	0·66 (±0·15)	0·07 (±0·02)	0·61 (±0·14)	0·66 (±0·16)	0·13 (±0·03)	0·11 (±0·04)	0·67 (±0·06)	0·13 (±0·04)	0·04	0·44	0·8089
APC	0·66 (±0·09)	0·84 (±0·01)	0·70 (±0·15)	0·52 (±0·13)	0·76 (±0·09)	0·20 (±0·05)	0·67 (±0·03)	0·87 (±0·01)	0·79	0·08	0·7125
CTNND1	0·68 (±0·13)	0·08 (±0·02)	0·54 (±0·17)	0·69 (±0·15)	0·14 (±0·03)	0·11 (±0·04)	0·66 (±0·05)	0·12 (±0·05)	0·05	0·40	0·0187
CHD1	0·72 (±0·08)	0·09 (±0·01)	0·51 (±0·17)	0·73 (±0·09)	0·16 (±0·02)	0·12 (±0·04)	0·66 (±0·01)	0·10 (±0·03)	0·05	0·55	0·7945
TRPS1	0·75 (±0·10)	0·20 (±0·04)	0·39 (±0·18)	0·79 (±0·13)	0·25 (±0·03)	0·15 (±0·04)	0·65 (±0·04)	0·22 (±0·02)	0·11	0·36	0·2477
AKT1	0·65 (±0·18)	0·05 (±0·02)	0·63 (±0·12)	0·65 (±0·19)	0·08 (±0·04)	0·10 (±0·05)	0·64 (±0·08)	0·08 (±0·08)	0·02	0·67	0·0020
ATM	0·63	0·22	0·58	0·64	0·30	0·17	0·64	0·24	0·13	0·37	0·2454

	(±0·16)	(±0·06)	(±0·18)	(±0·22)	(±0·03)	(±0·05)	(±0·02)	(±0·02)			
SMAD2	0·60 (±0·11)	0·07 (±0·02)	0·57 (±0·25)	0·60 (±0·13)	0·13 (±0·03)	0·08 (±0·06)	0·62 (±0·07)	0·08 (±0·02)	0·05	0·09	0·2060
KIF1A	0·64 (±0·08)	0·08 (±0·01)	0·48 (±0·09)	0·65 (±0·09)	0·14 (±0·01)	0·06 (±0·02)	0·62 (±0·02)	0·11 (±0·02)	0·06	0·41	0·6627
DUSP16	0·53 (±0·20)	0·00 (±0·00)	0·71 (±0·49)	0·53 (±0·20)	0·01 (±0·01)	0·02 (±0·06)	0·61 (±0·31)	0·01 (±0·01)	0.00	1.00	
NRAS	0·65 (±0·19)	0·09 (±0·03)	0·43 (±0·33)	0·67 (±0·22)	0·13 (±0·06)	0·05 (±0·08)	0·61 (±0·07)	0·11 (±0·03)	0·07	0·10	0·3766
SMG1	0·47 (±0·24)	0·06 (±0·02)	0·73 (±0·15)	0·46 (±0·25)	0·11 (±0·03)	0·08 (±0·05)	0·60 (±0·07)	0·08 (±0·03)	0·04	0·50	0·8932
TBX3	0·60 (±0·23)	0·07 (±0·02)	0·59 (±0·20)	0·60 (±0·24)	0·12 (±0·03)	0·08 (±0·05)	0·59 (±0·04)	0·09 (±0·05)	0·04	0·42	0·5554
PIK3CA	0·54 (±0·09)	0·33 (±0·02)	0·59 (±0·24)	0·52 (±0·22)	0·40 (±0·07)	0·11 (±0·03)	0·58 (±0·03)	0·34 (±0·02)	0·28	0·20	0·7623
KRAS	0·50 (±0·02)	0·46 (±0·01)	0·72 (±0·10)	0·32 (±0·11)	0·56 (±0·03)	0·04 (±0·02)	0·55 (±0·02)	0·50 (±0·03)	0·44	0·10	0·1642

Tab. S12: Performance metrics of Multi-Target Transformers excluding MSI as a target (secondary model) for external validation on CPTAC. Results are presented as mean and standard deviation across seven cross-validation folds for selected prediction targets. Metrics include Matthews Correlation Coefficient (MCC), Area Under the Receiver Operating Characteristic Curve (AUROC), and Area Under the Precision-Recall Curve (AUPRC), with mutation rates in the cohort. Binary classification thresholds were pre-defined at 0·5. DeLong p-values, as detailed in Materials and Methods, compare performance to the primary model (Tab. S8). Data is sorted by AUROC.

Target	Accuracy	Precision	Sensitivity	Specificity	F1 Score	MCC	AUROC	AUPRC	Mutation Rate	(Target MUT + MSI) / Target MUT	DeLong test p-value
CCDC40	0·36 (±0·17)	0·10 (±0·03)	0·96 (±0·07)	0·32 (±0·19)	0·17 (±0·05)	0·15 (±0·09)	0·81 (±0·06)	0·29 (±0·08)	0·07	1.00	0·3484
TGFBR2	0·28 (±0·13)	0·07 (±0·01)	0·98 (±0·06)	0·24 (±0·14)	0·14 (±0·02)	0·12 (±0·07)	0·80 (±0·04)	0·34 (±0·14)	0·06	1.00	0·7400
RNF43	0·38 (±0·13)	0·19 (±0·03)	0·93 (±0·07)	0·28 (±0·17)	0·32 (±0·04)	0·17 (±0·08)	0·80 (±0·03)	0·49 (±0·01)	0·15	0·88	0·6513
BRAF	0·39 (±0·15)	0·19 (±0·03)	0·91 (±0·06)	0·30 (±0·19)	0·32 (±0·04)	0·17 (±0·08)	0·80 (±0·03)	0·55 (±0·06)	0·15	0·81	0·5381
FHOD3	0·44 (±0·23)	0·18 (±0·07)	0·93 (±0·09)	0·37 (±0·27)	0·30 (±0·09)	0·22 (±0·12)	0·79 (±0·04)	0·31 (±0·04)	0·11	0·92	0·0786
RFX5	0·43 (±0·26)	0·08 (±0·03)	0·89 (±0·16)	0·41 (±0·28)	0·14 (±0·05)	0·14 (±0·07)	0·77 (±0·06)	0·16 (±0·05)	0·05	1.00	0·7903
BMPR2	0·43 (±0·14)	0·18 (±0·04)	0·95 (±0·09)	0·35 (±0·17)	0·30 (±0·05)	0·22 (±0·07)	0·77 (±0·03)	0·29 (±0·02)	0·12	0·85	0·3481
PLEKHA6	0·39 (±0·11)	0·10 (±0·02)	0·89 (±0·13)	0·35 (±0·13)	0·18 (±0·03)	0·14 (±0·08)	0·75 (±0·08)	0·21 (±0·05)	0·08	0·88	0·0501
ACVR1B	0·25 (±0·18)	0·04 (±0·01)	0·95 (±0·13)	0·23 (±0·18)	0·07 (±0·02)	0·07 (±0·06)	0·75 (±0·05)	0·18 (±0·11)	0·03	0·67	0·5133
KIF1A	0·25 (±0·20)	0·09 (±0·03)	0·96 (±0·09)	0·19 (±0·22)	0·17 (±0·04)	0·11 (±0·07)	0·74 (±0·04)	0·22 (±0·03)	0·08	0·88	0·3716
WNT16	0·42 (±0·18)	0·14 (±0·05)	0·89 (±0·09)	0·37 (±0·21)	0·24 (±0·06)	0·17 (±0·09)	0·73 (±0·03)	0·22 (±0·03)	0·09	0·80	0·9503
TBX3	0·23 (±0·10)	0·09 (±0·01)	1·00 (±0·00)	0·17 (±0·11)	0·17 (±0·02)	0·12 (±0·05)	0·72 (±0·08)	0·21 (±0·11)	0·08	0·62	0·1304
ZNRF3	0·31 (±0·15)	0·10 (±0·02)	0·84 (±0·13)	0·26 (±0·17)	0·18 (±0·03)	0·07 (±0·07)	0·72 (±0·03)	0·31 (±0·02)	0·08	0·78	0·2917
KRAS	0·67 (±0·08)	0·55 (±0·11)	0·49 (±0·25)	0·75 (±0·24)	0·47 (±0·09)	0·27 (±0·07)	0·72 (±0·03)	0·58 (±0·06)	0·33	0.00	0·3861
MECOM	0·36 (±0·17)	0·10 (±0·03)	0·88 (±0·10)	0·31 (±0·19)	0·18 (±0·04)	0·11 (±0·08)	0·71 (±0·03)	0·19 (±0·04)	0·08	0·88	0·8027
ALK	0·37 (±0·28)	0·10 (±0·03)	0·84 (±0·33)	0·33 (±0·33)	0·17 (±0·03)	0·12 (±0·06)	0·70 (±0·06)	0·25 (±0·10)	0·08	0·75	0·7380
CHD1	0·40 (±0·22)	0·08 (±0·03)	0·76 (±0·30)	0·38 (±0·25)	0·13 (±0·02)	0·08 (±0·06)	0·69 (±0·07)	0·15 (±0·03)	0·06	0·83	0·8215
TRPS1	0·55 (±0·17)	0·23 (±0·05)	0·60 (±0·23)	0·54 (±0·25)	0·31 (±0·03)	0·12 (±0·05)	0·66 (±0·03)	0·29 (±0·03)	0·17	0·56	0·9008
AKT1	0·19 (±0·18)	0·01 (±0·00)	1·00 (±0·00)	0·19 (±0·18)	0·02 (±0·01)	0·04 (±0·03)	0·65 (±0·27)	0·07 (±0·08)	0·01	1.00	-
PIK3CA	0·57 (±0·15)	0·23 (±0·11)	0·54 (±0·34)	0·58 (±0·27)	0·31 (±0·15)	0·10 (±0·12)	0·65 (±0·05)	0·37 (±0·08)	0·22	0·43	0·7644
APC	0·43 (±0·18)	0·78 (±0·07)	0·33 (±0·34)	0·74 (±0·31)	0·39 (±0·29)	0·07 (±0·10)	0·64 (±0·07)	0·82 (±0·04)	0·75	0·14	0·7638
CDK12	0·39 (±0·15)	0·03 (±0·01)	0·71 (±0·36)	0·38 (±0·16)	0·06 (±0·03)	0·03 (±0·11)	0·63 (±0·13)	0·16 (±0·10)	0·03	0·67	0·3362
SMAD2	0·29 (±0·13)	0·11 (±0·02)	0·91 (±0·19)	0·23 (±0·16)	0·20 (±0·03)	0·11 (±0·08)	0·63 (±0·09)	0·15 (±0·03)	0·09	0·40	0·0349
DUSP16	0·36	0·06	0·81	0·33	0·11	0·08	0·62	0·10	0·06	0·33	0·0248

	(±0·28)	(±0·03)	(±0·38)	(±0·32)	(±0·05)	(±0·07)	(±0·16)	(±0·03)			
TP53	0·52 (±0·05)	0·57 (±0·27)	0·20 (±0·18)	0·89 (±0·10)	0·27 (±0·22)	0·09 (±0·13)	0·61 (±0·02)	0·64 (±0·04)	0·53	0·14	0·3280
ATM	0·36 (±0·21)	0·09 (±0·02)	0·79 (±0·25)	0·32 (±0·25)	0·16 (±0·03)	0·08 (±0·06)	0·59 (±0·09)	0·13 (±0·03)	0·08	0·50	0·8098
SMG1	0·21 (±0·18)	0·08 (±0·02)	0·94 (±0·08)	0·16 (±0·19)	0·14 (±0·03)	0·02 (±0·18)	0·58 (±0·18)	0·13 (±0·08)	0·07	0·71	0·2335
ZHX2	0·26 (±0·19)	0·04 (±0·01)	0·75 (±0·20)	0·24 (±0·20)	0·07 (±0·01)	-0·00 (±0·06)	0·51 (±0·08)	0·08 (±0·03)	0·04	0·50	0·6480
NRAS	0·77 (±0·28)	0·02 (±0·02)	0·14 (±0·31)	0·80 (±0·32)	0·02 (±0·04)	-0·04 (±0·03)	0·48 (±0·13)	0·07 (±0·02)	0·06	0·17	0·3146
ELL2	0·46 (±0·32)	0·03 (±0·03)	0·50 (±0·38)	0·46 (±0·34)	0·06 (±0·05)	-0·03 (±0·12)	0·45 (±0·08)	0·05 (±0·01)	0·04	0·50	0·5055
CTNND1	0·54 (±0·30)	0·01 (±0·01)	0·36 (±0·38)	0·54 (±0·31)	0·02 (±0·02)	-0·03 (±0·04)	0·34 (±0·11)	0·02 (±0·00)	0·02	0.00	0·7825

Tab. S13: Association Rule Mining Results. Comprehensive overview of the relationships between various genetic alterations. Each relationship is characterized by an initiating genetic alteration, the 'Antecedent', and a resulting genetic alteration, the 'Consequent'. The relationships are evaluated based on several metrics. 'Support' refers to the frequency of occurrence of the relationship. 'Confidence' measures the predictive power of the relationship. 'Lift' assesses the degree of association between the Antecedent and Consequent. 'Leverage' calculates the difference between observed and expected Support. 'Conviction' gauges the dependability of the relationship. 'Zhang’s Metric' is a measure of the relationship's interestingness. The relationships are sorted in descending order of Confidence. The selection encompasses a variety of relevant and representative prediction targets from all prediction targets investigated in this study.

Antecedent	Consequent	Antecedent support	Consequent support	Support	Confidence	Lift	Leverage	Conviction	Zhang's metric
BMPR2	MSI	0·15	0·25	0·15	0·95	3·88	0·11	16·07	0·88
WNT16	hypermutated	0·03	0·26	0·03	0·95	3·69	0·02	15·58	0·75
WNT16	MSI	0·03	0·25	0·03	0·95	3·88	0·02	15·84	0·77
MSI	hypermutated	0·25	0·26	0·23	0·94	3·66	0·17	13·4	0·96
BMPR2	hypermutated	0·15	0·26	0·14	0·94	3·64	0·1	12·16	0·86
NRAS	MSS	0·04	0·75	0·04	0·93	1·24	0·01	3·75	0·2
RFX5	MSI	0·03	0·25	0·03	0·93	3·8	0·02	11·31	0·76
KRAS	MSS	0·33	0·75	0·3	0·93	1·23	0·06	3·32	0·28
ZNRF3	hypermutated	0·11	0·26	0·1	0·92	3·58	0·07	9·52	0·81
ELL2	hypermutated	0·02	0·26	0·02	0·92	3·55	0·01	8·91	0·73
CDK12	hypermutated	0·04	0·26	0·03	0·91	3·51	0·02	7·87	0·74
hypermutated	MSI	0·26	0·25	0·23	0·9	3·66	0·17	7·54	0·98
MECOM	hypermutated	0·06	0·26	0·05	0·9	3·49	0·04	7·42	0·76
PLEKHA6	MSI	0·06	0·25	0·06	0·9	3·65	0·04	7·38	0·78
CCDC40	hypermutated	0·04	0·26	0·04	0·89	3·45	0·02	6·8	0·74
ZNRF3	MSI	0·11	0·25	0·1	0·89	3·62	0·07	6·83	0·81
RFX5	hypermutated	0·03	0·26	0·03	0·89	3·45	0·02	6·68	0·73
FHOD3	hypermutated	0·08	0·26	0·07	0·89	3·44	0·05	6·56	0·77
PLEKHA6	hypermutated	0·06	0·26	0·06	0·89	3·44	0·04	6·53	0·76
FHOD3	MSI	0·08	0·25	0·07	0·88	3·57	0·05	6·2	0·78
CTNND1	hypermutated	0·04	0·26	0·04	0·88	3·4	0·03	6·04	0·74
MECOM	MSI	0·06	0·25	0·05	0·87	3·56	0·04	6·03	0·76
TP53	MSS	0·58	0·75	0·5	0·87	1·16	0·07	1·96	0·32
CHD1	hypermutated	0·04	0·26	0·04	0·87	3·36	0·03	5·57	0·73
APC	MSS	0·59	0·75	0·51	0·86	1·14	0·06	1·76	0·3
CTNND1	MSI	0·04	0·25	0·04	0·86	3·5	0·03	5·37	0·74
RNF43	MSI	0·21	0·25	0·17	0·84	3·41	0·12	4·67	0·89
CHD1	MSI	0·04	0·25	0·04	0·83	3·39	0·03	4·53	0·74
ELL2	MSI	0·02	0·25	0·01	0·83	3·39	0·01	4·53	0·72
CDK12	MSI	0·04	0·25	0·03	0·81	3·3	0·02	4	0·72
RNF43	hypermutated	0·21	0·26	0·17	0·81	3·13	0·11	3·86	0·86
ZHX2	hypermutated	0·03	0·26	0·03	0·8	3·12	0·02	3·79	0·7
RFX5	BRAF	0·03	0·23	0·03	0·8	3·49	0·02	3·85	0·74
AKT1	hypermutated	0·04	0·26	0·03	0·79	3·07	0·02	3·58	0·7
ELL2	BRAF	0·02	0·23	0·01	0·79	3·45	0·01	3·7	0·72
TGFBR2	hypermutated	0·06	0·26	0·05	0·78	3·04	0·03	3·42	0·71
CCDC40	MSI	0·04	0·25	0·03	0·78	3·18	0·02	3·46	0·71
SMG1	hypermutated	0·04	0·26	0·03	0·78	3·02	0·02	3·37	0·7
MECOM	BRAF	0·06	0·23	0·04	0·78	3·38	0·03	3·43	0·75
TBX3	hypermutated	0·05	0·26	0·04	0·77	2·98	0·03	3·2	0·7
ZNRF3	RNF43	0·11	0·21	0·08	0·77	3·68	0·06	3·39	0·82
CCDC40	RNF43	0·04	0·21	0·03	0·76	3·66	0·02	3·35	0·76
NRAS	APC	0·04	0·59	0·03	0·75	1·28	0·01	1·67	0·23
ALK	hypermutated	0·08	0·26	0·06	0·75	2·9	0·04	2·94	0·71
TGFBR2	MSI	0·06	0·25	0·04	0·75	3·04	0·03	2·98	0·71
FHOD3	BRAF	0·08	0·23	0·06	0·74	3·22	0·04	2·95	0·75
PLEKHA6	BRAF	0·06	0·23	0·05	0·74	3·22	0·03	2·95	0·74
CDK12	RNF43	0·04	0·21	0·03	0·74	3·53	0·02	3	0·75
CHD1	RNF43	0·04	0·21	0·03	0·73	3·52	0·02	2·97	0·75
CHD1	BRAF	0·04	0·23	0·03	0·73	3·2	0·02	2·89	0·72
KRAS	APC	0·33	0·59	0·24	0·73	1·24	0·05	1·52	0·29
KIF1A	hypermutated	0·1	0·26	0·08	0·73	2·82	0·05	2·71	0·72
FHOD3	RNF43	0·08	0·21	0·06	0·72	3·46	0·04	2·84	0·78
WNT16	BRAF	0·03	0·23	0·02	0·71	3·12	0·01	2·7	0·7
WNT16	RNF43	0·03	0·21	0·02	0·71	3·43	0·02	2·77	0·73
MSI	RNF43	0·25	0·21	0·17	0·71	3·41	0·12	2·74	0·94
BMPR2	RNF43	0·15	0·21	0·11	0·7	3·38	0·08	2·68	0·83
MECOM	RNF43	0·06	0·21	0·04	0·7	3·36	0·03	2·64	0·74
ZHX2	MSI	0·03	0·25	0·02	0·7	2·83	0·01	2·48	0·67
TBX3	MSI	0·05	0·25	0·03	0·7	2·83	0·02	2·48	0·68
SMG1	MSI	0·04	0·25	0·03	0·69	2·83	0·02	2·47	0·67
ACVR1B	hypermutated	0·05	0·26	0·04	0·69	2·69	0·02	2·42	0·66
RFX5	RNF43	0·03	0·21	0·02	0·69	3·3	0·02	2·54	0·72
BRAF	hypermutated	0·23	0·26	0·16	0·68	2·65	0·1	2·35	0·81
BRAF	MSI	0·23	0·25	0·16	0·68	2·76	0·1	2·34	0·83
DUSP16	hypermutated	0·03	0·26	0·02	0·68	2·62	0·01	2·29	0·64

ALK	MSI	0·08	0·25	0·05	0·67	2·74	0·03	2·31	0·69
CCDC40	BRAF	0·04	0·23	0·03	0·67	2·93	0·02	2·36	0·69
MSS	APC	0·75	0·59	0·51	0·67	1·14	0·06	1·25	0·5
RNF43	BRAF	0·21	0·23	0·14	0·67	2·92	0·09	2·34	0·83
MSS	TP53	0·75	0·58	0·5	0·67	1·16	0·07	1·28	0·56
TRPS1	hypermutated	0·06	0·26	0·04	0·67	2·59	0·02	2·23	0·65
ELL2	RNF43	0·02	0·21	0·01	0·67	3·2	0·01	2·37	0·7
NRAS	TP53	0·04	0·58	0·03	0·66	1·14	0	1·23	0·13
hypermutated	RNF43	0·26	0·21	0·17	0·65	3·13	0·11	2·28	0·92
TGFBR2	BRAF	0·06	0·23	0·04	0·65	2·84	0·03	2·21	0·69
DUSP16	BRAF	0·03	0·23	0·02	0·65	2·83	0·01	2·19	0·66
DUSP16	MSI	0·03	0·25	0·02	0·65	2·64	0·01	2·15	0·64
TP53	APC	0·58	0·59	0·37	0·65	1·1	0·03	1·17	0·22
WNT16	BMPR2	0·03	0·15	0·02	0·64	4·21	0·01	2·37	0·79
BMPR2	BRAF	0·15	0·23	0·1	0·64	2·79	0·06	2·13	0·76
PLEKHA6	RNF43	0·06	0·21	0·04	0·64	3·05	0·03	2·18	0·72
APC	TP53	0·59	0·58	0·37	0·64	1·1	0·03	1·16	0·22
MSI	BRAF	0·25	0·23	0·16	0·63	2·76	0·1	2·1	0·85
CTNND1	BRAF	0·04	0·23	0·03	0·63	2·76	0·02	2·09	0·66
SMAD2	APC	0·04	0·59	0·03	0·63	1·07	0	1·11	0·07
TRPS1	MSI	0·06	0·25	0·04	0·63	2·56	0·02	2·03	0·64
ZNRF3	BRAF	0·11	0·23	0·07	0·62	2·72	0·04	2·05	0·71
CDK12	BRAF	0·04	0·23	0·02	0·62	2·72	0·01	2·04	0·66
CHD1	BMPR2	0·04	0·15	0·03	0·62	4·04	0·02	2·21	0·79
KIF1A	MSI	0·1	0·25	0·06	0·62	2·51	0·04	1·97	0·67
PIK3CA	APC	0·18	0·59	0·11	0·62	1·05	0·01	1·07	0·05
ATM	hypermutated	0·11	0·26	0·07	0·61	2·38	0·04	1·92	0·65
ACVR1B	MSI	0·05	0·25	0·03	0·61	2·5	0·02	1·95	0·63
BRAF	RNF43	0·23	0·21	0·14	0·61	2·92	0·09	2·03	0·85
ZHX2	RNF43	0·03	0·21	0·02	0·61	2·92	0·01	2·02	0·68
hypermutated	BRAF	0·26	0·23	0·16	0·61	2·65	0·1	1·97	0·84
ZNRF3	BMPR2	0·11	0·15	0·07	0·6	3·96	0·05	2·14	0·84
AKT1	MSI	0·04	0·25	0·02	0·6	2·46	0·01	1·9	0·62
RFX5	BMPR2	0·03	0·15	0·02	0·6	3·93	0·01	2·12	0·77
CTNND1	RNF43	0·04	0·21	0·02	0·6	2·86	0·02	1·96	0·68
DUSP16	APC	0·03	0·59	0·02	0·59	1·01	0	1·01	0·01
DUSP16	RNF43	0·03	0·21	0·02	0·59	2·85	0·01	1·95	0·67
SMG1	BRAF	0·04	0·23	0·03	0·59	2·59	0·02	1·89	0·64
MSI	BMPR2	0·25	0·15	0·15	0·59	3·88	0·11	2·08	0·98
PIK3CA	MSS	0·18	0·75	0·11	0·58	0·77	-0·03	0·58	-0·27
TRPS1	BRAF	0·06	0·23	0·03	0·58	2·52	0·02	1·82	0·64
SMG1	RNF43	0·04	0·21	0·02	0·58	2·76	0·02	1·87	0·67
ATM	APC	0·11	0·59	0·06	0·58	0·98	0	0·97	-0·03
ATM	MSI	0·11	0·25	0·06	0·57	2·31	0·04	1·75	0·64
PLEKHA6	BMPR2	0·06	0·15	0·04	0·57	3·72	0·03	1·96	0·78
ZHX2	BRAF	0·03	0·23	0·02	0·57	2·47	0·01	1·77	0·61
SMAD2	MSS	0·04	0·75	0·03	0·56	0·75	-0·01	0·56	-0·26
ALK	RNF43	0·08	0·21	0·04	0·56	2·69	0·03	1·8	0·68
KRAS	TP53	0·33	0·58	0·18	0·56	0·96	-0·01	0·95	-0·05
hypermutated	BMPR2	0·26	0·15	0·14	0·56	3·64	0·1	1·91	0·98
TGFBR2	RNF43	0·06	0·21	0·03	0·55	2·66	0·02	1·78	0·66
TBX3	RNF43	0·05	0·21	0·03	0·55	2·64	0·02	1·76	0·65
MECOM	BMPR2	0·06	0·15	0·03	0·55	3·6	0·02	1·88	0·77
SMAD2	hypermutated	0·04	0·26	0·02	0·55	2·13	0·01	1·64	0·55
CCDC40	APC	0·04	0·59	0·02	0·55	0·93	0	0·9	-0·08
FHOD3	BMPR2	0·08	0·15	0·04	0·54	3·53	0·03	1·84	0·78
MECOM	ZNRF3	0·06	0·11	0·03	0·54	4·87	0·02	1·92	0·84
ACVR1B	APC	0·05	0·59	0·03	0·53	0·91	0	0·88	-0·1
RFX5	ZNRF3	0·03	0·11	0·02	0·53	4·83	0·01	1·91	0·82
RFX5	FHOD3	0·03	0·08	0·02	0·53	6·47	0·01	1·97	0·87
AKT1	APC	0·04	0·59	0·02	0·53	0·9	0	0·87	-0·11
CTNND1	BMPR2	0·04	0·15	0·02	0·53	3·45	0·02	1·79	0·74
SMG1	APC	0·04	0·59	0·02	0·53	0·89	0	0·87	-0·11
WNT16	FHOD3	0·03	0·08	0·02	0·52	6·36	0·01	1·93	0·87
CHD1	FHOD3	0·04	0·08	0·02	0·52	6·27	0·02	1·9	0·88
RNF43	BMPR2	0·21	0·15	0·11	0·52	3·38	0·08	1·75	0·89
CDK12	TP53	0·04	0·58	0·02	0·51	0·88	0	0·86	-0·12
CCDC40	BMPR2	0·04	0·15	0·02	0·51	3·34	0·01	1·73	0·73
TBX3	BMPR2	0·05	0·15	0·03	0·51	3·32	0·02	1·72	0·74
KIF1A	RNF43	0·1	0·21	0·05	0·51	2·43	0·03	1·61	0·66
TRPS1	RNF43	0·06	0·21	0·03	0·5	2·4	0·02	1·58	0·62
MECOM	FHOD3	0·06	0·08	0·03	0·5	6·07	0·02	1·84	0·89
CHD1	ZNRF3	0·04	0·11	0·02	0·5	4·53	0·02	1·78	0·81
ZHX2	APC	0·03	0·59	0·02	0·5	0·85	0	0·82	-0·16

Tab. S14: Performance metrics of Multi-Target Transformers for external validation on the GECCO test cohort. Mean and standard deviation from the 7 folds of the cross-validation for relevant selected prediction targets. The threshold for binary classification is pre-defined as 0·5. The evaluation metrics include the Matthews Correlation Coefficient (MCC), the Area Under the Receiver Operating Characteristic Curve (AUROC), and the Area Under the Precision-Recall Curve (AUPRC), along with the corresponding mutation rates in external cohorts. Mutation rate contains the ratio of mutations of the respective target within the external data set. The data is sorted for AUROC, as shown in Fig. S3B.

**Target**	**Accuracy**	**Precision**	**Sensitivity**	**Specificity**	**F1 Score**	**MCC**	**AUROC**	**AUPRC**	**Mutation Rate**	**(Target MUT + MSI) / Target MUT**
MSI	0·85 (±0·04)	0·68 (±0·09)	0·88 (±0·05)	0·83 (±0·07)	0·76 (±0·04)	0·67 (±0·06)	0·93 (±0·01)	0·86 (±0·03)	0·27	1·00
PLEKHA6	0·77 (±0·05)	0·28 (±0·04)	0·91 (±0·08)	0·76 (±0·06)	0·42 (±0·04)	0·42 (±0·03)	0·90 (±0·01)	0·38 (±0·06)	0·04	0·97
hypermutated	0·78 (±0·05)	0·58 (±0·07)	0·85 (±0·04)	0·76 (±0·08)	0·69 (±0·04)	0·56 (±0·05)	0·88 (±0·01)	0·74 (±0·02)	0·28	0·92
CHD1	0·76 (±0·07)	0·12 (±0·03)	0·84 (±0·15)	0·76 (±0·08)	0·2 (±0·03)	0·26 (±0·02)	0·87 (±0·02)	0·17 (±0·06)	0·02	0·91
BMPR2	0·75 (±0·05)	0·4 (±0·05)	0·89 (±0·08)	0·73 (±0·07)	0·55 (±0·04)	0·48 (±0·03)	0·87 (±0·01)	0·45 (±0·02)	0·16	0·97
RNF43	0·77 (±0·04)	0·5 (±0·05)	0·82 (±0·06)	0·76 (±0·06)	0·62 (±0·02)	0·5 (±0·03)	0·86 (±0·01)	0·59 (±0·02)	0·22	0·86
FHOD3	0·79 (±0·06)	0·33 (±0·06)	0·77 (±0·14)	0·79 (±0·08)	0·45 (±0·04)	0·4 (±0·04)	0·84 (±0·02)	0·38 (±0·03)	0·06	0·81
WNT16	0·77 (±0·08)	0·14 (±0·04)	0·76 (±0·14)	0·77 (±0·09)	0·24 (±0·05)	0·26 (±0·04)	0·82 (±0·02)	0·18 (±0·02)	0·02	0·93
ELL2	0·71 (±0·08)	0·03 (±0·0)	0·75 (±0·14)	0·71 (±0·09)	0·06 (±0·01)	0·11 (±0·01)	0·79 (±0·05)	0·06 (±0·01)	0·01	0·75
MECOM	0·75 (±0·05)	0·20 (±0·04)	0·70 (±0·09)	0·75 (±0·06)	0·31 (±0·05)	0·28 (±0·07)	0·78 (±0·04)	0·21 (±0·04)	0·04	0·88
BRAF	0·73 (±0·03)	0·42 (±0·03)	0·73 (±0·08)	0·73 (±0·05)	0·53 (±0·01)	0·39 (±0·01)	0·78 (±0·01)	0·45 (±0·02)	0·21	0·71
CDK12	0·60 (±0·07)	0·09 (±0·01)	0·86 (±0·07)	0·58 (±0·08)	0·16 (±0·01)	0·19 (±0·01)	0·78 (±0·02)	0·13 (±0·02)	0·04	0·86
TGFBR2	0·64 (±0·07)	0·14 (±0·01)	0·81 (±0·11)	0·63 (±0·08)	0·24 (±0·02)	0·23 (±0·02)	0·77 (±0·02)	0·17 (±0·01)	0·07	0·73
RFX5	0·71 (±0·09)	0·11 (±0·02)	0·77 (±0·11)	0·71 (±0·1)	0·18 (±0·03)	0·21 (±0·04)	0·77 (±0·02)	0·12 (±0·01)	0·02	0·85
CCDC40	0·63 (±0·1)	0·07 (±0·01)	0·76 (±0·22)	0·62 (±0·12)	0·13 (±0·01)	0·15 (±0·04)	0·76 (±0·04)	0·1 (±0·03)	0·04	0·83
SMG1	0·58 (±0·13)	0·08 (±0·02)	0·82 (±0·16)	0·57 (±0·14)	0·14 (±0·04)	0·16 (±0·06)	0·76 (±0·07)	0·1 (±0·02)	0·04	0·77

ACVR1B	0·61 (±0·08)	0·11 (±0·01)	0·78 (±0·11)	0·60 (±0·09)	0·2 (±0·02)	0·18 (±0·03)	0·75 (±0·03)	0·17 (±0·01)	0·06	0·51
ZNRF3	0·70 (±0·04)	0·23 (±0·02)	0·73 (±0·05)	0·69 (±0·05)	0·34 (±0·02)	0·28 (±0·02)	0·75 (±0·01)	0·25 (±0·01)	0·11	0·87
CTNND1	0·67 (±0·07)	0·09 (±0·02)	0·74 (±0·06)	0·66 (±0·07)	0·17 (±0·03)	0·18 (±0·04)	0·75 (±0·03)	0·1 (±0·01)	0·04	0·89
ZHX2	0·6 (±0·06)	0·07 (±0·0)	0·81 (±0·13)	0·59 (±0·06)	0·12 (±0·01)	0·15 (±0·03)	0·74 (±0·02)	0·08 (±0·01)	0·03	0·77
TBX3	0·53 (±0·13)	0·1 (±0·02)	0·83 (±0·11)	0·51 (±0·14)	0·17 (±0·02)	0·16 (±0·03)	0·74 (±0·03)	0·14 (±0·03)	0·06	0·76
DUSP16	0·46 (±0·18)	0·04 (±0·01)	0·83 (±0·11)	0·45 (±0·18)	0·07 (±0·02)	0·09 (±0·04)	0·74 (±0·04)	0·08 (±0·03)	0·02	0·75
TRPS1	0·48 (±0·17)	0·14 (±0·05)	0·8 (±0·07)	0·45 (±0·2)	0·23 (±0·07)	0·15 (±0·09)	0·73 (±0·03)	0·24 (±0·05)	0·04	0·68
TP53	0·65 (±0·01)	0·7 (±0·05)	0·61 (±0·15)	0·68 (±0·17)	0·64 (±0·05)	0·31 (±0·03)	0·72 (±0·02)	0·72 (±0·03)	0·53	0·13
AKT1	0·48 (±0·17)	0·07 (±0·02)	0·81 (±0·12)	0·47 (±0·18)	0·13 (±0·03)	0·12 (±0·04)	0·7 (±0·03)	0·1 (±0·02)	0·05	0·63
ALK	0·43 (±0·23)	0·12 (±0·03)	0·84 (±0·14)	0·39 (±0·26)	0·21 (±0·04)	0·14 (±0·06)	0·7 (±0·02)	0·17 (±0·02)	0·08	0·77
KIF1A	0·46 (±0·19)	0·14 (±0·02)	0·8 (±0·14)	0·42 (±0·23)	0·23 (±0·03)	0·14 (±0·05)	0·67 (±0·03)	0·19 (±0·03)	0·10	0·63
APC	0·63 (±0·06)	0·7 (±0·05)	0·68 (±0·23)	0·55 (±0·2)	0·66 (±0·15)	0·25 (±0·04)	0·66 (±0·02)	0·71 (±0·04)	0·59	0·15
SMAD2	0·41 (±0·16)	0·07 (±0·01)	0·8 (±0·11)	0·39 (±0·17)	0·13 (±0·02)	0·09 (±0·03)	0·65 (±0·03)	0·11 (±0·03)	0·05	0·39
KRAS	0·61 (±0·04)	0·44 (±0·03)	0·57 (±0·19)	0·63 (±0·15)	0·48 (±0·07)	0·19 (±0·04)	0·65 (±0·03)	0·46 (±0·03)	0·33	0·08
ATM	0·61 (±0·08)	0·14 (±0·02)	0·62 (±0·07)	0·61 (±0·1)	0·23 (±0·02)	0·14 (±0·03)	0·63 (±0·01)	0·15 (±0·01)	0·09	0·63
PIK3CA	0·49 (±0·09)	0·19 (±0·02)	0·66 (±0·14)	0·46 (±0·14)	0·29 (±0·02)	0·09 (±0·04)	0·56 (±0·03)	0·19 (±0·02)	0·16	0·42
NRAS	0·58 (±0·14)	0·05 (±0·01)	0·49 (±0·21)	0·59 (±0·15)	0·09 (±0·02)	0·03 (±0·03)	0·56 (±0·04)	0·06 (±0·01)	0·04	0·07

Tab. S15: Comparison of the Microsatellite Instability (MSI) prediction scores with the target prediction scores within subgroups. The data is organized by subgroups (Fig. 1D), with each subgroup representing a specific category such as MSI, Microsatellite Stability (MSS), Prediction Target Wild Type (WT), and Prediction Target Mutated (MUT). For each subgroup, the table provides the count of samples (N) and the median values for the prediction scores for both the train set (internal, i) and test set (external, e). The p-value from the Mann-Whitney U test is also included, which indicates the statistical significance of the comparison between the MSI and prediction target scores within each subgroup. This data is visualized within Fig. 4 and Figs. S4-S5.

Target	i/e	MSS/WT_N	MSS/WT_p	MSS/WT_MSI_median	MSS/WT_target_median	MSI/WT_N	MSI/WT_p	MSI/WT_MSI_median	MSI/WT_target_median	MSS/MUT_N	MSS/MUT_p	MSS/MUT_MSI_median	MSS/MUT_target_median	MSI/MUT_N	MSI/MUT_p	MSI/MUT_MSI_median	MSI/MUT_target_median
ACVR1B	i	563	6·01e-40	0·01	0·08	133	1·04e-7	0·95	0·46	10	4·92e-1	0·02	0·13	25	2·26e-3	0·92	0·60
	e	449	1·30e-63	0·12	0·29	157	3·06e-13	0·89	0·78	19	2·93e-1	0·69	0·62	20	1·99e-3	0·93	0·81
AKT1	i	563	2·37e-39	0·01	0·10	146	1·24e-8	0·91	0·36	10	1·37e-2	0·00	0·51	12	1·61e-2	0·99	0·50
	e	457	5·90e-66	0·12	0·44	158	3·74e-15	0·89	0·67	11	9·77e-4	0·12	0·53	19	1·69e-3	0·93	0·69
ALK	i	551	2·76e-48	0·01	0·19	128	2·11e-8	0·95	0·47	22	4·28e-4	0·00	0·22	30	6·85e-1	0·77	0·56
	e	456	3·03e-72	0·12	0·49	136	2·35e-10	0·90	0·76	12	2·44e-3	0·25	0·53	41	1·66e-2	0·83	0·74
APC	i	196	2·80e-22	0·01	0·54	103	1·09e-9	0·95	0·21	377	1·59e-43	0·01	0·66	55	3·70e-2	0·94	0·31
	e	146	7·75e-17	0·14	0·62	118	8·35e-19	0·91	0·33	322	4·96e-43	0·11	0·66	59	1·10e-8	0·88	0·39
ATM	i	530	2·57e-45	0·01	0·16	111	1·60e-4	0·89	0·53	43	1·83e-4	0·02	0·20	47	1·60e-3	0·98	0·54
	e	446	7·23e-62	0·12	0·37	140	1·18e-17	0·90	0·72	22	2·62e-5	0·09	0·32	37	4·55e-6	0·89	0·72
BMPR2	i	566	2·16e-8	0·01	0·02	60	1·55e-5	0·74	0·40	7	9·38e-1	0·01	0·04	98	6·51e-6	0·97	0·83
	e	465	2·60e-36	0·12	0·16	74	2·61e-5	0·92	0·88	3	5·00e-1	0·54	0·58	103	9·24e-9	0·89	0·82
BRAF	i	510	2·30e-37	0·00	0·06	41	2·91e-1	0·53	0·72	63	5·89e-8	0·03	0·44	117	2·05e-1	0·97	0·91
	e	429	5·02e-49	0·11	0·22	81	1·22e-7	0·90	0·85	39	5·59e-4	0·23	0·34	96	9·23e-11	0·89	0·83
CCDC40	i	565	8·85e-38	0·01	0·06	136	2·03e-9	0·94	0·49	8	2·50e-1	0·01	0·38	22	1·09e-3	0·98	0·53
	e	464	4·18e-56	0·12	0·33	158	7·49e-21	0·90	0·70	4	1·25e-1	0·34	0·52	19	6·63e-2	0·83	0·66
CDK12	i	567	6·88e-34	0·01	0·07	141	9·54e-12	0·95	0·45	6	3·13e-1	0·01	0·26	17	5·05e-2	0·90	0·51
	e	464	5·30e-68	0·12	0·36	152	8·54e-18	0·89	0·74	4	1·25e-1	0·39	0·53	25	2·03e-3	0·90	0·72
CHD1	i	417	1·46e-19	0·01	0·07	108	9·83e-7	0·94	0·49	9	1·00e0	0·13	0·23	39	1·58e-4	0·99	0·68
	e	255	5·36e-16	0·13	0·20	55	6·27e-10	0·86	0·65	1	1·00e0	0·30	0·31	10	1·95e-3	0·87	0·67
CTNND1	i	568	3·09e-27	0·01	0·05	134	6·32e-12	0·94	0·42	5	3·13e-1	0·23	0·03	24	3·66e-5	0·97	0·42
	e	465	9·42e-44	0·12	0·27	152	8·00e-24	0·90	0·74	3	1·00e0	0·38	0·38	25	1·49e-6	0·88	0·72
DUSP16	i	564	2·71e-34	0·01	0·12	147	5·14e-12	0·94	0·47	9	3·91e-3	0·00	0·17	11	1·86e-2	0·99	0·38
	e	464	7·34e-72	0·12	0·47	165	2·66e-16	0·89	0·73	4	1·25e-1	0·21	0·51	12	9·77e-4	0·91	0·74
ELL2	i	423	8·96e-14	0·01	0·07	131	9·37e-12	0·96	0·53	3	7·50e-1	0·03	0·39	16	7·63e-3	0·99	0·34
	e	255	9·70e-26	0·13	0·27	62	1·13e-8	0·86	0·68	1	1·00e0	0·30	0·28	3	2·50e-1	0·86	0·74
FHOD3	i	420	3·87e-15	0·01	0·05	79	1·36e-8	0·95	0·54	6	1·00e0	0·42	0·15	68	1·21e-5	0·97	0·65
	e	249	2·34e-18	0·13	0·19	36	3·11e-8	0·84	0·73	7	1·00e0	0·30	0·39	29	3·73e-9	0·87	0·75
hypermutated	i	551	1·73e-28	0·01	0·02	5	3·13e-1	1·00	0·98	22	2·38e-6	0·00	0·04	153	2·66e-2	0·94	0·96
	e	454	3·14e-68	0·12	0·22	13	1·71e-3	0·96	0·93	14	1·22e-4	0·10	0·16	164	8·27e-1	0·89	0·88
KIF1A	i	540	8·89e-41	0·01	0·14	111	1·25e-4	0·94	0·50	33	5·63e-6	0·00	0·31	47	8·50e-3	0·97	0·48
	e	445	3·38e-70	0·12	0·45	137	1·63e-10	0·90	0·77	23	1·67e-5	0·25	0·56	40	5·54e-1	0·84	0·78
KRAS	i	349	3·12e-34	0·00	0·35	142	6·33e-12	0·97	0·10	224	5·54e-25	0·01	0·51	16	7·82e-1	0·16	0·50
	e	271	3·56e-30	0·11	0·51	160	5·78e-25	0·89	0·32	197	8·63e-22	0·13	0·54	17	4·58e-5	0·91	0·36
MECOM	i	420	1·52e-10	0·01	0·04	103	1·96e-9	0·95	0·58	6	1·00e0	0·07	0·08	44	2·01e-5	0·97	0·64
	e	253	3·81e-	0·13	0·22	42	3·95e-9	0·87	0·74	3	2·50e-1	0·17	0·24	23	2·15e-4	0·82	0·70

			27														
NRAS	i	542	1·83e-35	0·01	0·20	156	1·30e-16	0·94	0·12	31	5·41e-4	0·00	0·23	2	1·00e0	0·50	0·03
	e	442	1·96e-44	0·12	0·50	175	4·27e-28	0·90	0·34	26	8·35e-4	0·12	0·49	2	1·00e0	0·30	0·51
PIK3CA	i	486	1·18e-32	0·01	0·09	96	1·09e-4	0·93	0·45	87	1·68e-7	0·01	0·16	62	2·89e-5	0·97	0·48
	e	407	1·82e-59	0·12	0·45	133	8·83e-17	0·91	0·71	61	4·85e-10	0·12	0·53	44	4·81e-5	0·83	0·66
PLEKHA6	i	418	7·19e-19	0·01	0·07	97	1·85e-8	0·94	0·54	8	7·81e-3	0·03	0·19	50	2·00e-7	0·99	0·65
	e	255	1·74e-33	0·13	0·24	37	1·63e-4	0·82	0·72	1	1·00e0	0·95	0·83	28	1·49e-8	0·89	0·75
RFX5	i	425	1·49e-8	0·01	0·03	120	1·93e-12	0·95	0·50	1	1·00e0	0·13	0·01	27	2·52e-6	0·99	0·71
	e	254	1·84e-31	0·13	0·24	54	9·01e-7	0·86	0·74	2	5·00e-1	0·07	0·16	11	9·77e-4	0·86	0·72
RNF43	i	546	1·16e-28	0·01	0·03	45	2·62e-1	0·79	0·69	27	1·51e-2	0·02	0·16	113	1·04e-2	0·97	0·86
	e	448	2·19e-41	0·12	0·17	54	2·53e-6	0·86	0·83	20	4·41e-2	0·29	0·36	123	1·74e-13	0·90	0·83
SMAD2	i	558	1·13e-53	0·01	0·17	145	1·90e-11	0·97	0·47	15	4·21e-1	0·04	0·37	13	5·42e-1	0·04	0·14
	e	448	7·36e-72	0·12	0·48	164	3·06e-7	0·89	0·77	20	1·34e-4	0·34	0·65	13	4·64e-3	0·90	0·78
SMG1	i	561	3·41e-39	0·01	0·11	138	4·95e-11	0·95	0·51	12	3·01e-1	0·01	0·16	20	8·26e-2	0·41	0·35
	e	462	3·20e-65	0·12	0·38	157	7·87e-19	0·89	0·69	6	3·13e-1	0·43	0·57	20	2·67e-5	0·92	0·69
TBX3	i	561	9·45e-48	0·01	0·11	139	4·82e-9	0·95	0·42	12	6·77e-1	0·03	0·23	19	9·45e-3	0·89	0·24
	e	459	1·69e-69	0·12	0·43	149	5·45e-10	0·89	0·74	9	1·95e-2	0·37	0·55	28	7·20e-5	0·92	0·74
TGFBR2	i	564	2·92e-44	0·01	0·07	128	9·85e-10	0·95	0·50	9	3·91e-3	0·01	0·27	30	5·01e-3	0·91	0·54
	e	456	7·01e-62	0·12	0·27	145	2·79e-14	0·89	0·77	12	3·42e-3	0·40	0·52	32	5·43e-5	0·90	0·76
TP53	i	174	1·01e-16	0·01	0·50	105	1·23e-5	0·89	0·21	399	4·77e-56	0·00	0·76	53	2·48e-2	0·97	0·43
	e	170	1·54e-8	0·16	0·48	133	6·73e-22	0·90	0·27	298	5·23e-41	0·10	0·58	44	2·80e-9	0·89	0·31
TRPS1	i	406	2·38e-38	0·01	0·22	118	1·82e-7	0·97	0·52	20	2·96e-2	0·02	0·38	29	6·23e-2	0·78	0·47
	e	247	3·52e-40	0·13	0·49	46	6·84e-3	0·82	0·71	9	7·81e-3	0·12	0·54	19	9·54e-5	0·90	0·73
WNT16	i	425	3·11e-4	0·01	0·03	121	2·75e-13	0·95	0·49	1	1·00e0	0·27	0·12	26	5·66e-7	0·97	0·48
	e	255	9·02e-12	0·13	0·19	52	1·39e-9	0·86	0·68	1	1·00e0	0·02	0·08	13	2·44e-4	0·87	0·67
ZHX2	i	564	2·09e-32	0·01	0·07	143	4·03e-12	0·95	0·41	9	4·26e-1	0·00	0·17	15	2·29e-1	0·40	0·43
	e	463	1·18e-53	0·12	0·33	160	2·43e-19	0·89	0·69	5	6·25e-1	0·48	0·55	17	6·56e-4	0·92	0·68
ZNRF3	i	565	3·83e-26	0·01	0·05	87	2·47e-4	0·87	0·52	8	3·83e-1	0·01	0·03	71	6·68e-6	0·99	0·65
	e	459	1·66e-43	0·12	0·21	116	3·27e-16	0·90	0·80	9	3·91e-3	0·07	0·17	61	4·04e-10	0·89	0·76

Tab. S16: Comparison of microsatellite instability (MSI) scores and prediction target scores among subgroups. The results are arranged by subgroups (Fig. 1D), where each subgroup represents a particular combination of microsatellite and prediction target mutation status, such as MSI, Microsatellite Stability (MSS), Prediction Target Wild Type (WT) and Prediction Target Mutated (MUT). For each subgroup within the train and test dataset, the table provides the p-value obtained from the Wilcoxon test for comparing MSI and prediction target scores, respectively, which is shown in the second column. These results are illustrated in Fig. 4 and Figs. S4-S5.

		Train set		Test set	
**Target**	**comparison**	**p_value_MSI**	**p_value_target**	**p_value_MSI**	**p_value_target**
ACVR1B	MSS/WT vs MSI/WT	0·00e0	0·00e0	0·00e0	0·00e0
	MSS/WT vs MSS/MUT	1·05e-1	1·25e-1	5·00e-5	1·00e-5
	MSS/WT vs MSI/MUT	0·00e0	0·00e0	0·00e0	0·00e0
	MSI/WT vs MSS/MUT	9·10e-3	7·69e-2	9·00e-5	5·60e-4
	MSI/WT vs MSI/MUT	6·00e-1	8·19e-1	1·29e-1	1·52e-1
	MSS/MUT vs MSI/MUT	1·85e-2	1·21e-1	4·20e-4	8·70e-4
AKT1	MSS/WT vs MSI/WT	0·00e0	0·00e0	0·00e0	0·00e0
	MSS/WT vs MSS/MUT	5·51e-1	7·50e-3	3·86e-1	1·94e-2
	MSS/WT vs MSI/MUT	0·00e0	7·00e-5	0·00e0	0·00e0
	MSI/WT vs MSS/MUT	1·60e-4	7·92e-1	0·00e0	4·80e-4
	MSI/WT vs MSI/MUT	1·61e-1	3·17e-1	8·48e-1	3·49e-1
	MSS/MUT vs MSI/MUT	5·40e-4	1·00e0	5·00e-5	1·45e-3
ALK	MSS/WT vs MSI/WT	0·00e0	0·00e0	0·00e0	0·00e0
	MSS/WT vs MSS/MUT	7·79e-1	3·19e-1	1·31e-1	2·62e-1
	MSS/WT vs MSI/MUT	0·00e0	0·00e0	0·00e0	0·00e0
	MSI/WT vs MSS/MUT	0·00e0	8·22e-3	0·00e0	0·00e0
	MSI/WT vs MSI/MUT	3·31e-1	5·51e-1	2·67e-2	7·31e-2
	MSS/MUT vs MSI/MUT	6·00e-5	7·44e-3	1·00e-5	2·00e-5
APC	MSS/WT vs MSI/WT	0·00e0	0·00e0	0·00e0	0·00e0
	MSS/WT vs MSS/MUT	3·98e-1	1·24e-2	2·26e-1	3·76e-2
	MSS/WT vs MSI/MUT	0·00e0	5·23e-2	0·00e0	0·00e0
	MSI/WT vs MSS/MUT	0·00e0	0·00e0	0·00e0	0·00e0
	MSI/WT vs MSI/MUT	3·48e-1	1·60e-1	6·80e-2	1·09e-2
	MSS/MUT vs MSI/MUT	0·00e0	8·40e-4	0·00e0	0·00e0
ATM	MSS/WT vs MSI/WT	0·00e0	0·00e0	0·00e0	0·00e0
	MSS/WT vs MSS/MUT	3·30e-2	7·90e-1	2·45e-1	1·30e-1
	MSS/WT vs MSI/MUT	0·00e0	0·00e0	0·00e0	0·00e0
	MSI/WT vs MSS/MUT	0·00e0	0·00e0	0·00e0	0·00e0
	MSI/WT vs MSI/MUT	1·65e-1	4·84e-1	3·27e-1	5·41e-1
	MSS/MUT vs MSI/MUT	0·00e0	2·00e-5	0·00e0	0·00e0
BMPR2	MSS/WT vs MSI/WT	0·00e0	0·00e0	0·00e0	0·00e0
	MSS/WT vs MSS/MUT	4·07e-1	2·62e-1	8·88e-3	8·14e-3
	MSS/WT vs MSI/MUT	0·00e0	0·00e0	0·00e0	0·00e0
	MSI/WT vs MSS/MUT	3·00e-2	7·95e-2	2·85e-2	2·85e-2
	MSI/WT vs MSI/MUT	4·06e-2	2·93e-2	1·03e-1	6·90e-2
	MSS/MUT vs MSI/MUT	9·90e-4	1·05e-3	3·93e-2	3·49e-2
BRAF	MSS/WT vs MSI/WT	0·00e0	0·00e0	0·00e0	0·00e0
	MSS/WT vs MSS/MUT	2·50e-4	0·00e0	2·70e-4	5·02e-3
	MSS/WT vs MSI/MUT	0·00e0	0·00e0	0·00e0	0·00e0
	MSI/WT vs MSS/MUT	2·12e-3	1·60e-1	0·00e0	0·00e0
	MSI/WT vs MSI/MUT	3·88e-2	3·80e-2	9·80e-1	8·40e-1
	MSS/MUT vs MSI/MUT	0·00e0	1·00e-5	0·00e0	0·00e0
CCDC40	MSS/WT vs MSI/WT	0·00e0	0·00e0	0·00e0	0·00e0
	MSS/WT vs MSS/MUT	6·97e-1	6·19e-2	3·93e-2	2·60e-2
	MSS/WT vs MSI/MUT	0·00e0	1·60e-4	0·00e0	0·00e0
	MSI/WT vs MSS/MUT	5·82e-3	3·07e-1	1·80e-4	8·10e-4
	MSI/WT vs MSI/MUT	7·35e-1	9·86e-1	3·14e-1	5·91e-1
	MSS/MUT vs MSI/MUT	3·49e-2	4·75e-1	8·58e-3	8·58e-3
CDK12	MSS/WT vs MSI/WT	0·00e0	0·00e0	0·00e0	0·00e0
	MSS/WT vs MSS/MUT	9·24e-1	2·61e-1	3·61e-2	3·61e-2
	MSS/WT vs MSI/MUT	0·00e0	5·00e-5	0·00e0	0·00e0
	MSI/WT vs MSS/MUT	3·70e-2	1·83e-1	4·70e-4	1·48e-3
	MSI/WT vs MSI/MUT	9·64e-1	9·28e-1	8·94e-1	9·92e-1
	MSS/MUT vs MSI/MUT	1·58e-2	1·77e-1	2·27e-3	2·27e-3
CHD1	MSS/WT vs MSI/WT	0·00e0	0·00e0	0·00e0	0·00e0
	MSS/WT vs MSS/MUT	1·83e-2	1·11e-1	4·92e-1	5·70e-1
	MSS/WT vs MSI/MUT	0·00e0	0·00e0	0·00e0	0·00e0
	MSI/WT vs MSS/MUT	7·10e-2	1·48e-1	1·43e-1	1·43e-1
	MSI/WT vs MSI/MUT	3·50e-2	7·10e-2	4·95e-1	2·79e-1
	MSS/MUT vs MSI/MUT	6·01e-3	2·65e-2	1·82e-1	1·82e-1
CTNND1	MSS/WT vs MSI/WT	0·00e0	0·00e0	0·00e0	0·00e0
	MSS/WT vs MSS/MUT	1·94e-1	8·61e-1	2·68e-1	3·79e-1
	MSS/WT vs MSI/MUT	0·00e0	0·00e0	0·00e0	0·00e0
	MSI/WT vs MSS/MUT	3·38e-1	1·01e-1	7·46e-2	5·70e-2
	MSI/WT vs MSI/MUT	4·60e-1	4·63e-1	3·34e-1	1·64e-1
	MSS/MUT vs MSI/MUT	2·01e-1	7·79e-2	1·25e-1	1·67e-1
DUSP16	MSS/WT vs MSI/WT	0·00e0	0·00e0	0·00e0	0·00e0
	MSS/WT vs MSS/MUT	9·73e-1	6·27e-1	6·30e-1	6·98e-1
	MSS/WT vs MSI/MUT	1·00e-5	3·04e-3	0·00e0	0·00e0
	MSI/WT vs MSS/MUT	5·30e-4	1·70e-2	1·20e-4	1·30e-4
	MSI/WT vs MSI/MUT	3·86e-1	7·59e-1	4·75e-1	3·14e-1

	MSS/MUT vs MSI/MUT	1·84e-3	9·46e-2	1·10e-3	1·10e-3
ELL2	MSS/WT vs MSI/WT	0·00e0	0·00e0	0·00e0	0·00e0
	MSS/WT vs MSS/MUT	2·12e-1	6·50e-2	4·92e-1	9·14e-1
	MSS/WT vs MSI/MUT	0·00e0	0·00e0	1·70e-4	1·40e-4
	MSI/WT vs MSS/MUT	1·96e-1	4·99e-1	1·27e-1	6·35e-2
	MSI/WT vs MSI/MUT	6·39e-1	7·96e-1	5·41e-1	2·81e-1
	MSS/MUT vs MSI/MUT	1·09e-1	6·34e-1	5·00e-1	5·00e-1
FHOD3	MSS/WT vs MSI/WT	0·00e0	0·00e0	0·00e0	0·00e0
	MSS/WT vs MSS/MUT	4·85e-1	3·54e-1	5·09e-2	4·77e-2
	MSS/WT vs MSI/MUT	0·00e0	0·00e0	0·00e0	0·00e0
	MSI/WT vs MSS/MUT	6·96e-2	1·66e-1	2·40e-2	2·40e-2
	MSI/WT vs MSI/MUT	3·79e-1	1·88e-1	4·25e-1	6·30e-1
	MSS/MUT vs MSI/MUT	4·69e-2	6·31e-2	3·37e-3	5·34e-3
hypermutated	MSS/WT vs MSI/WT	8·48e-3	2·38e-2	0·00e0	0·00e0
	MSS/WT vs MSS/MUT	9·29e-2	3·41e-2	8·56e-1	7·66e-1
	MSS/WT vs MSI/MUT	0·00e0	0·00e0	0·00e0	0·00e0
	MSI/WT vs MSS/MUT	1·26e-2	1·29e-1	1·00e-5	1·00e-5
	MSI/WT vs MSI/MUT	7·29e-1	1·00e0	1·55e-2	2·23e-2
	MSS/MUT vs MSI/MUT	0·00e0	6·00e-5	0·00e0	0·00e0
KIF1A	MSS/WT vs MSI/WT	0·00e0	0·00e0	0·00e0	0·00e0
	MSS/WT vs MSS/MUT	2·45e-1	6·07e-3	8·65e-2	1·43e-1
	MSS/WT vs MSI/MUT	0·00e0	0·00e0	0·00e0	0·00e0
	MSI/WT vs MSS/MUT	0·00e0	1·07e-1	0·00e0	0·00e0
	MSI/WT vs MSI/MUT	8·22e-1	9·58e-1	1·31e-1	7·30e-1
	MSS/MUT vs MSI/MUT	0·00e0	1·20e-1	0·00e0	0·00e0
KRAS	MSS/WT vs MSI/WT	0·00e0	0·00e0	0·00e0	0·00e0
	MSS/WT vs MSS/MUT	7·88e-3	1·80e-4	1·08e-1	1·14e-3
	MSS/WT vs MSI/MUT	8·10e-4	3·28e-1	0·00e0	1·00e-5
	MSI/WT vs MSS/MUT	0·00e0	0·00e0	0·00e0	0·00e0
	MSI/WT vs MSI/MUT	3·32e-2	1·54e-2	9·19e-1	7·27e-2
	MSS/MUT vs MSI/MUT	5·15e-3	8·98e-1	0·00e0	0·00e0
MECOM	MSS/WT vs MSI/WT	0·00e0	0·00e0	0·00e0	0·00e0
	MSS/WT vs MSS/MUT	5·54e-2	2·53e-1	7·16e-1	6·78e-1
	MSS/WT vs MSI/MUT	0·00e0	0·00e0	0·00e0	0·00e0
	MSI/WT vs MSS/MUT	4·21e-2	5·52e-2	1·55e-3	1·55e-3
	MSI/WT vs MSI/MUT	9·98e-1	3·32e-1	1·86e-1	1·37e-1
	MSS/MUT vs MSI/MUT	1·10e-2	2·13e-2	3·08e-3	5·38e-3
NRAS	MSS/WT vs MSI/WT	0·00e0	0·00e0	0·00e0	0·00e0
	MSS/WT vs MSS/MUT	2·96e-1	8·12e-1	5·81e-1	2·52e-1
	MSS/WT vs MSI/MUT	2·65e-1	3·06e-2	9·31e-1	9·53e-1
	MSI/WT vs MSS/MUT	0·00e0	9·14e-3	0·00e0	0·00e0
	MSI/WT vs MSI/MUT	9·94e-1	1·09e-1	1·55e-2	2·17e-2
	MSS/MUT vs MSI/MUT	1·86e-1	7·58e-2	8·25e-1	6·98e-1
PIK3CA	MSS/WT vs MSI/WT	0·00e0	0·00e0	0·00e0	0·00e0
	MSS/WT vs MSS/MUT	4·36e-2	7·07e-3	5·82e-1	5·41e-3
	MSS/WT vs MSI/MUT	0·00e0	0·00e0	0·00e0	0·00e0
	MSI/WT vs MSS/MUT	0·00e0	5·41e-3	0·00e0	0·00e0
	MSI/WT vs MSI/MUT	2·21e-1	8·16e-1	8·92e-3	6·45e-3
	MSS/MUT vs MSI/MUT	0·00e0	7·06e-3	0·00e0	1·00e-5
PLEKHA6	MSS/WT vs MSI/WT	0·00e0	0·00e0	0·00e0	0·00e0
	MSS/WT vs MSS/MUT	2·47e-1	8·99e-2	7·81e-3	7·81e-3
	MSS/WT vs MSI/MUT	0·00e0	0·00e0	0·00e0	0·00e0
	MSI/WT vs MSS/MUT	1·81e-3	6·66e-2	2·63e-1	3·16e-1
	MSI/WT vs MSI/MUT	2·36e-2	2·06e-1	9·12e-2	2·63e-1
	MSS/MUT vs MSI/MUT	7·70e-4	3·74e-2	3·45e-1	1·38e-1
RFX5	MSS/WT vs MSI/WT	0·00e0	0·00e0	0·00e0	0·00e0
	MSS/WT vs MSS/MUT	3·80e-1	5·63e-1	3·82e-1	2·43e-1
	MSS/WT vs MSI/MUT	0·00e0	0·00e0	0·00e0	0·00e0
	MSI/WT vs MSS/MUT	5·12e-1	1·98e-1	5·19e-3	2·60e-3
	MSI/WT vs MSI/MUT	1·09e-1	1·41e-1	6·81e-1	9·09e-1
	MSS/MUT vs MSI/MUT	3·57e-1	7·14e-2	2·56e-2	2·56e-2
RNF43	MSS/WT vs MSI/WT	0·00e0	0·00e0	0·00e0	0·00e0
	MSS/WT vs MSS/MUT	1·75e-2	3·77e-3	4·26e-3	1·30e-3
	MSS/WT vs MSI/MUT	0·00e0	0·00e0	0·00e0	0·00e0
	MSI/WT vs MSS/MUT	1·44e-3	4·93e-2	0·00e0	0·00e0
	MSI/WT vs MSI/MUT	9·92e-2	1·41e-2	5·57e-1	5·67e-1
	MSS/MUT vs MSI/MUT	0·00e0	0·00e0	0·00e0	0·00e0
SMAD2	MSS/WT vs MSI/WT	0·00e0	0·00e0	0·00e0	0·00e0
	MSS/WT vs MSS/MUT	1·74e-2	1·70e-1	1·72e-3	1·21e-3
	MSS/WT vs MSI/MUT	2·06e-1	7·18e-1	0·00e0	0·00e0
	MSI/WT vs MSS/MUT	5·60e-4	1·73e-1	0·00e0	1·00e-5
	MSI/WT vs MSI/MUT	2·80e-4	3·46e-2	7·17e-1	9·44e-1
	MSS/MUT vs MSI/MUT	6·45e-1	5·49e-1	4·00e-5	2·68e-3
SMG1	MSS/WT vs MSI/WT	0·00e0	0·00e0	0·00e0	0·00e0
	MSS/WT vs MSS/MUT	6·54e-1	2·95e-1	1·50e-3	6·50e-4
	MSS/WT vs MSI/MUT	5·00e-5	6·06e-3	0·00e0	0·00e0
	MSI/WT vs MSS/MUT	4·20e-4	1·41e-2	1·84e-3	1·01e-2
	MSI/WT vs MSI/MUT	8·39e-2	3·27e-2	5·73e-1	8·58e-1
	MSS/MUT vs MSI/MUT	3·73e-2	3·60e-1	1·97e-3	1·31e-2
TBX3	MSS/WT vs MSI/WT	0·00e0	0·00e0	0·00e0	0·00e0
	MSS/WT vs MSS/MUT	1·48e-2	1·13e-1	1·92e-1	1·52e-1
	MSS/WT vs MSI/MUT	1·00e-5	5·00e-2	0·00e0	0·00e0
	MSI/WT vs MSS/MUT	6·65e-3	9·12e-2	2·00e-5	1·00e-5
	MSI/WT vs MSI/MUT	8·56e-1	9·86e-2	2·72e-1	4·98e-1
	MSS/MUT vs MSI/MUT	4·92e-2	8·87e-1	3·00e-5	2·00e-5

TGFBR2	MSS/WT vs MSI/WT	0·00e0	0·00e0	0·00e0	0·00e0
	MSS/WT vs MSS/MUT	6·69e-1	2·51e-1	9·00e-4	3·00e-4
	MSS/WT vs MSI/MUT	0·00e0	0·00e0	0·00e0	0·00e0
	MSI/WT vs MSS/MUT	3·87e-3	2·53e-1	0·00e0	5·00e-5
	MSI/WT vs MSI/MUT	2·96e-1	8·99e-1	6·54e-1	7·13e-1
	MSS/MUT vs MSI/MUT	2·67e-2	4·74e-1	2·00e-5	1·70e-4
TP53	MSS/WT vs MSI/WT	0·00e0	2·70e-4	0·00e0	0·00e0
	MSS/WT vs MSS/MUT	3·12e-3	0·00e0	7·00e-5	0·00e0
	MSS/WT vs MSI/MUT	0·00e0	3·28e-1	0·00e0	0·00e0
	MSI/WT vs MSS/MUT	0·00e0	0·00e0	0·00e0	0·00e0
	MSI/WT vs MSI/MUT	7·54e-1	4·44e-2	4·26e-1	7·67e-2
	MSS/MUT vs MSI/MUT	0·00e0	1·00e-5	0·00e0	0·00e0
TRPS1	MSS/WT vs MSI/WT	0·00e0	0·00e0	0·00e0	0·00e0
	MSS/WT vs MSS/MUT	4·41e-1	3·69e-2	1·00e0	6·80e-1
	MSS/WT vs MSI/MUT	0·00e0	1·10e-4	0·00e0	0·00e0
	MSI/WT vs MSS/MUT	1·00e-5	4·90e-2	4·00e-5	1·20e-4
	MSI/WT vs MSI/MUT	6·67e-1	5·97e-1	3·72e-2	6·18e-2
	MSS/MUT vs MSI/MUT	1·26e-3	2·26e-1	5·00e-5	2·20e-4
WNT16	MSS/WT vs MSI/WT	0·00e0	0·00e0	0·00e0	0·00e0
	MSS/WT vs MSS/MUT	2·77e-1	4·55e-1	9·38e-2	1·25e-1
	MSS/WT vs MSI/MUT	0·00e0	0·00e0	0·00e0	0·00e0
	MSI/WT vs MSS/MUT	6·23e-1	5·41e-1	3·77e-2	3·77e-2
	MSI/WT vs MSI/MUT	5·31e-1	8·69e-1	5·72e-1	7·24e-1
	MSS/MUT vs MSI/MUT	4·44e-1	4·44e-1	1·43e-1	1·43e-1
ZHX2	MSS/WT vs MSI/WT	0·00e0	0·00e0	0·00e0	0·00e0
	MSS/WT vs MSS/MUT	7·25e-1	1·51e-1	2·67e-2	1·83e-2
	MSS/WT vs MSI/MUT	2·90e-4	4·90e-4	0·00e0	0·00e0
	MSI/WT vs MSS/MUT	1·79e-3	1·11e-1	2·02e-3	9·00e-4
	MSI/WT vs MSI/MUT	1·69e-1	6·65e-1	8·09e-1	7·59e-1
	MSS/MUT vs MSI/MUT	3·69e-2	2·57e-1	8·58e-3	3·19e-3
ZNRF3	MSS/WT vs MSI/WT	0·00e0	0·00e0	0·00e0	0·00e0
	MSS/WT vs MSS/MUT	9·27e-1	3·96e-1	9·89e-2	1·91e-1
	MSS/WT vs MSI/MUT	0·00e0	0·00e0	0·00e0	0·00e0
	MSI/WT vs MSS/MUT	1·50e-3	1·30e-4	0·00e0	0·00e0
	MSI/WT vs MSI/MUT	1·83e-1	3·52e-1	1·52e-1	1·27e-1
	MSS/MUT vs MSI/MUT	1·80e-4	1·00e-5	0·00e0	1·00e-5

Tab. S17: Performance metrics of Multi-Target Transformers for the MSI subgroup in external validation. Mean and standard deviation from the 7 folds of the cross-validation for relevant selected prediction targets in the MSI subgroup. The threshold for binary classification is pre-defined as 0·5· The evaluation metrics include the Matthews Correlation Coefficient (MCC), the Area Under the Receiver Operating Characteristic Curve (AUROC), and the Area Under the Precision-Recall Curve (AUPRC), along with the corresponding mutation rates in external cohorts. Mutation rate contains the ratio of mutations of the respective target within the external data set. The data is sorted for Target and visualized in Fig. S3B.

**Target**	**Accuracy**	**Precision**	**Sensitivity**	**Specificity**	**F1 Score**	**MCC**	**AUROC**	**AUPRC**	**Mutation Rate**
ACVR1B	0·2 (±0·05)	0·11 (±0·01)	0·91 (±0·05)	0·11 (±0·05)	0·2 (±0·01)	0·01 (±0·06)	0·6 (±0·04)	0·19 (±0·04)	0·11
AKT1	0·2 (±0·07)	0·11 (±0·0)	0·91 (±0·08)	0·12 (±0·08)	0·2 (±0·01)	0·03 (±0·04)	0·55 (±0·04)	0·15 (±0·03)	0·11
ALK	0·25 (±0·02)	0·22 (±0·01)	0·92 (±0·08)	0·05 (±0·05)	0·36 (±0·02)	-0·06 (±0·06)	0·44 (±0·03)	0·24 (±0·05)	0·23
APC	0·64 (±0·03)	0·53 (±0·22)	0·26 (±0·19)	0·84 (±0·14)	0·28 (±0·16)	0·12 (±0·05)	0·6 (±0·02)	0·43 (±0·03)	0·33
ATM	0·28 (±0·05)	0·21 (±0·01)	0·9 (±0·07)	0·11 (±0·09)	0·34 (±0·01)	0·02 (±0·05)	0·47 (±0·03)	0·2 (±0·02)	0·21
BMPR2	0·56 (±0·02)	0·58 (±0·01)	0·89 (±0·08)	0·1 (±0·05)	0·7 (±0·03)	-0·01 (±0·04)	0·44 (±0·03)	0·54 (±0·01)	0·58
BRAF	0·57 (±0·02)	0·56 (±0·01)	0·91 (±0·07)	0·17 (±0·07)	0·7 (±0·02)	0·12 (±0·04)	0·49 (±0·02)	0·55 (±0·02)	0·54
CCDC40	0·2 (±0·09)	0·1 (±0·01)	0·81 (±0·21)	0·13 (±0·13)	0·18 (±0·03)	-0·04 (±0·06)	0·46 (±0·04)	0·13 (±0·04)	0·11
CDK12	0·21 (±0·04)	0·14 (±0·01)	0·91 (±0·06)	0·1 (±0·05)	0·25 (±0·01)	0·01 (±0·05)	0·5 (±0·04)	0·16 (±0·02)	0·14
CHD1	0·35 (±0·12)	0·18 (±0·01)	0·91 (±0·15)	0·25 (±0·17)	0·3 (±0·01)	0·16 (±0·03)	0·6 (±0·06)	0·22 (±0·07)	0·06
CTNND1	0·24 (±0·04)	0·13 (±0·01)	0·78 (±0·06)	0·15 (±0·05)	0·22 (±0·02)	-0·07 (±0·09)	0·42 (±0·04)	0·13 (±0·01)	0·14
DUSP16	0·14 (±0·07)	0·07 (±0·0)	0·95 (±0·07)	0·08 (±0·07)	0·13 (±0·0)	0·03 (±0·03)	0·58 (±0·05)	0·11 (±0·04)	0·07
ELL2	0·22 (±0·1)	0·05 (±0·0)	0·95 (±0·13)	0·18 (±0·11)	0·1 (±0·0)	0·08 (±0·03)	0·65 (±0·06)	0·1 (±0·02)	0·02
FHOD3	0·52 (±0·04)	0·48 (±0·02)	0·84 (±0·13)	0·25 (±0·15)	0·61 (±0·04)	0·13 (±0·08)	0·54 (±0·03)	0·49 (±0·03)	0·16
hypermutated	0·85 (±0·04)	0·92 (±0·0)	0·91 (±0·04)	0·0 (±0·0)	0·92 (±0·02)	-0·08 (±0·02)	0·34 (±0·05)	0·9 (±0·01)	0·93
KIF1A	0·26 (±0·03)	0·22 (±0·01)	0·91 (±0·1)	0·07 (±0·06)	0·36 (±0·02)	-0·02 (±0·06)	0·51 (±0·04)	0·28 (±0·03)	0·23
KRAS	0·82 (±0·06)	0·14 (±0·11)	0·17 (±0·11)	0·89 (±0·08)	0·14 (±0·1)	0·05 (±0·1)	0·6 (±0·05)	0·17 (±0·06)	0·1
MECOM	0·39 (±0·04)	0·34 (±0·02)	0·78 (±0·09)	0·17 (±0·07)	0·47 (±0·04)	-0·06 (±0·12)	0·42 (±0·04)	0·32 (±0·03)	0·13
NRAS	0·86 (±0·09)	0·06 (±0·06)	0·64 (±0·38)	0·87 (±0·09)	0·11 (±0·1)	0·17 (±0·15)	0·81 (±0·16)	0·11 (±0·07)	0·01
PIK3CA	0·26 (±0·02)	0·23 (±0·01)	0·81 (±0·08)	0·08 (±0·05)	0·35 (±0·02)	-0·15 (±0·06)	0·38 (±0·04)	0·22 (±0·04)	0·25
PLEKHA6	0·49 (±0·04)	0·46 (±0·02)	0·9 (±0·08)	0·19 (±0·07)	0·61 (±0·03)	0·13 (±0·11)	0·57 (±0·05)	0·47 (±0·04)	0·16
RFX5	0·3 (±0·07)	0·18 (±0·01)	0·91 (±0·13)	0·17 (±0·11)	0·3 (±0·02)	0·09 (±0·06)	0·51 (±0·06)	0·19 (±0·02)	0·06
RNF43	0·68 (±0·02)	0·71 (±0·01)	0·9 (±0·05)	0·17 (±0·07)	0·79 (±0·02)	0·1 (±0·07)	0·53 (±0·01)	0·71 (±0·02)	0·69
SMAD2	0·11 (±0·03)	0·07 (±0·0)	0·97 (±0·06)	0·05 (±0·04)	0·14 (±0·01)	0·02 (±0·05)	0·51 (±0·07)	0·11 (±0·04)	0·07
SMG1	0·22 (±0·09)	0·11 (±0·01)	0·87 (±0·16)	0·14 (±0·12)	0·2 (±0·02)	0·03 (±0·08)	0·48 (±0·06)	0·12 (±0·02)	0·11

TBX3	0·21 (±0·05)	0·16 (±0·0)	0·96 (±0·07)	0·07 (±0·07)	0·28 (±0·0)	0·06 (±0·02)	0·53 (±0·05)	0·2 (±0·03)	0·16
TGFBR2	0·25 (±0·04)	0·18 (±0·0)	0·89 (±0·08)	0·11 (±0·06)	0·3 (±0·01)	0·0 (±0·03)	0·51 (±0·03)	0·21 (±0·01)	0·18
TP53	0·71 (±0·08)	0·4 (±0·16)	0·17 (±0·2)	0·88 (±0·16)	0·19 (±0·1)	0·09 (±0·07)	0·58 (±0·03)	0·32 (±0·02)	0·25
TRPS1	0·37 (±0·08)	0·31 (±0·03)	0·94 (±0·05)	0·13 (±0·12)	0·47 (±0·02)	0·09 (±0·1)	0·58 (±0·07)	0·38 (±0·07)	0·11
WNT16	0·38 (±0·11)	0·22 (±0·02)	0·81 (±0·15)	0·27 (±0·17)	0·34 (±0·03)	0·08 (±0·08)	0·53 (±0·03)	0·24 (±0·02)	0·07
ZHX2	0·19 (±0·04)	0·09 (±0·01)	0·87 (±0·09)	0·12 (±0·06)	0·17 (±0·01)	-0·01 (±0·05)	0·48 (±0·06)	0·11 (±0·02)	0·1
ZNRF3	0·36 (±0·01)	0·33 (±0·01)	0·84 (±0·06)	0·11 (±0·05)	0·47 (±0·01)	-0·08 (±0·03)	0·44 (±0·02)	0·32 (±0·02)	0·34

Tab. S18: Performance metrics of Multi-Target Transformers for the MSS subgroup in external validation. Mean and standard deviation from the 7 folds of the cross-validation for relevant selected prediction targets in the MSS subgroup. The threshold for binary classification is pre-defined as 0·5· The evaluation metrics include the Matthews Correlation Coefficient (MCC), the Area Under the Receiver Operating Characteristic Curve (AUROC), and the Area Under the Precision-Recall Curve (AUPRC), along with the corresponding mutation rates in external cohorts. Mutation rate contains the ratio of mutations of the respective target within the external data set. The data is sorted for Target and visualized in Fig. S3B.

Target	Accuracy	Precision	Sensitivity	Specificity	F1 Score	MCC	AUROC	AUPRC	Mutation Rate
ACVR1B	0·77 (±0·1)	0·11 (±0·03)	0·64 (±0·21)	0·77 (±0·11)	0·19 (±0·05)	0·2 (±0·06)	0·77 (±0·05)	0·21 (±0·08)	0·04
AKT1	0·59 (±0·22)	0·04 (±0·01)	0·64 (±0·26)	0·59 (±0·23)	0·07 (±0·01)	0·08 (±0·02)	0·67 (±0·04)	0·05 (±0·01)	0·02
ALK	0·49 (±0·31)	0·03 (±0·01)	0·58 (±0·34)	0·49 (±0·32)	0·06 (±0·01)	0·03 (±0·03)	0·58 (±0·03)	0·04 (±0·01)	0·03
APC	0·62 (±0·09)	0·72 (±0·03)	0·76 (±0·24)	0·32 (±0·27)	0·71 (±0·15)	0·09 (±0·06)	0·56 (±0·05)	0·73 (±0·04)	0·69
ATM	0·73 (±0·1)	0·03 (±0·01)	0·16 (±0·09)	0·76 (±0·11)	0·05 (±0·02)	-0·04 (±0·02)	0·42 (±0·02)	0·05 (±0·02)	0·05
BMPR2	0·83 (±0·08)	0·03 (±0·01)	0·71 (±0·3)	0·83 (±0·08)	0·06 (±0·03)	0·12 (±0·06)	0·87 (±0·08)	0·05 (±0·02)	0·01
BRAF	0·79 (±0·04)	0·13 (±0·03)	0·28 (±0·11)	0·83 (±0·05)	0·17 (±0·05)	0·08 (±0·04)	0·63 (±0·03)	0·15 (±0·02)	0·08
CCDC40	0·78 (±0·12)	0·02 (±0·01)	0·54 (±0·34)	0·79 (±0·12)	0·04 (±0·02)	0·07 (±0·05)	0·77 (±0·07)	0·03 (±0·01)	0·01
CDK12	0·74 (±0·09)	0·02 (±0·01)	0·57 (±0·28)	0·74 (±0·09)	0·04 (±0·01)	0·07 (±0·04)	0·75 (±0·08)	0·03 (±0·01)	0·01
CHD1	0·87 (±0·06)	0·0 (±0·01)	0·14 (±0·38)	0·87 (±0·06)	0·01 (±0·02)	0·0 (±0·06)	0·71 (±0·15)	0·02 (±0·01)	0·0
CTNND1	0·83 (±0·08)	0·02 (±0·01)	0·43 (±0·16)	0·83 (±0·08)	0·03 (±0·01)	0·06 (±0·02)	0·63 (±0·11)	0·04 (±0·03)	0·01
DUSP16	0·57 (±0·23)	0·01 (±0·01)	0·46 (±0·34)	0·58 (±0·23)	0·02 (±0·01)	0·01 (±0·05)	0·55 (±0·11)	0·01 (±0·0)	0·01
ELL2	0·84 (±0·08)	0·0 (±0·0)	0·14 (±0·38)	0·84 (±0·09)	0·0 (±0·01)	-0·01 (±0·04)	0·53 (±0·24)	0·01 (±0·0)	0·0
FHOD3	0·85 (±0·06)	0·09 (±0·02)	0·45 (±0·19)	0·87 (±0·07)	0·14 (±0·02)	0·15 (±0·04)	0·68 (±0·07)	0·12 (±0·03)	0·01
hypermutated	0·76 (±0·08)	0·03 (±0·01)	0·18 (±0·07)	0·78 (±0·08)	0·05 (±0·02)	-0·01 (±0·03)	0·49 (±0·05)	0·03 (±0·0)	0·03
KIF1A	0·54 (±0·26)	0·07 (±0·01)	0·6 (±0·23)	0·53 (±0·28)	0·12 (±0·02)	0·07 (±0·03)	0·58 (±0·04)	0·07 (±0·01)	0·05
KRAS	0·53 (±0·05)	0·46 (±0·03)	0·6 (±0·2)	0·47 (±0·21)	0·51 (±0·07)	0·08 (±0·06)	0·57 (±0·04)	0·5 (±0·04)	0·42
MECOM	0·84 (±0·06)	0·01 (±0·01)	0·1 (±0·16)	0·85 (±0·06)	0·01 (±0·02)	-0·02 (±0·04)	0·55 (±0·09)	0·02 (±0·01)	0·01
NRAS	0·48 (±0·17)	0·05 (±0·01)	0·48 (±0·21)	0·48 (±0·19)	0·09 (±0·02)	-0·02 (±0·03)	0·47 (±0·05)	0·06 (±0·02)	0·06
PIK3CA	0·57 (±0·13)	0·16 (±0·02)	0·55 (±0·2)	0·58 (±0·18)	0·25 (±0·04)	0·09 (±0·05)	0·59 (±0·03)	0·16 (±0·01)	0·13
PLEKHA6	0·84 (±0·06)	0·03 (±0·01)	1·0 (±0·0)	0·84 (±0·06)	0·06 (±0·02)	0·15 (±0·04)	0·99 (±0·01)	0·48 (±0·38)	0·0
RFX5	0·81 (±0·09)	0·0 (±0·0)	0·0 (±0·0)	0·82 (±0·1)	0·0 (±0·0)	-0·04 (±0·01)	0·3 (±0·13)	0·01 (±0·0)	0·0
RNF43	0·81 (±0·06)	0·07 (±0·01)	0·31 (±0·12)	0·83 (±0·06)	0·12 (±0·02)	0·07 (±0·03)	0·69 (±0·02)	0·08 (±0·01)	0·04
SMAD2	0·52 (±0·21)	0·07 (±0·02)	0·69 (±0·15)	0·51 (±0·23)	0·12 (±0·03)	0·09 (±0·04)	0·68 (±0·03)	0·13 (±0·05)	0·04
SMG1	0·71 (±0·17)	0·04 (±0·02)	0·67 (±0·24)	0·71 (±0·17)	0·07 (±0·03)	0·1 (±0·03)	0·79 (±0·08)	0·07 (±0·03)	0·01

TBX3	0·65 (±0·16)	0·02 (±0·01)	0·41 (±0·28)	0·65 (±0·16)	0·04 (±0·02)	0·02 (±0·05)	0·6 (±0·07)	0·04 (±0·01)	0·02
TGFBR2	0·79 (±0·09)	0·07 (±0·01)	0·6 (±0·21)	0·79 (±0·09)	0·13 (±0·02)	0·15 (±0·04)	0·76 (±0·05)	0·08 (±0·01)	0·03
TP53	0·62 (±0·03)	0·72 (±0·04)	0·68 (±0·14)	0·52 (±0·18)	0·69 (±0·05)	0·21 (±0·06)	0·66 (±0·03)	0·75 (±0·03)	0·64
TRPS1	0·51 (±0·2)	0·04 (±0·02)	0·51 (±0·16)	0·51 (±0·21)	0·08 (±0·03)	0·01 (±0·06)	0·52 (±0·09)	0·05 (±0·01)	0·02
WNT16	0·87 (±0·08)	0·0 (±0·0)	0·0 (±0·0)	0·88 (±0·08)	0·0 (±0·0)	-0·02 (±0·01)	0·12 (±0·11)	0·0 (±0·0)	0·0
ZHX2	0·76 (±0·06)	0·02 (±0·01)	0·57 (±0·29)	0·76 (±0·06)	0·05 (±0·02)	0·08 (±0·05)	0·76 (±0·05)	0·03 (±0·01)	0·01
ZNRF3	0·82 (±0·05)	0·0 (±0·0)	0·02 (±0·04)	0·84 (±0·05)	0·0 (±0·01)	-0·05 (±0·02)	0·4 (±0·07)	0·02 (±0·0)	0·02

Tab. S19: Pathological review of top tiles for selected slides. The slides correspond to those used for creating the heatmaps. We selected the top and bottom slides based on the prediction score (highest and lowest) for the genetic alteration. All of these tiles were assigned high attention by the model and are therefore considered to be of high relevance for the resulting slide-based prediction score. Sorted by Slide Name. Abbreviations: HM: Hypermutation; MSI: Microsatellite instability; MSS: Microsatellite stability; MUT: Mutated; NOS: Not otherwise specified; TILs: Tumor infiltrating lymphocytes; WT: Wild type

Slide Name	Alteration	Ground Truth	Prediction Score	Tiles shown	Histopathological Assessment
CRA, 5661	MSS/MSI	MSI	0·99	top	medullary carcinoma (solid growth, sheets of tumor cells, high number of TILs)
CRA, 5661	TP53	MUT	0·18	bottom	some medullary carcinoma, some connective tissue with prominent vessels, some lymphoid aggregates, some inconspicuous glands
CRA, 5694	MSS/MSI	MSI	0·06	bottom	gland-forming adenocarcinoma (NOS) with dirty necrosis
CRA, 5694	TP53	MUT	0·69	top	gland-forming adenocarcinoma (NOS) with dirty necrosis
CRA, 5733	MSS/MSI	MSS	0·02	bottom	gland-forming adenocarcinoma (NOS)
CRA, 5733	TP53	WT	0·7	top	gland-forming adenocarcinoma (NOS), connective tissue and smooth muscles
WHI, 1031546	BRAF	WT	0·96	top	medullary carcinoma (solid growth, sheets of tumor cells, high number of TILs)
WHI, 1031546	MSS/MSI	MSI	0·98	top	medullary carcinoma (solid growth, sheets of tumor cells, high number of TILs)
WHI, 1031550	KRAS	WT	0·09	bottom	medullary and mucinous differentiation
WHI, 1031550	MSS/MSI	MSI	0·99	top	medullary carcinoma (solid growth, sheets of tumor cells, high number of TILs)
WHI, 1031553	HM	WT	0·89	top	mucinous differentiation
WHI, 1031553	MSS/MSI	MSI	0·91	top	mucinous differentiation
WHI, 1031557	BRAF	WT	0·95	top	medullary and mucinous differentiation
WHI, 1031557	HM	WT	0·95	top	medullary and mucinous differentiation
WHI, 1031557	MSS/MSI	MSI	0·99	top	mucinous and more solid differentiation, high number of TILs
WHI, 1031567	HM	WT	0·75	top	medullary and mucinous differentiation, TILs, connective tissue with prominent vessels
WHI, 1031567	MSS/MSI	MSI	0·88	top	medullary and mucinous differentiation, TILs
WHI, 1031576	HM	WT	0·63	top	lymphoid aggregates, normal mucosa, adipocytes
WHI, 1031576	MSS/MSI	MSI	0·85	top	lymphoid aggregates, normal mucosa, adipocytes
WHI, 1031609	HM	MUT	0·98	top	medullary carcinoma (solid growth, sheets of tumor cells, high number of TILs)
WHI, 1031609	MSS/MSI	MSI	0·99	top	medullary carcinoma (solid growth, sheets of tumor cells, high number of TILs)
WHI, 1031622	TP53	WT	0·11	bottom	mucinous differentiation
WHI,	MSS/MSI	MSI	0·98	top	mucinous differentiation

Slide Name	Alteration	Ground Truth	Prediction Score	Tiles shown	Histopathological Assessment
1031622					
WHI, 1031632	MSS/MSI	MSI	0·98	top	mucinous and medullary differentiation
WHI, 1031632	RNF43	WT	0·93	top	mucinous and medullary differentiation
WHI, 1031662	APC	MUT	0·17	bottom	adipocytes, mucin (without relevant tumor content), lymphoid aggregates
WHI, 1031662	MSS/MSI	MSS	0·93	top	mucinous differentiation, TILs
WHI, 1031666	APC	MUT	0·35	bottom	mucinous differentiation, lymphoid aggregates, connective tissue
WHI, 1031666	MSS/MSI	MSS	0·96	top	partly mucinous differentiation, TILs, also conventional gland-forming adenocarcinoma and few normal glands
WHI, 1031666	RNF43	WT	0·91	top	mucinous differentiation, normal glands with lymphocytic background, connective tissue
WHI, 1031672	KRAS	MUT	0·82	top	dysplastic epithelium, superficial tumor parts
WHI, 1031672	MSS/MSI	MSS	0·27	bottom	dysplastic epithelium
WHI, 1031705	BRAF	WT	0·06	bottom	dysplastic epithelium, superficial tumor parts
WHI, 1031705	MSS/MSI	MSS	0·04	bottom	dysplastic epithelium, superficial tumor parts
WHI, 1031733	BRAF	MUT	0·97	top	medullary carcinoma (solid growth, sheets of tumor cells, high number of TILs)
WHI, 1031733	MSS/MSI	MSI	0·97	top	medullary carcinoma (solid growth, sheets of tumor cells, high number of TILs)
WHI, 1031786	KRAS	MUT	0·14	bottom	lymphoid aggregates, few tumor glands, few normal glands, connective tissue, adipocytes
WHI, 1031786	MSS/MSI	MSI	0·95	top	medullary carcinoma (solid growth, sheets of tumor cells, high number of TILs), mucus
WHI, 1031792	KRAS	WT	0·08	bottom	sheets of tumor cells, TILs
WHI, 1031792	MSS/MSI	MSI	0·98	top	sheets of tumor cells, TILs
WHI, 1031829	KRAS	WT	0·75	top	dysplastic epithelium
WHI, 1031829	MSS/MSI	MSS	0·05	bottom	dysplastic epithelium
WHI, 1031846	KRAS	WT	0·65	top	gland-forming adenocarcinoma (NOS)
WHI, 1031846	MSS/MSI	MSI	0·55	top	mucinous differentiation
WHI, 1031849	BRAF	MUT	0·09	bottom	dysplastic epithelium, tumor glands with dirty necrosis (adenocarcinoma NOS)
WHI, 1031849	MSS/MSI	MSS	0·08	bottom	dysplastic epithelium, tumor glands

Tab. S20: Shapiro-Wilk test for normal distribution of mean prediction scores presented in Fig. 4. Abbreviations: MSI: Microsatellite instability; MSS: Microsatellite stability; MUT: Mutated; WT: Wild type.

Target	WT/MUT	MSS/MSI	Score Distribution	Statistic	p-value
hypermutated	WT	MSS	MSI	0·78	<0·0001
hypermutated	WT	MSS	hypermutated	0·87	<0·0001
hypermutated	WT	MSI	MSI	0·88	0·0741
hypermutated	WT	MSI	hypermutated	0·93	0·3123
hypermutated	MUT	MSS	MSI	0·81	0·0071
hypermutated	MUT	MSS	hypermutated	0·84	0·0184
hypermutated	MUT	MSI	MSI	0·78	<0·0001
hypermutated	MUT	MSI	hypermutated	0·77	<0·0001
BRAF	WT	MSS	MSI	0·77	<0·0001
BRAF	WT	MSS	BRAF	0·83	<0·0001
BRAF	WT	MSI	MSI	0·74	<0·0001
BRAF	WT	MSI	BRAF	0·78	<0·0001
BRAF	MUT	MSS	MSI	0·87	0·0004
BRAF	MUT	MSS	BRAF	0·93	0·0126
BRAF	MUT	MSI	MSI	0·82	<0·0001
BRAF	MUT	MSI	BRAF	0·89	<0·0001
TP53	WT	MSS	MSI	0·83	<0·0001
TP53	WT	MSS	TP53	0·98	0·0037
TP53	WT	MSI	MSI	0·78	<0·0001
TP53	WT	MSI	TP53	0·93	<0·0001
TP53	MUT	MSS	MSI	0·76	<0·0001
TP53	MUT	MSS	TP53	0·98	<0·0001
TP53	MUT	MSI	MSI	0·71	<0·0001
TP53	MUT	MSI	TP53	0·92	0·0064
APC	WT	MSS	MSI	0·81	<0·0001
APC	WT	MSS	APC	0·95	<0·0001
APC	WT	MSI	MSI	0·75	<0·0001
APC	WT	MSI	APC	0·94	<0·0001
APC	MUT	MSS	MSI	0·77	<0·0001
APC	MUT	MSS	APC	0·94	<0·0001
APC	MUT	MSI	MSI	0·78	<0·0001
APC	MUT	MSI	APC	0·95	0·0102
BMPR2	WT	MSS	MSI	0·78	<0·0001
BMPR2	WT	MSS	BMPR2	0·83	<0·0001
BMPR2	WT	MSI	MSI	0·71	<0·0001
BMPR2	WT	MSI	BMPR2	0·72	<0·0001
BMPR2	MUT	MSS	MSI	Insufficient data	Insufficient data
BMPR2	MUT	MSS	BMPR2	Insufficient data	Insufficient data
BMPR2	MUT	MSI	MSI	0·80	<0·0001
BMPR2	MUT	MSI	BMPR2	0·83	<0·0001
RNF43	WT	MSS	MSI	0·77	<0·0001
RNF43	WT	MSS	RNF43	0·81	<0·0001
RNF43	WT	MSI	MSI	0·78	<0·0001
RNF43	WT	MSI	RNF43	0·84	<0·0001
RNF43	MUT	MSS	MSI	0·92	0·1023
RNF43	MUT	MSS	RNF43	0·94	0·2874
RNF43	MUT	MSI	MSI	0·76	<0·0001
RNF43	MUT	MSI	RNF43	0·82	<0·0001
ZNRF3	WT	MSS	MSI	0·79	<0·0001
ZNRF3	WT	MSS	ZNRF43	0·85	<0·0001
ZNRF3	WT	MSI	MSI	0·76	<0·0001
ZNRF3	WT	MSI	ZNRF43	0·86	<0·0001
ZNRF3	MUT	MSS	MSI	0·87	0·129
ZNRF3	MUT	MSS	ZNRF43	0·95	0·6615
ZNRF3	MUT	MSI	MSI	0·78	<0·0001
ZNRF3	MUT	MSI	ZNRF43	0·85	<0·0001

KRAS	WT	MSS	MSI	0·76	<0·0001
KRAS	WT	MSS	KRAS	0·98	0·0031
KRAS	WT	MSI	MSI	0·76	<0·0001
KRAS	WT	MSI	KRAS	0·95	<0·0001
KRAS	MUT	MSS	MSI	0·81	<0·0001
KRAS	MUT	MSS	KRAS	0·99	0·0422
KRAS	MUT	MSI	MSI	0·80	0·0022
KRAS	MUT	MSI	KRAS	0·98	0·95

Supplementary Figures

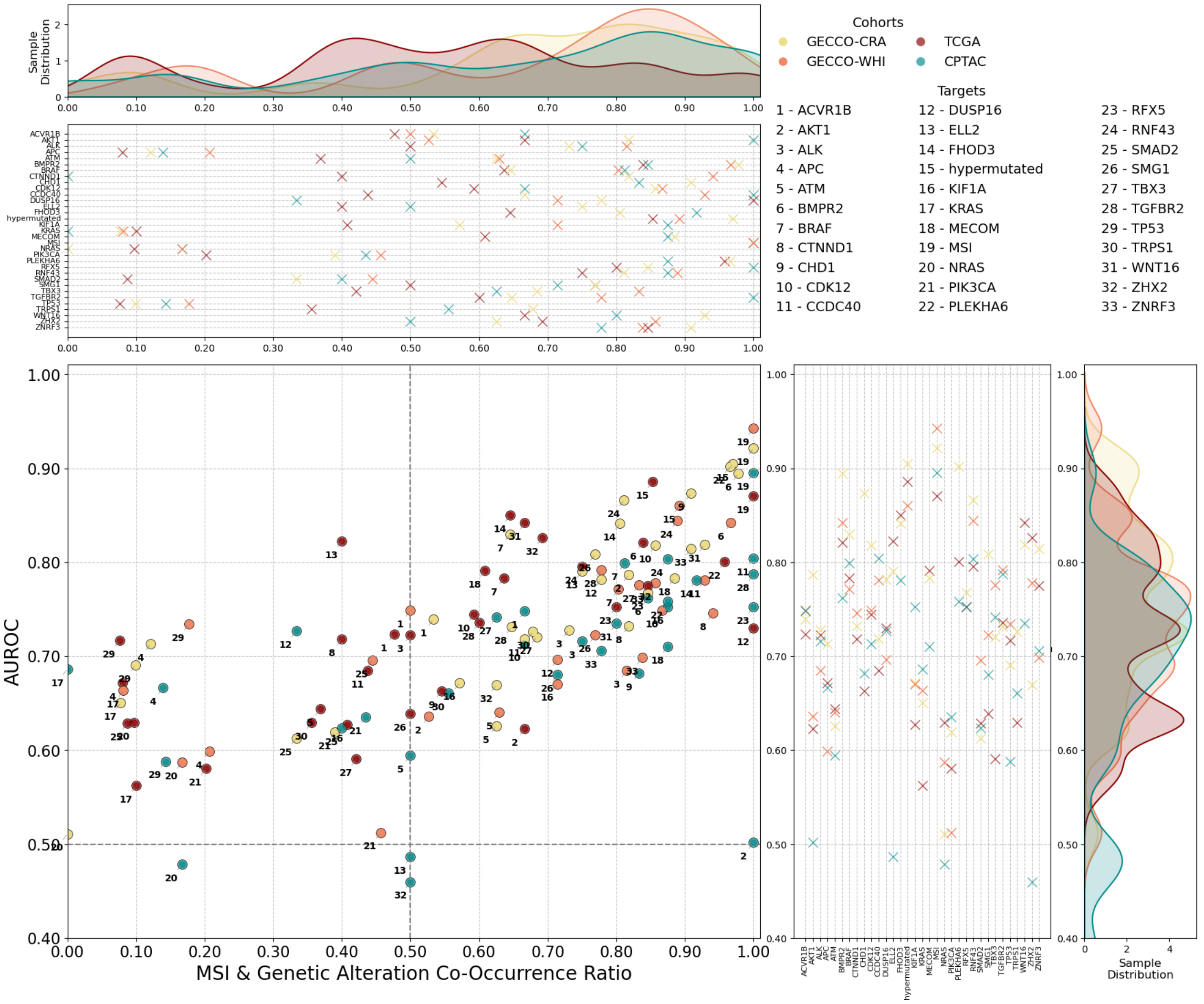

Fig. S1: **Performance evaluation of the primary multi-target transformer on all four external cohorts.** The relationship between the MSI & Genetic Alteration Co-Occurrence Ratio, representing the fraction of cases harboring MSI among those with a specific mutation, and the mean AUROCs from seven folds, reflecting predictive performance, is shown. Each dot corresponds to a prediction target, with IDs mapped to genetic alterations in the legend. Density plots on the top and right illustrate the distribution of co-occurrence ratios and AUROC values between cohorts, respectively. The horizontal and vertical cross distributions, aligned with the target names on the axes, improve clarity of target positions. Further metrics provided in Tab. S5-S8.

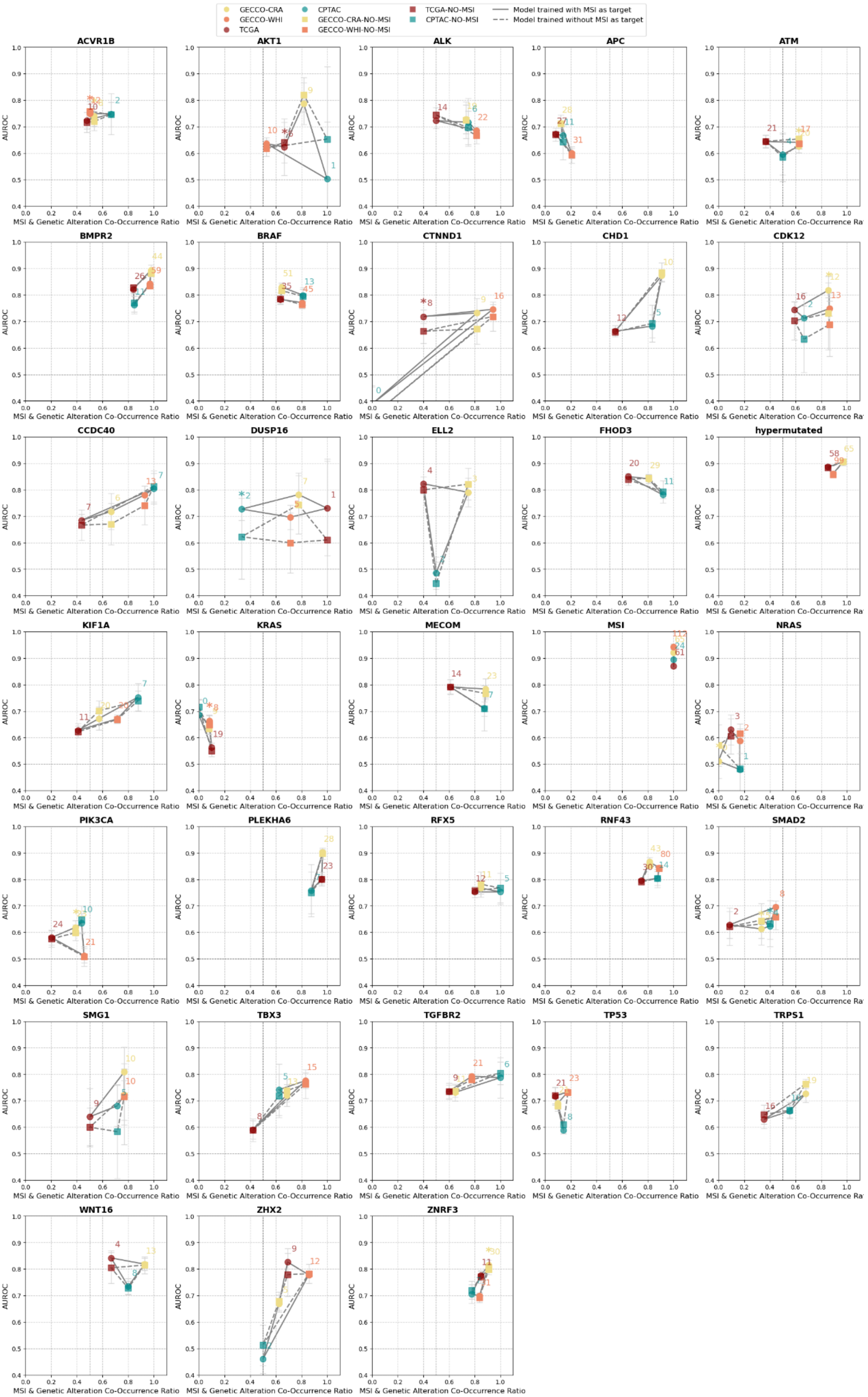

Fig. S2: **Performance comparison of the primary multi-target transformer including MSI as a target with the secondary multi-target transformer excluding MSI as a target on all four external cohorts**. The association between MSI and Genetic Alteration Co-Occurrence Ratio—defined as the proportion of cases exhibiting MSI among those with a given mutation—and the

mean AUROCs with standard deviations across seven folds, are presented on a target basis. Each cohort is represented by a distinct color. Dots with continuous lines denote results for the primary model, while squares with dashed lines correspond to the secondary model. Absolute case numbers with concurrent MSI and target mutations are provided for context. Statistical significance, determined via the DeLong test ($p<0{\cdot}05$), is indicated by asterisks. Further metrics provided in Tab. S5-S14.

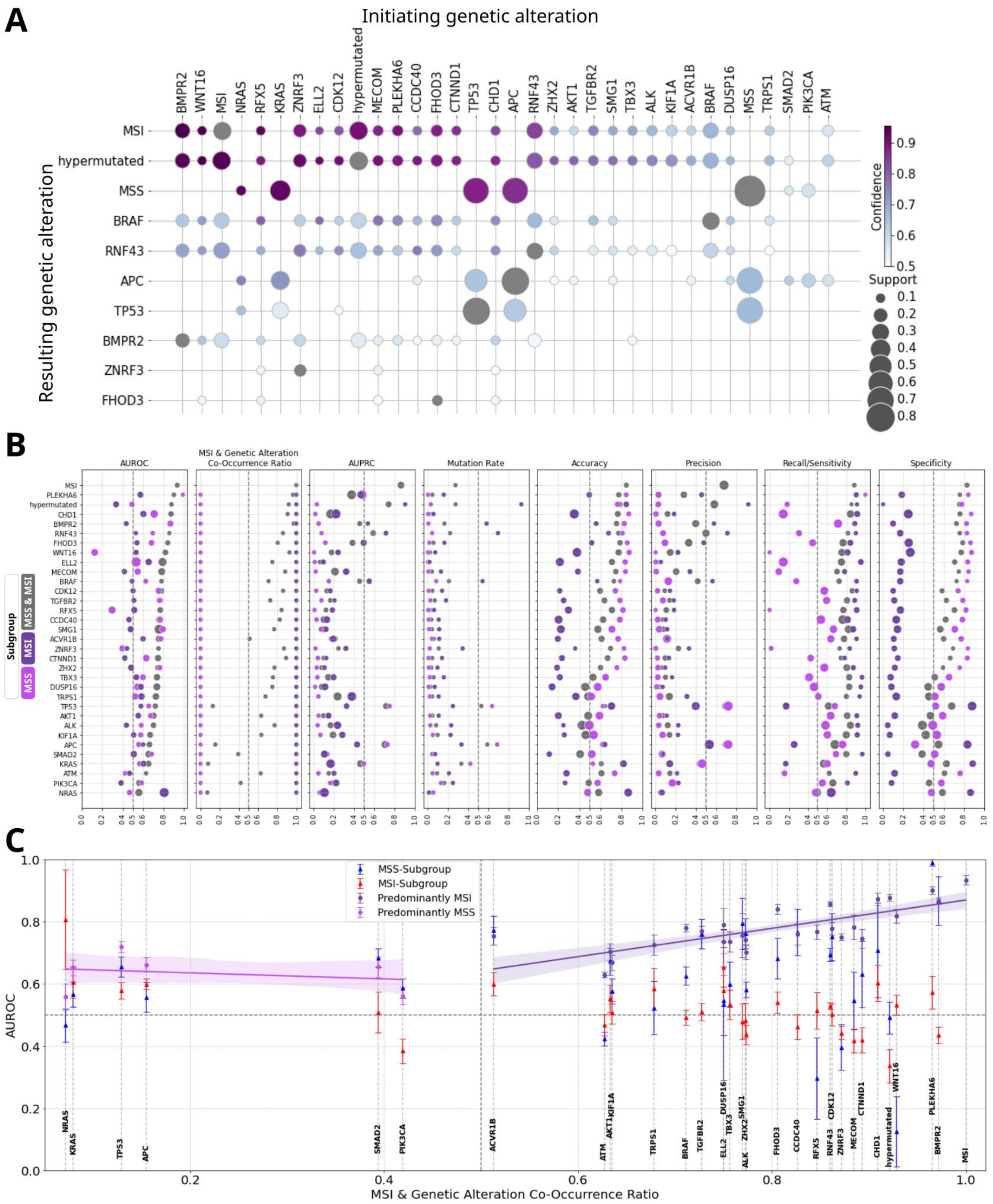

Fig. S3: **Co-Occurrence of genetic alterations and the performance of Multi-Target Transformers on selected alterations. A**. Association rule mining was used to investigate genetic alterations (Tab. S13). The analysis includes an initiating genetic alteration ('antecedent') and resulting genetic alteration ('consequent'). The presence of an initiating alteration statistically increases the probability of observing a resulting alteration, without implying a cause-effect biological sequence. Confidence, calculated as the ratio of co-occurrences to instances of the antecedent alone, indicates the predictive power of the initiating alteration for the resulting alteration. Support measures the frequency of an alteration or combination thereof in the dataset. Gray circles represent the antecedent support, with a support value of 1 indicating that every sample in the dataset has the respective genetic alteration. Microsatellite stability (MSS) and MSI were included to demonstrate relationships with targets commonly associated with MSS. **B.**

Performance metrics of Multi-Target Transformers for external validation. The mean and standard deviation for relevant selected prediction targets for the whole external set, as well as the MSI and MSS subgroups, are displayed based on the 7 folds of cross-validation. The threshold for binary classification is pre-defined as 0·50· The evaluation metrics include the Area Under the Receiver Operating Characteristic Curve (AUROC), and the Area Under the Precision-Recall Curve (AUPRC), along with the corresponding mutation rates in external cohorts. The Mutation Rate refers to the fraction of instances with a specific mutation in the subgroup. The MSI & Genetic Alteration Co-Occurrence Ratio is the fraction of cases harboring MSI among all cases with a particular genetic mutation. The data is sorted for AUROC and shown in Tab. S14 and Tab. S17-S18. **C.** Distribution of AUROCs (mean ± standard deviation) for selected prediction targets and their co-occurrence with MSI in external validation. For comparability, the MSS/MSI subgroup-specific AUROCs are reported for the MSI & Genetic Alteration Co-Occurrence Ratio of the respective alteration in the entire external dataset. The corresponding values and further metrics shown in Tab. S14 and Tab. S17-S18.

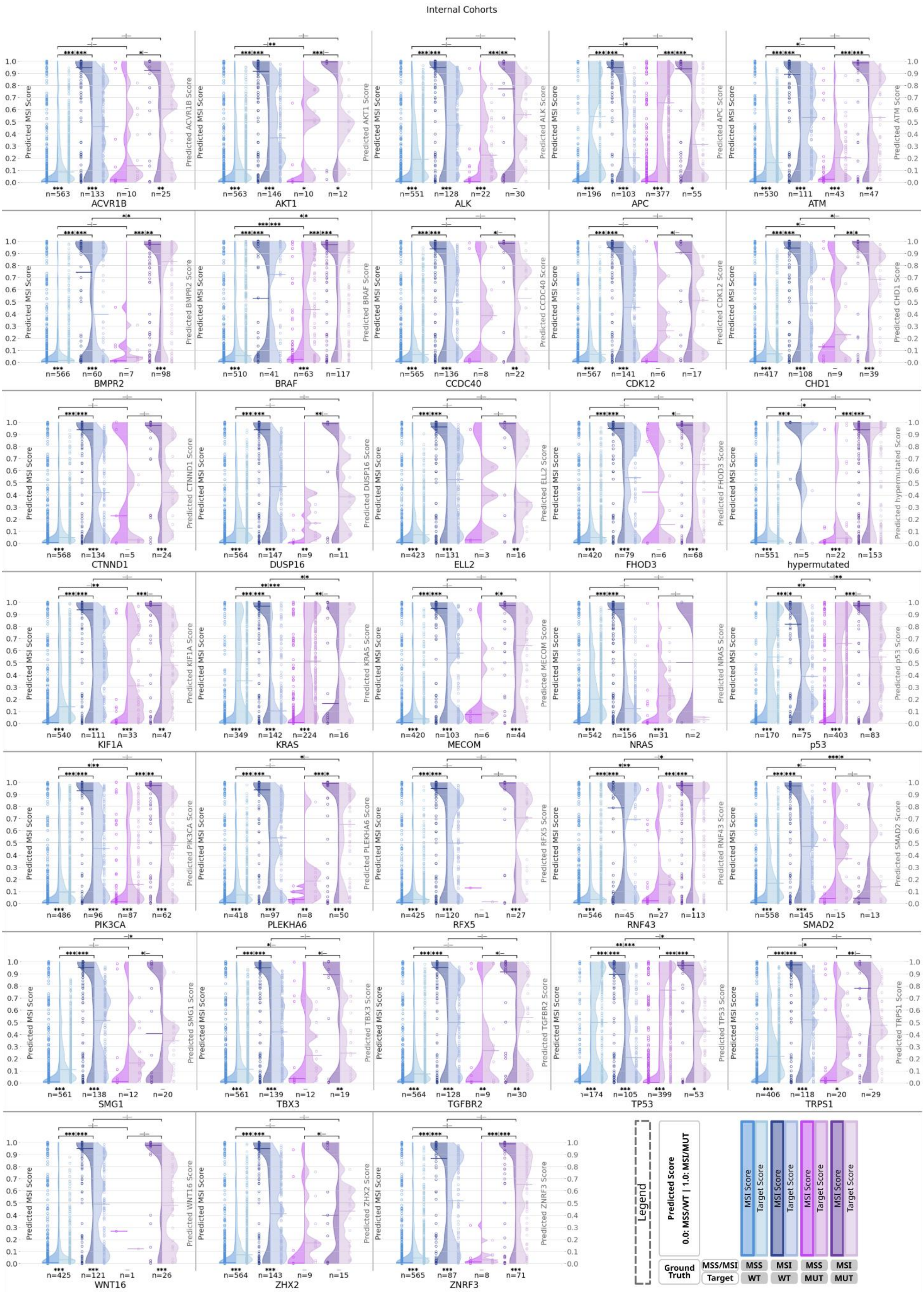

Fig. S4: **Violin plots representing individual patient scores from the train set cohorts for MSI and respective genetic alterations in four subgroups based on microsatellite and alteration mutational status.** The left y-axis represents the MSI score scale (left violins) and the right y-axis corresponds to the prediction target scores (right violins). Representative genetic alterations from genetic Cluster 1-3 were selected, as per Fig. 2. The data encompasses the train set cohorts

(EPIC, CORSA, IWHS) (Fig. 1C). Each dot represents the mean value of individual patient prediction scores calculated from 7 folds, with the horizontal line within each half violin indicating the median of all individual mean patient scores. A horizontal line at 0·50 denotes the line of model uncertainty. The sample count for each subgroup is indicated below the violins. Statistical significance is denoted in the figures as follows: * for $p < 0{\cdot}05$, ** for $p < 0{\cdot}01$, *** for $p < 0{\cdot}001$, with more details provided in Fig. 1D. The Mann-Whitney U test was used for within-group comparisons, and the Wilcoxon test was used for between-group comparisons.

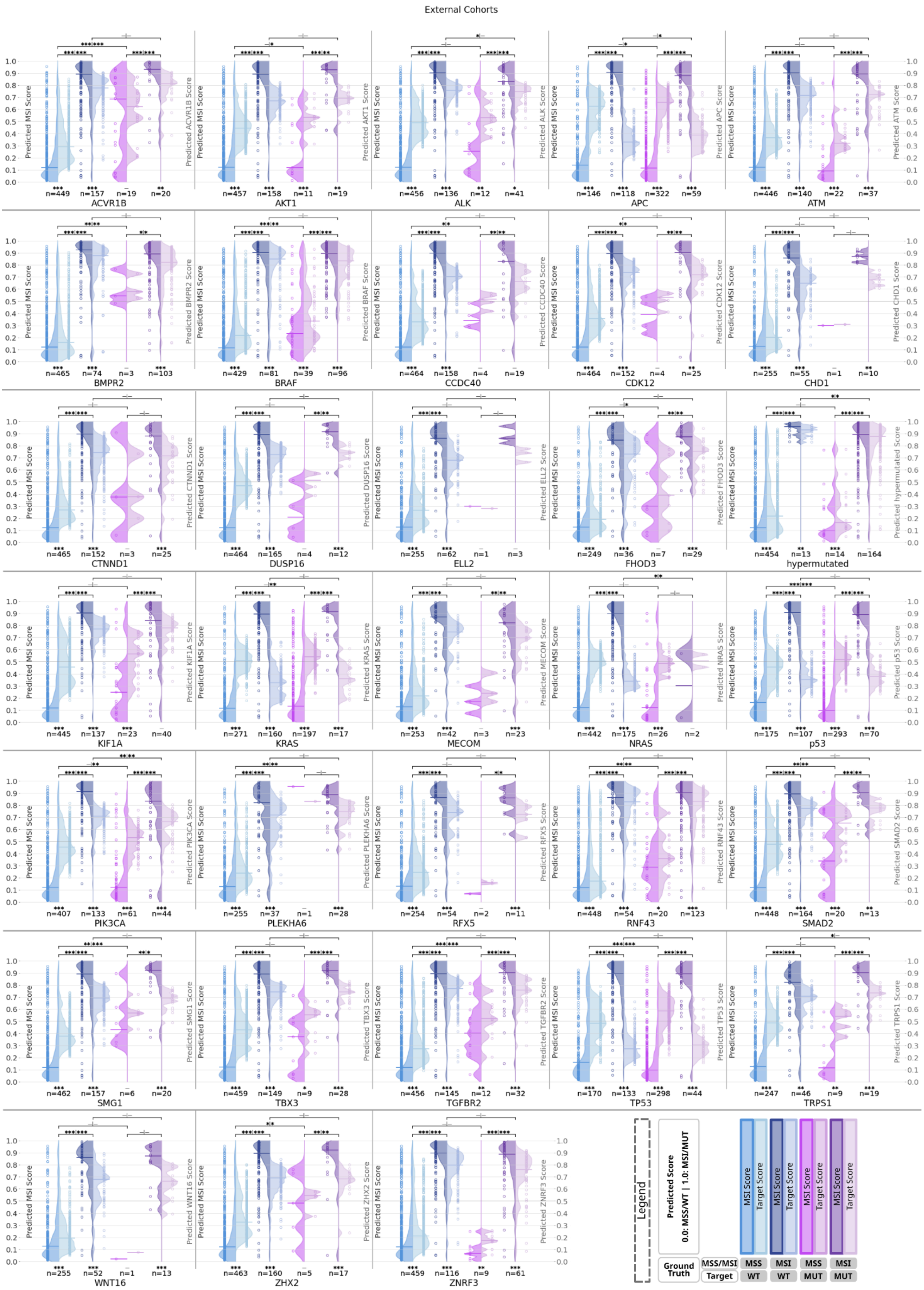

Fig. S5: **Violin plots representing individual patient scores from the test set cohorts for MSI and respective genetic alterations in four subgroups based on microsatellite and alteration mutational status.** The left y-axis represents the MSI score scale (left violins) and the right y-axis corresponds to the prediction target scores (right violins). Representative genetic alterations from genetic Cluster 1-3 were selected, as per Fig. 2. The data encompasses the test set cohorts (CRA,

WHI) (Fig. 1C). Each dot represents the mean value of individual patient prediction scores calculated from 7 folds, with the horizontal line within each half violin indicating the median of all individual mean patient scores. A horizontal line at 0·50 denotes the line of model uncertainty. The sample count for each subgroup is indicated below the violins. Statistical significance is denoted in the figures as follows: * for $p < 0{\cdot}05$, ** for $p < 0{\cdot}01$, *** for $p < 0{\cdot}001$, with more details provided in Fig. 1D. The Mann-Whitney U test was used for within-group comparisons, and the Wilcoxon test was used for between-group comparisons.

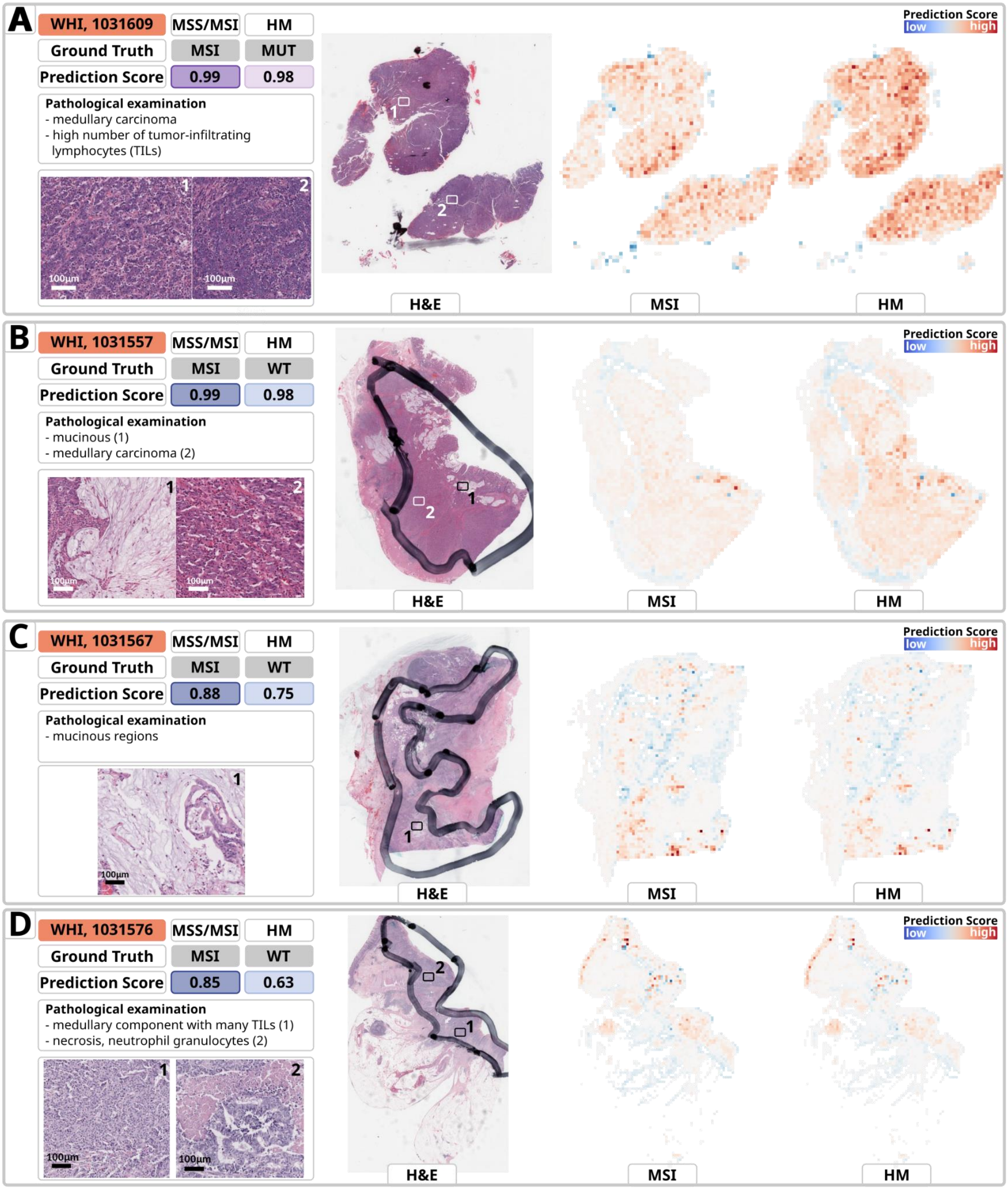

Fig. S6: **Heatmaps of representative samples for prediction of MSI and hypermutation (HM) from the external validation dataset**. The heatmaps are derived from the model with the median AUROC for MSI detection and the majority of prediction targets evaluated by sevenfold cross-validation. The cohort, Sample-ID, ground truth and prediction scores for MSI, along with the HM status, a brief pathological evaluation and magnified views of specific areas are provided for in-depth analysis. The heatmaps show critical tumor areas for predicting MSI (middle) and HM (right). Red signifies high importance and indicates MSI or HM (MUT), while blue signifies low importance and indicates MSS and HM wild type (WT). The color intensity represents the model's attention to that particular area. The sections highlighted in the heatmaps represent tumor tissue and show a high degree of similarity for MSI and HM, indicating that most of the relevant information for both prediction targets is concentrated in the same areas leading to a similar score. Minor variations in the highlighted areas of the heatmaps do not reveal noteworthy differences in pathological

assessment, as the tissue in these areas shows similar morphological patterns. **A.** This relatively homogeneous tumor shows sheets of tumor cells and a high number of tumor-infiltrating lymphocytes (TILs), consistent with the diagnosis of a medullary carcinoma. This observation is consistent with the correct prediction of MSI and HM mutations, as these are commonly seen in this type of cancer. **B.-D.** Cases of MSI and wild-type HM displaying elevated scores of both MSI and HM. **B.** MSI-like morphology with mucinous (zoom 1) and solid medullary parts (zoom 2). **C.** Tumor with a mucinous component is shown where the MSI prediction is correct and plausible but the HM WT is predicted with a high score. **D.** Higher degree of intratumoral heterogeneity, which comprises different distinct components, including a medullary component with numerous TILs (zoom 1) and gland-forming areas, necrosis, and neutrophil granulocytes (zoom 2).

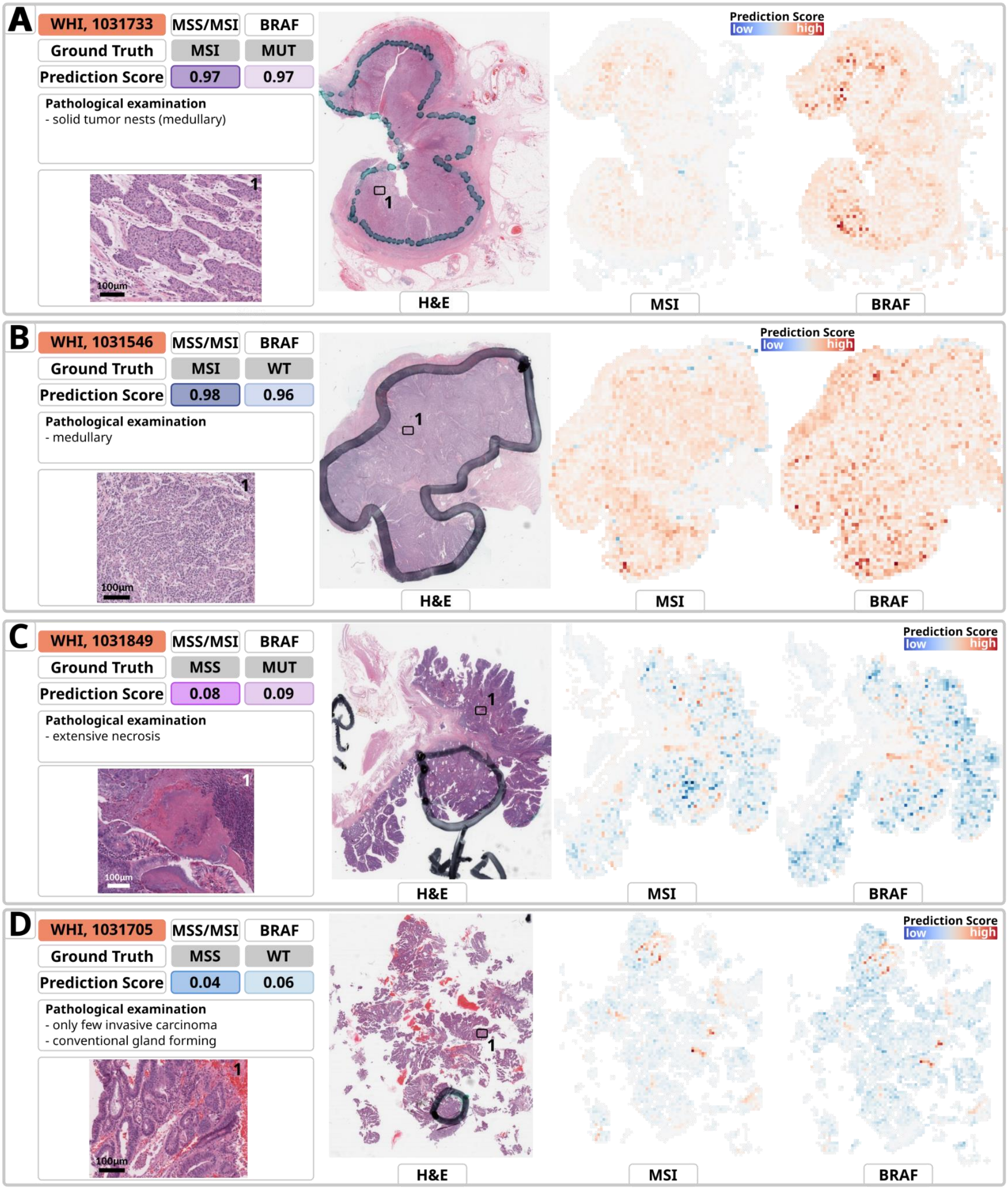

Fig. S7: **Heatmaps of representative samples for prediction of MSI and *BRAF* from the external validation dataset**. The heatmaps are derived from the model with the median AUROC for MSI detection and the majority of prediction targets evaluated by sevenfold cross-validation. The cohort, Sample-ID, ground truth and prediction scores for MSI, along with the *BRAF* mutational status, a brief pathological evaluation and magnified views of specific areas are provided for in-depth analysis. The heatmaps highlight relevant tumor areas for MSI (middle) and *BRAF* mutation (right) prediction. Red signifies high importance and indicates MSI or *BRAF* mutation (MUT), while blue signifies low importance and indicates MSS and *BRAF* wild type (WT). The color intensity represents the model's attention to that particular area. The sections highlighted in the heatmaps represent tumor tissue and show a high degree of similarity for MSI and *BRAF*, indicating that most of the relevant information for both prediction targets is concentrated in the same areas leading to

a similar score. Slight deviations in the heatmaps do not indicate any significant variations in a pathological evaluation. **A**. The solid tumor nests are typical of a medullary carcinoma. This observation is consistent with the correct prediction of MSI and *BRAF* mutations, as these are commonly seen in this type of cancer. **B**. The typical morphology indicative of medullary microsatellite insatiable tumor is apparent, providing further support for the prediction of MSI. However, despite a high MSI prediction score, a high score for *BRAF* MUT was obtained even though ground truth is *BRAF* WT. **C**. The extensive necrosis and MSS-like morphology in a MSS, *BRAF* MUT case is potentially responsible for the low prediction score for *BRAF* indicating *BRAF* WT. **D**. The heatmap indicates the existence of numerous superficial components (adenoma) and only a small number of invasive carcinoma components. It is worth noting that the invasive carcinoma features a typical gland-forming morphology resembling that of MSS, which plausibly leads to low MSI and low *BRAF* scores, correctly indicating MSS but falsely indicating *BRAF* WT.

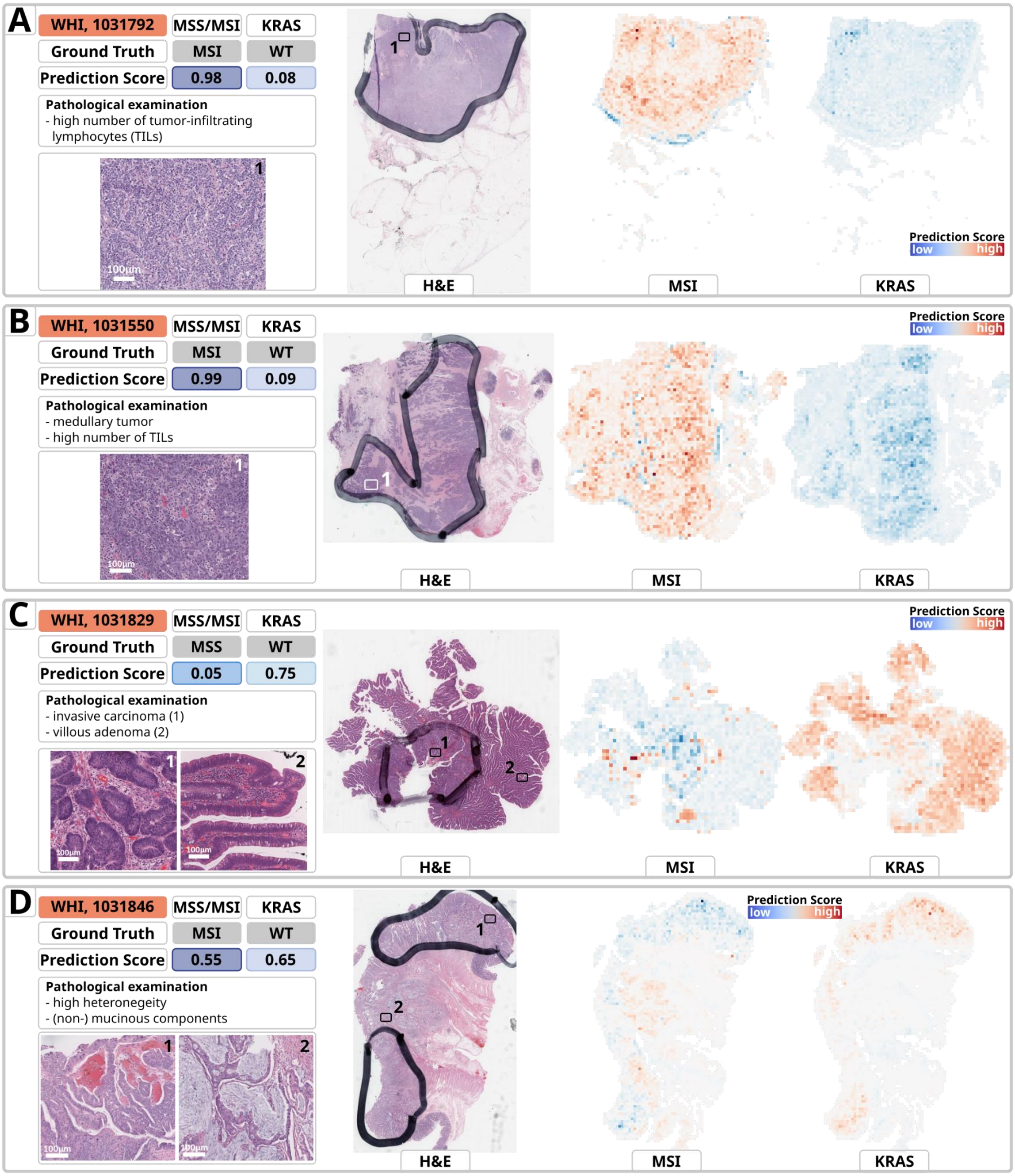

Fig. S8: **Heatmaps of representative samples for prediction of MSI and *KRAS* from the external validation dataset**. The heatmaps are derived from the model with the median AUROC for MSI detection and the majority of prediction targets evaluated by sevenfold cross-validation. The cohort, Sample-ID, ground truth and prediction scores for MSI, along with the *KRAS* mutational status, a brief pathological evaluation and magnified views of specific areas are provided for in-depth analysis. The heatmaps highlight relevant tumor areas for MSI (middle) and *KRAS* mutation (right) prediction. Red signifies high importance and indicates MSI or *KRAS* mutation (MUT), while blue signifies low importance and indicates MSS and *KRAS* wild type (WT). The color intensity represents the model's attention to that particular area. The heatmaps highlight sections of tumor tissue highly similar for MSI and *KRAS*. Nevertheless, corresponding regions in the heatmaps result in divergent scores for MSI (red) and *KRAS* (blue). In certain cases, different regions seem

to be crucial for the forecasts of MSI and *KRAS*, respectively. **A.-B.** The tumor has a solid medullary morphology with high number intratumoral lymphocytes, which is quite typical for MSI. The prediction for MSI appears to be driven by the presence of tumor sheets and a high number of TILs, and KRAS is correctly predicted with a low score indicating *KRAS* WT. Identical tumor areas are taken into consideration for predicting each target. **C.** The extensive villous adenoma frequently contains *KRAS* mutations. It is noteworthy that the *KRAS* prediction heatmap focuses on the adenoma parts rather than the invasive carcinoma, which seems more relevant for MSS prediction. While the tissue exhibits MSS-like morphology, resulting in a correspondingly low MSI score, the model yields a relatively high *KRAS* score, falsely indicating *KRAS* MUT. **D.** For predicting MSI, the mucinous component is highlighted whereas for predicting *KRAS*, the superficial tumor parts (without mucin) are highlighted. Due to the heterogeneous nature of the tumor, the predictions seem difficult, resulting in intermediate MSI and *KRAS* scores, indicating model uncertainty.

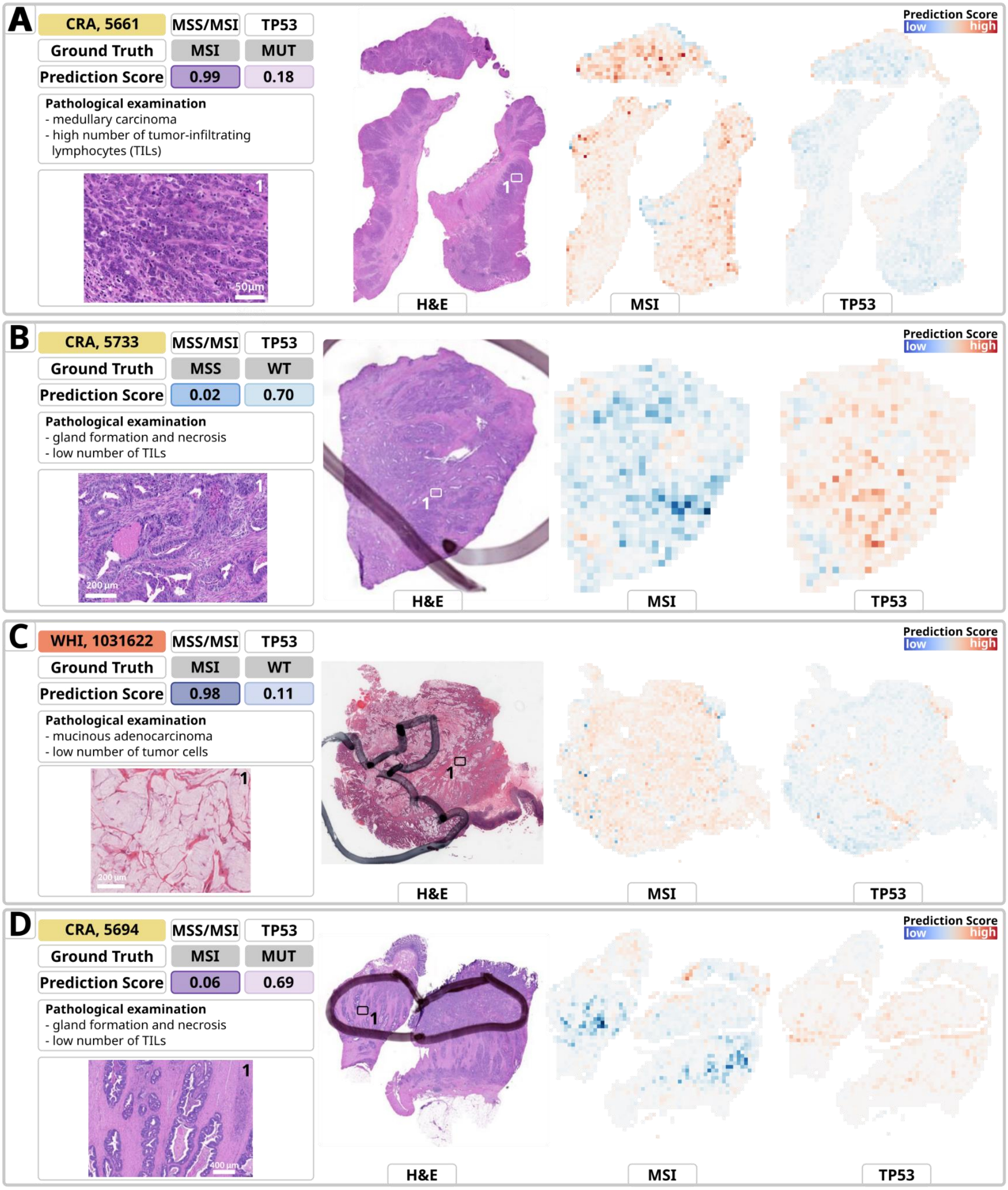

Fig. S9: **Heatmaps of representative samples for prediction of MSI and *TP53* from the external validation dataset**. The heatmaps are derived from the model with the median AUROC for MSI detection and the majority of prediction targets evaluated by sevenfold cross-validation. The cohort, Sample-ID, ground truth and prediction scores for MSI, along with the *TP53* mutational status, a brief pathological evaluation and magnified views of specific areas are provided for in-depth analysis. The heatmaps highlight relevant tumor areas for MSI (middle) and *TP53* mutation (right) prediction. Red signifies high importance and indicates MSI or *TP53* mutation (MUT), while blue signifies low importance and indicates MSS and *TP53* wild type (WT). The color intensity represents the model's attention to that particular area. The heatmaps highlight sections of tumor tissue highly similar for MSI and *TP53.* Nevertheless, corresponding regions in the heatmaps result in divergent scores for MSI (red) and *TP53* (blue). In certain cases, different regions seem to be of

higher relevance for the forecasts of MSI and *TP53*, respectively. **A.** The heatmaps highlight tumor, rather than stroma, for MSI prediction, which is plausible and reassuring. Typical morphological features of MSI CRCs include sheets of tumor cells (medullary carcinoma) with a high number of tumor-infiltrating lymphocytes (TILs). Similar regions are highlighted for MSI and *TP53* prediction, with a low prediction score indicating *TP53* WT, despite the ground truth being *TP53* MUT. **B.** The tumor appears to be MSS showing typical morphological features for MSS in CRC, including conventional histology with gland formation (NOS, ‘not otherwise specified’) and necrosis, as well as a low number of TILs. Consequently, the low MSI predictive score looks plausible, but the high *TP53* predictive score contradicts the ground truth, which is *TP53* WT. **C.** Mucinous adenocarcinoma as seen in this sample is a typical morphological feature of MSI CRCs, characterized by over 50% mucinous differentiation with mucin lakes present and only occasional tumor cells. The MSI score is high and *TP53* score is low, both indicating the same as the ground truth. **D.** Higher intensity is observed for MSS in the heatmap. The morphological features are typical of MSS colorectal cancers, despite the ground truth being MSI. The conventional histology includes gland formation (NOS, ‘not otherwise specified’) and necrosis, as well as a low number of TILs. Therefore, the pathological examination results in a plausible similar outcome to the model, even though it contradicts the ground truth of MSI.

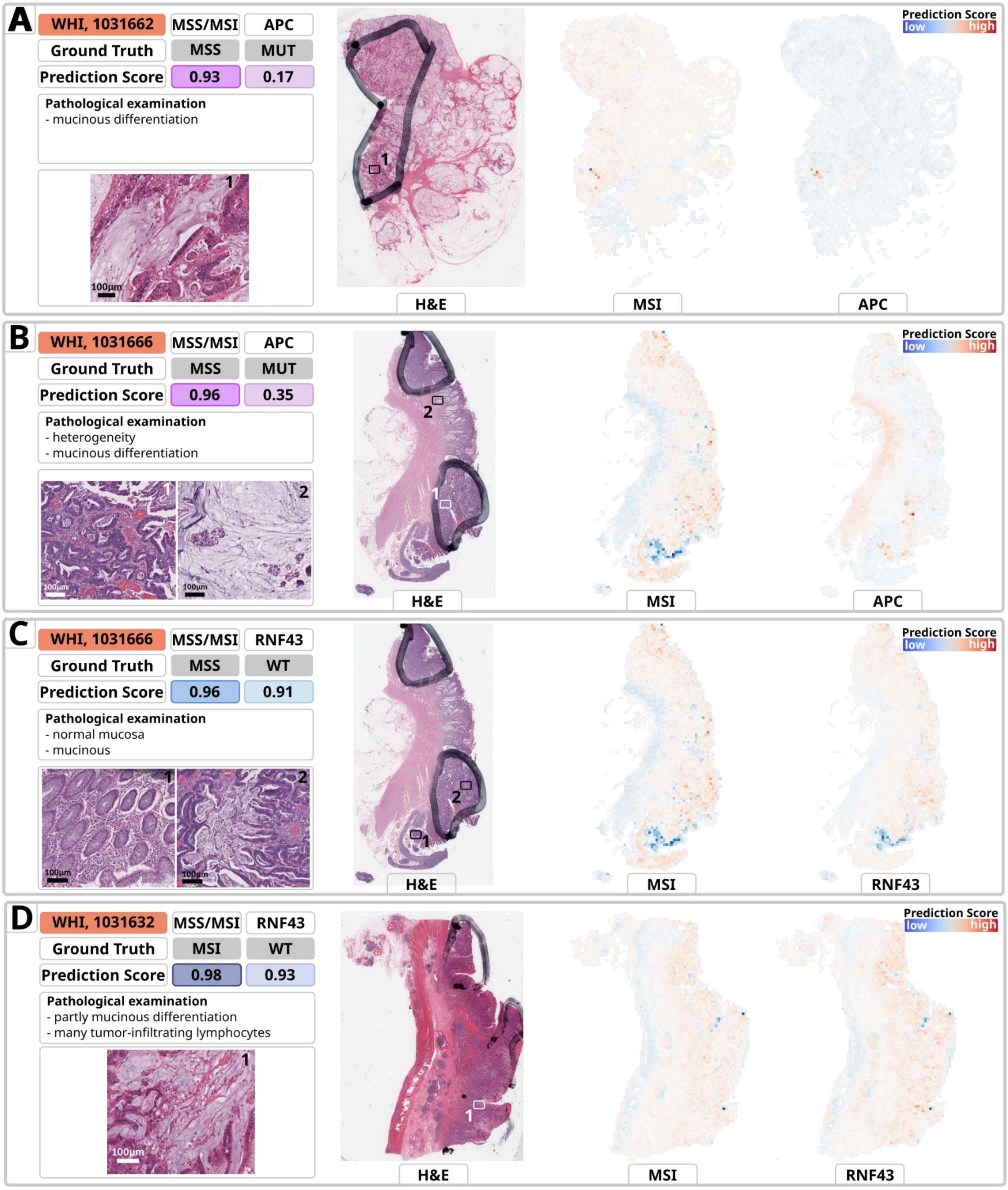

Fig. S10: **Heatmaps of representative samples for prediction of MSI, *APC* and *RNF43* from the external validation dataset**. The heatmaps are derived from the model with the median AUROC for MSI detection and the majority of prediction targets evaluated by sevenfold cross-validation. The cohort, Sample-ID, ground truth and prediction scores for MSI, along with the *APC* (**A**.-**B**.) and *RNF43* (**C**.-**D**.) mutational status, a brief pathological evaluation and magnified views of specific areas are provided for in-depth analysis. The heatmaps highlight relevant tumor areas for MSI (middle) and *APC/RNF43* mutation (right) prediction. Red signifies high importance and indicates MSI or *APC/RNF43* mutation (MUT), while blue signifies low importance and indicates MSS and *APC/RNF43* wild type (WT). The color intensity represents the model's attention to that particular area. All heatmaps highlight relatively concentrated sections of tumor tissue and are highly similar for MSI and *APC/RNF43*, respectively. However, while not only the regions but also

the colors representing the scores are highly similar for MSI and *RNF43*, corresponding regions in the heatmaps for MSI and *APC* result in divergent colors and consequently also prediction scores. **A.-B.** The slide shows a case of mucinous adenocarcinoma with a rather heterogeneous appearance in B. The MSI score is high, despite the ground truth being MSS. On the other hand, the score for APC is low, indicating APC WT, although it is mutated. **C.** This case comprises normal mucosa and mucinous differentiation. Although the ground truth is WT, RNF43 is predicted with a high score, implying mutation, and despite being MSS, it receives a high prediction score indicating MSI. **D.** This example shows a case with partial mucinous differentiation and a high number of tumor infiltrating lymphocytes (TILs). The high MSI prediction is pathologically plausible and reflects the ground truth. Despite being WT, RNF43 receives a high score.

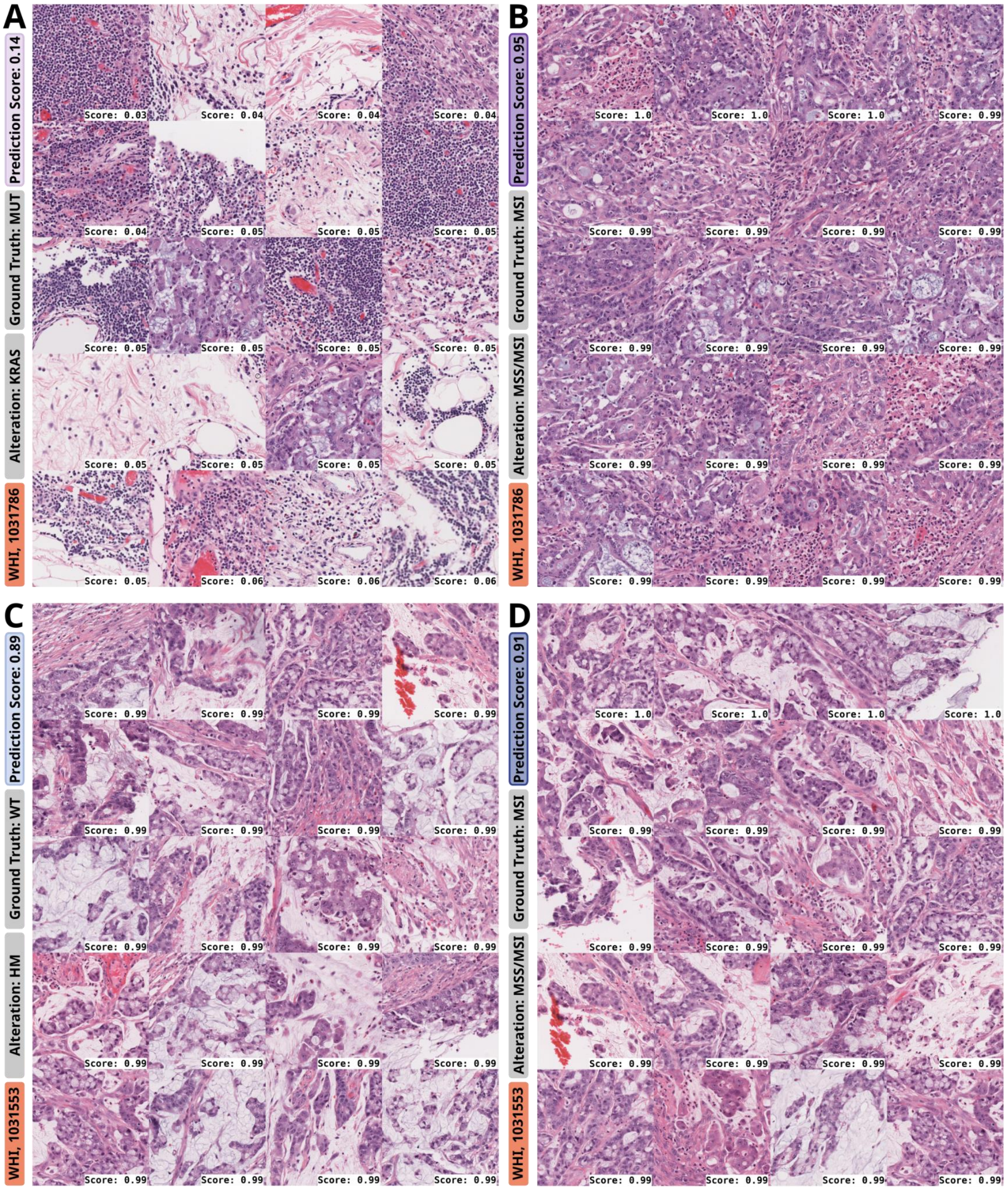

Fig. S11: **Top tiles for prediction of genetic alterations and MSI for two selected slides from Fig. 5.** Each row includes top tiles for one slide (heatmaps in Fig. 5), with tiles for KRAS (**A.**-**B.**)/Hypermutation (HM) (**C.**-**D.**) in the left and MSS/MSI in the right column. A detailed pathological assessment is given in Tab. S19. Abbreviations: HM: Hypermutation; MSI: Microsatellite instability; MSS: Microsatellite stability; MUT: Mutated; WT: Wild type.

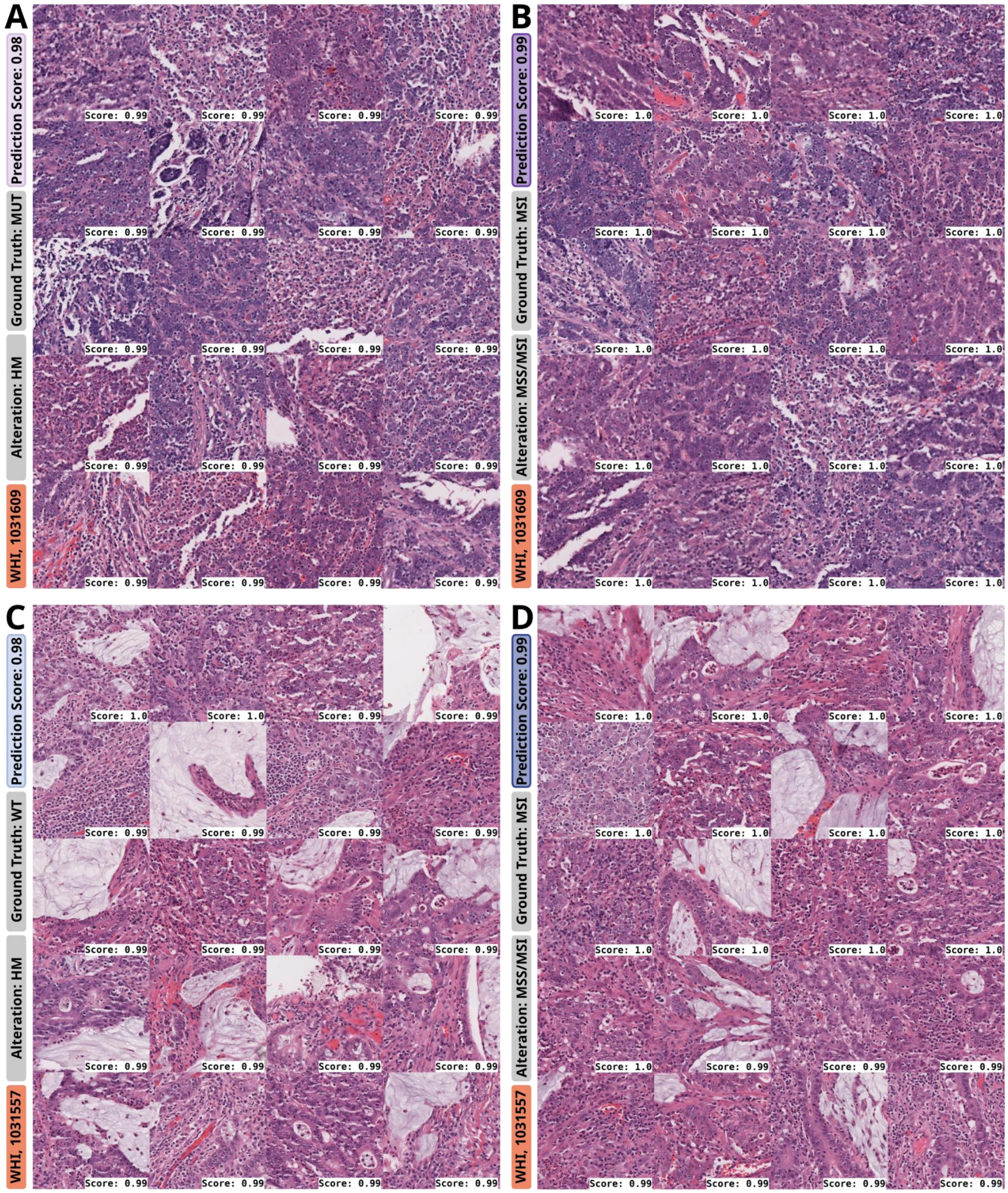

Fig. S12: **Top tiles for prediction of Hypermutation and MSI for two selected slides from Fig. S6.** Each row includes top tiles for one slide (heatmaps in Fig. S6), with tiles for Hypermutation (HM) in the left and MSS/MSI in the right column. A detailed pathological assessment is given in Tab. S19. Abbreviations: HM: Hypermutation; MSI: Microsatellite instability; MSS: Microsatellite stability; MUT: Mutated; WT: Wild type.

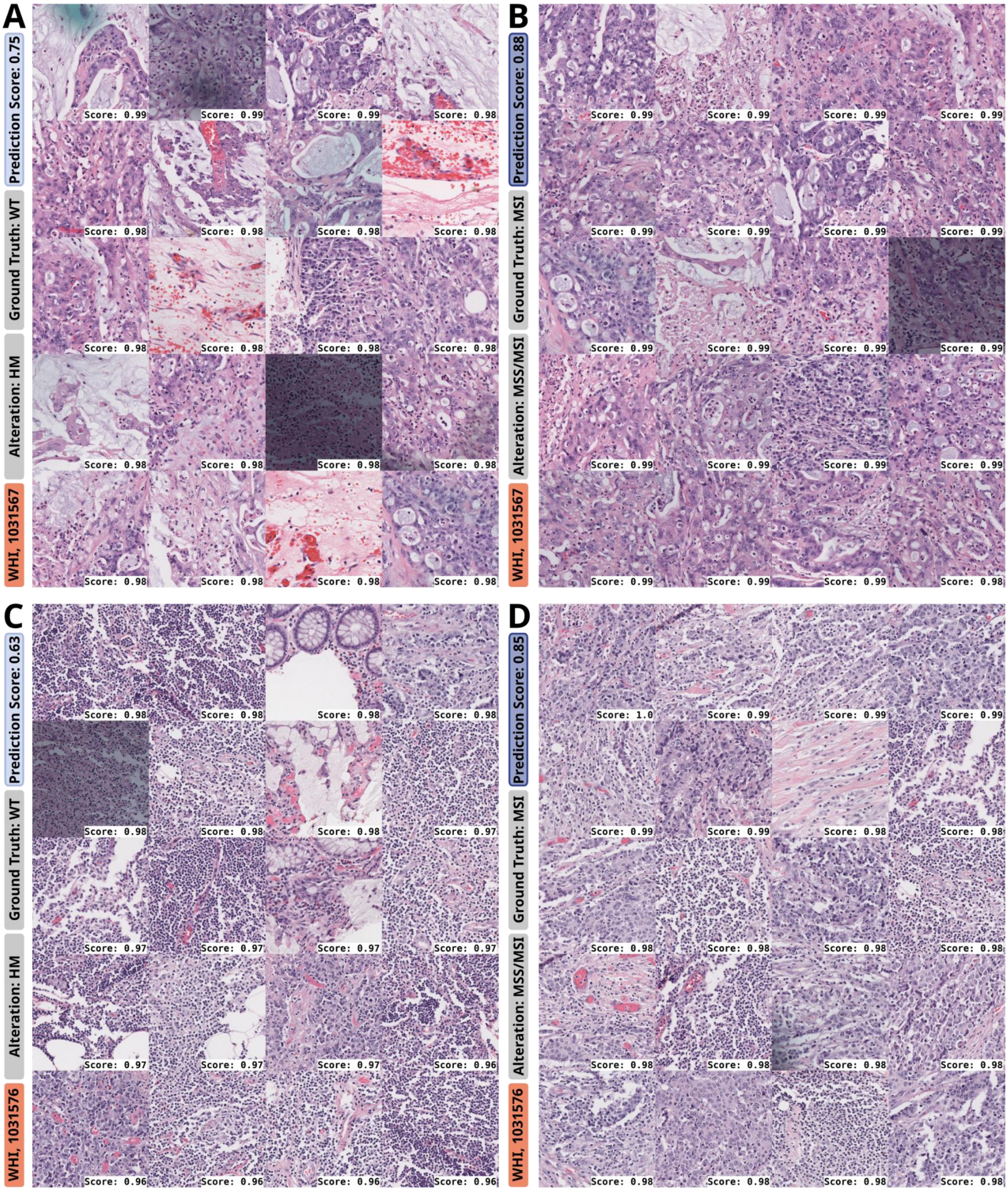

Fig. S13: **Top tiles for prediction of Hypermutation and MSI for two selected slides from Fig. S6.** Each row includes top tiles for one slide (heatmaps in Fig. S6), with tiles for Hypermutation (HM) in the left and MSS/MSI in the right column. A detailed pathological assessment is given in Tab. S19. Abbreviations: HM: Hypermutation; MSI: Microsatellite instability; MSS: Microsatellite stability; MUT: Mutated; WT: Wild type.

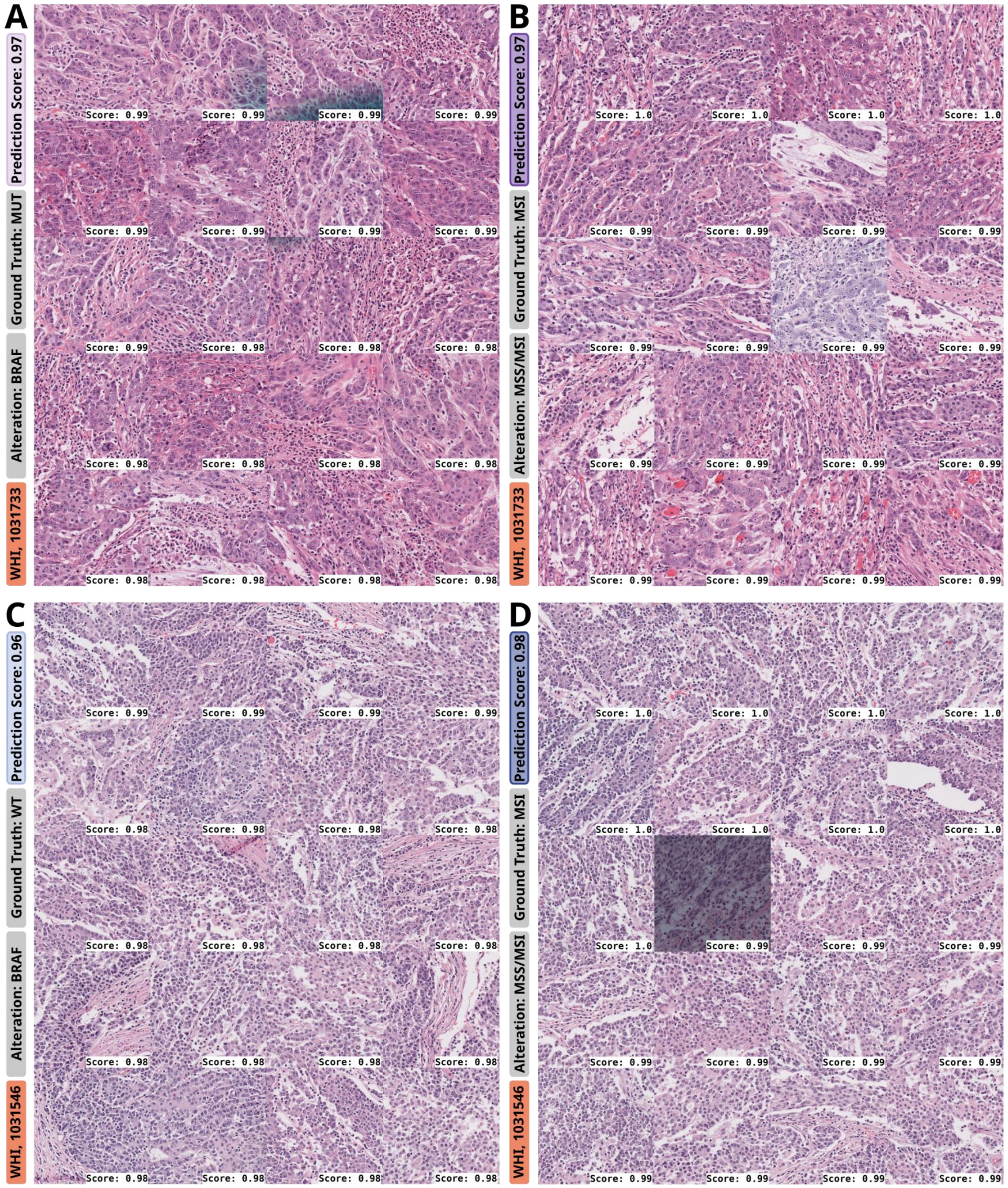

Fig. S14: **Top tiles for prediction of *BRAF* and MSI for two selected slides from Fig. S7.** Each row includes top tiles for one slide (heatmaps in Fig. S7), with tiles for *BRAF* in the left and MSS/MSI in the right column. A detailed pathological assessment is given in Tab. S19. Abbreviations: MSI: Microsatellite instability; MSS: Microsatellite stability; MUT: Mutated; WT: Wild type.

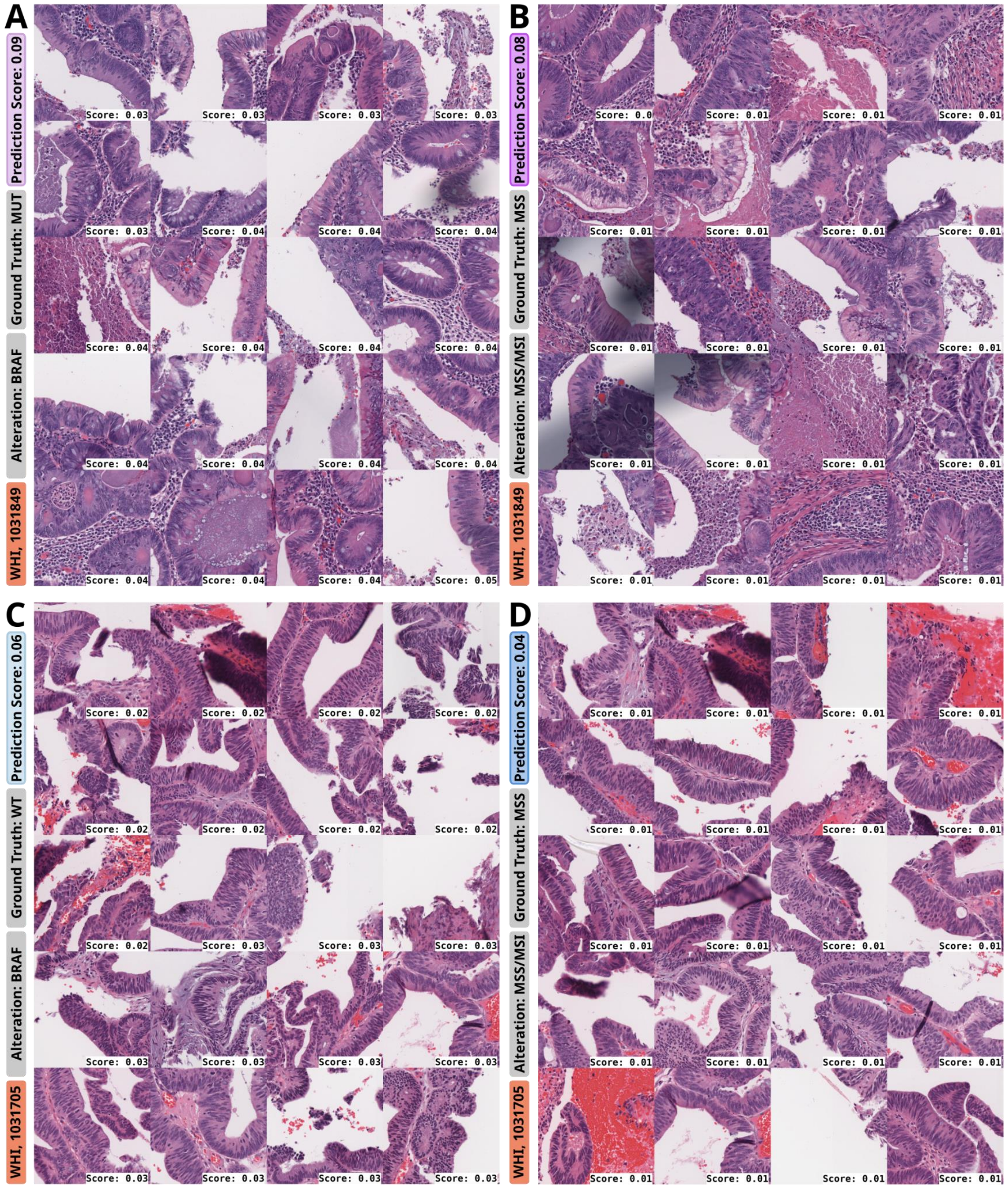

Fig. S15: **Top tiles for prediction of *BRAF* and MSI for two selected slides from Fig. S7.** Each row includes top tiles for one slide (heatmaps in Fig. S7), with tiles for *BRAF* in the left and MSS/MSI in the right column. A detailed pathological assessment is given in Tab. S19. Abbreviations: MSI: Microsatellite instability; MSS: Microsatellite stability; MUT: Mutated; WT: Wild type.

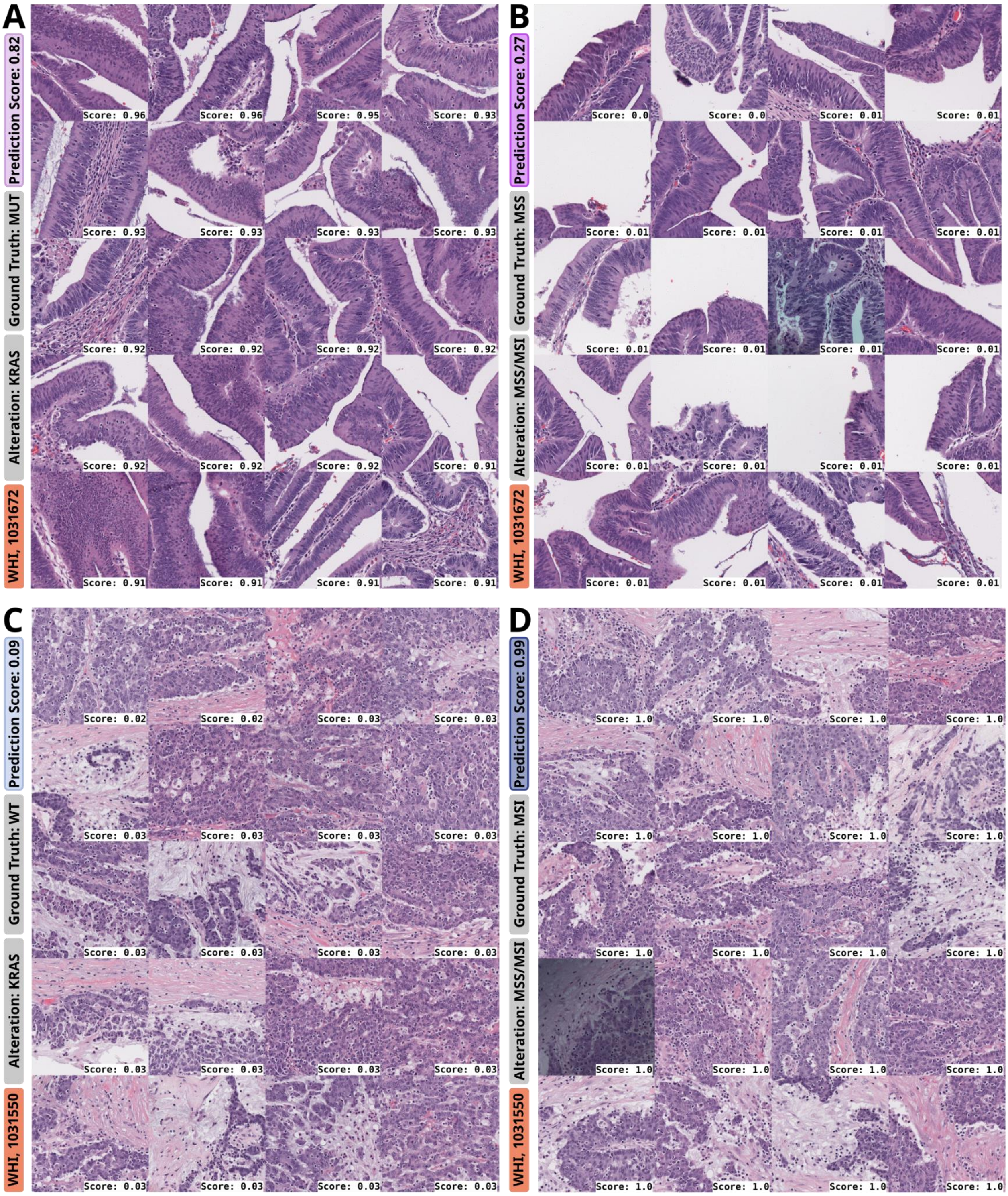

Fig. S16: **Top tiles for prediction of *KRAS* and MSI for two selected slides from Fig. S8.** Each row includes top tiles for one slide (heatmaps in Fig. S8), with tiles for *KRAS* in the left and MSS/MSI in the right column. A detailed pathological assessment is given in Tab. S19. Abbreviations: MSI: Microsatellite instability; MSS: Microsatellite stability; MUT: Mutated; WT: Wild type.

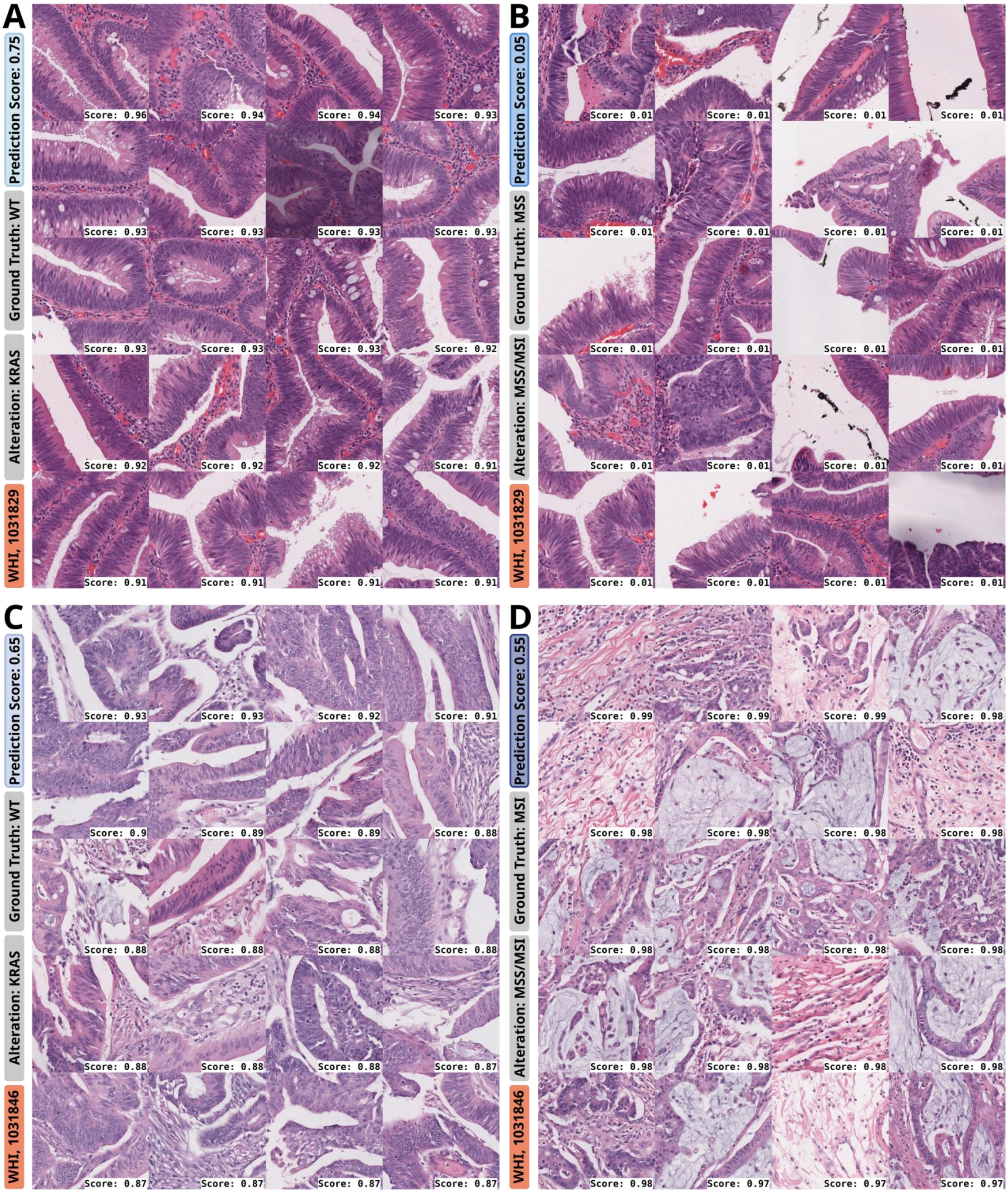

Fig. S17: **Top tiles for prediction of *KRAS* and MSI for two selected slides from Fig. S8.** Each row includes top tiles for one slide (heatmaps in Fig. S8), with tiles for *KRAS* in the left and MSS/MSI in the right column. A detailed pathological assessment is given in Tab. S19. Abbreviations: MSI: Microsatellite instability; MSS: Microsatellite stability; MUT: Mutated; WT: Wild type.

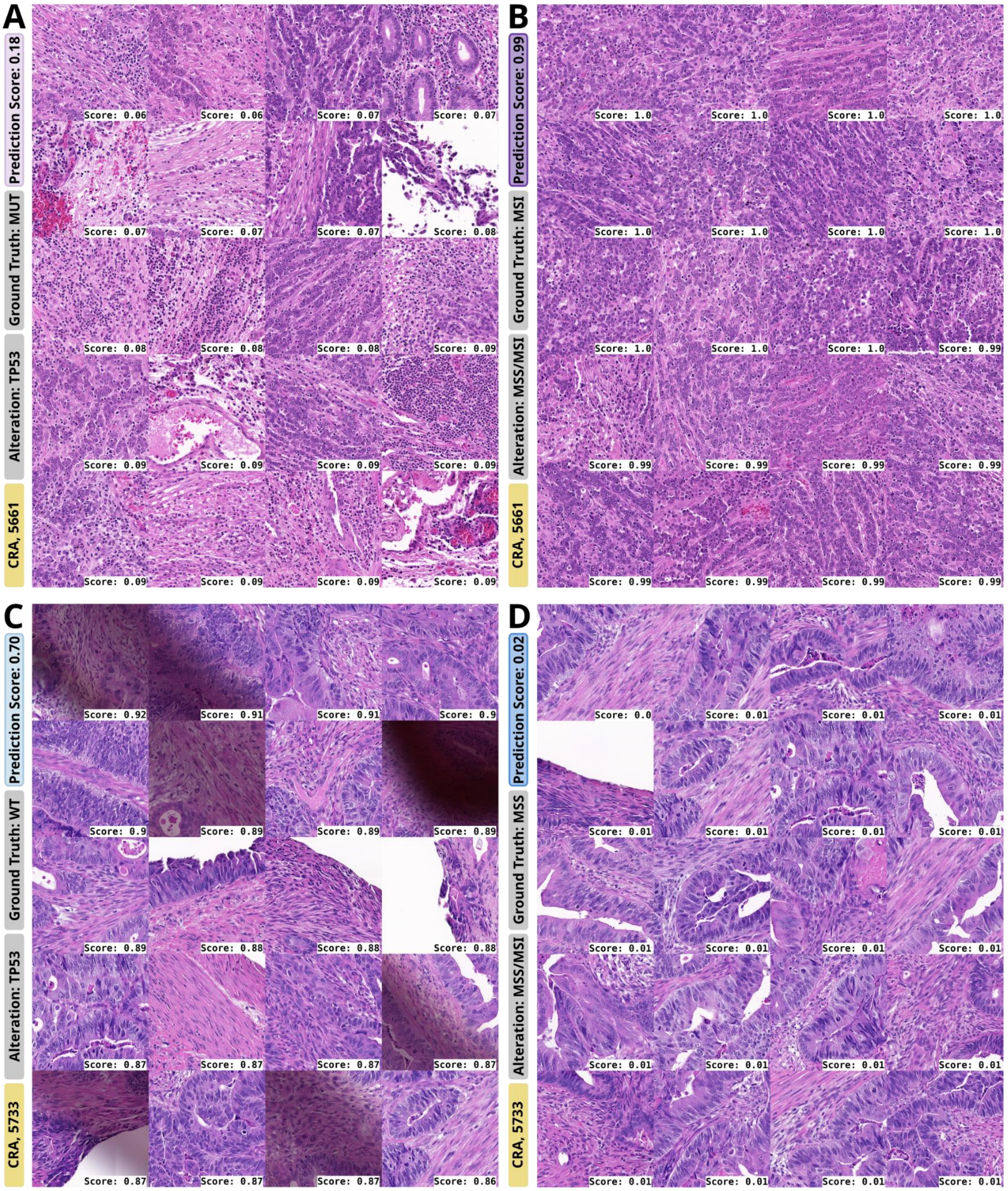

Fig. S18: **Top tiles for prediction of *TP53* and MSI for two selected slides from Fig. S9.** Each row includes top tiles for one slide (heatmaps in Fig. S9), with tiles for *TP53* in the left and MSS/MSI in the right column. A detailed pathological assessment is given in Tab. S19. Abbreviations: MSI: Microsatellite instability; MSS: Microsatellite stability; MUT: Mutated; WT: Wild type.

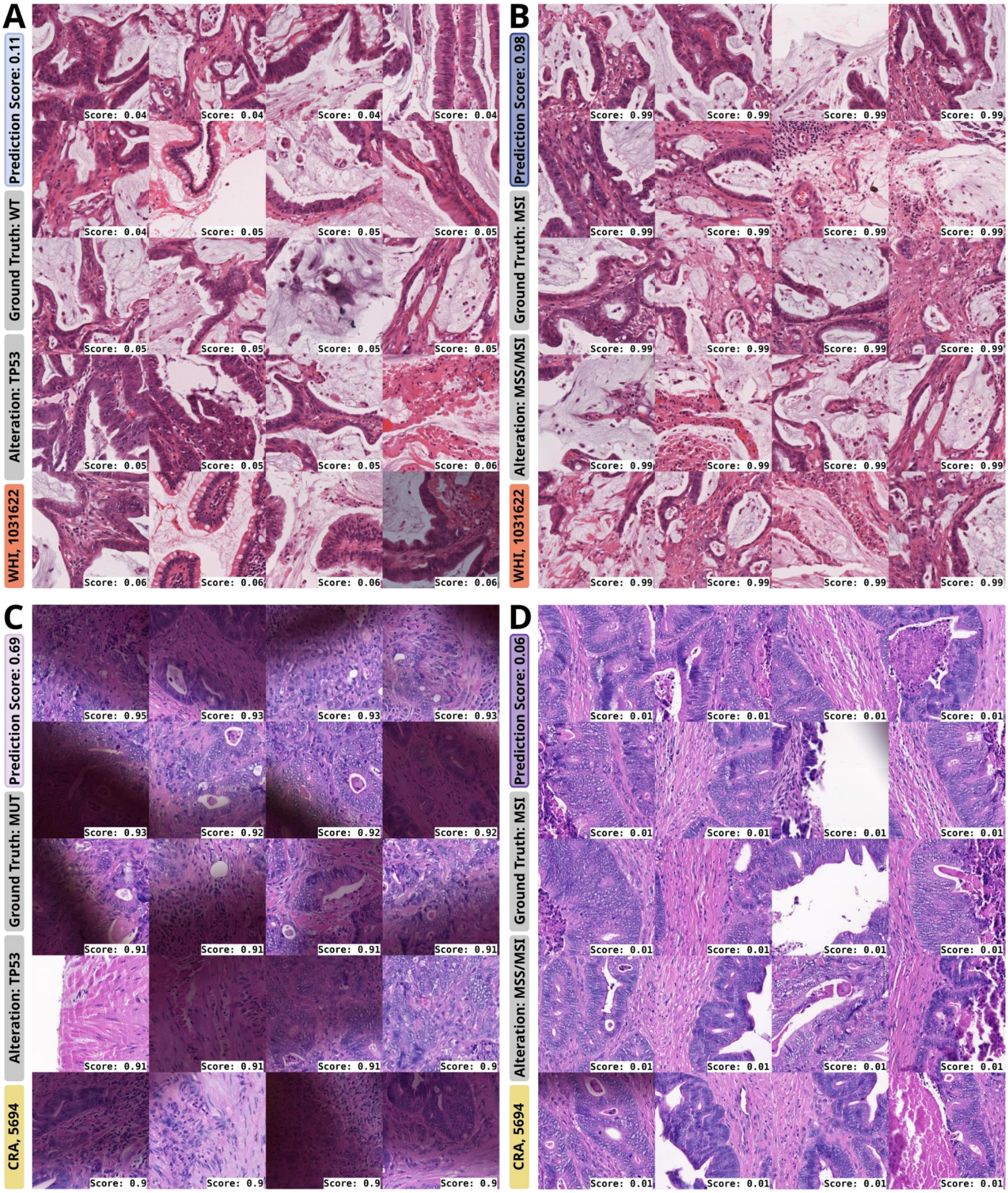

Fig. S19: **Top tiles for prediction of *TP53* and MSI for two selected slides from Fig. S9.** Each row includes top tiles for one slide (heatmaps in Fig. S9), with tiles for *TP53* in the left and MSS/MSI in the right column. A detailed pathological assessment is given in Tab. S19. Abbreviations: MSI: Microsatellite instability; MSS: Microsatellite stability; MUT: Mutated; WT: Wild type.

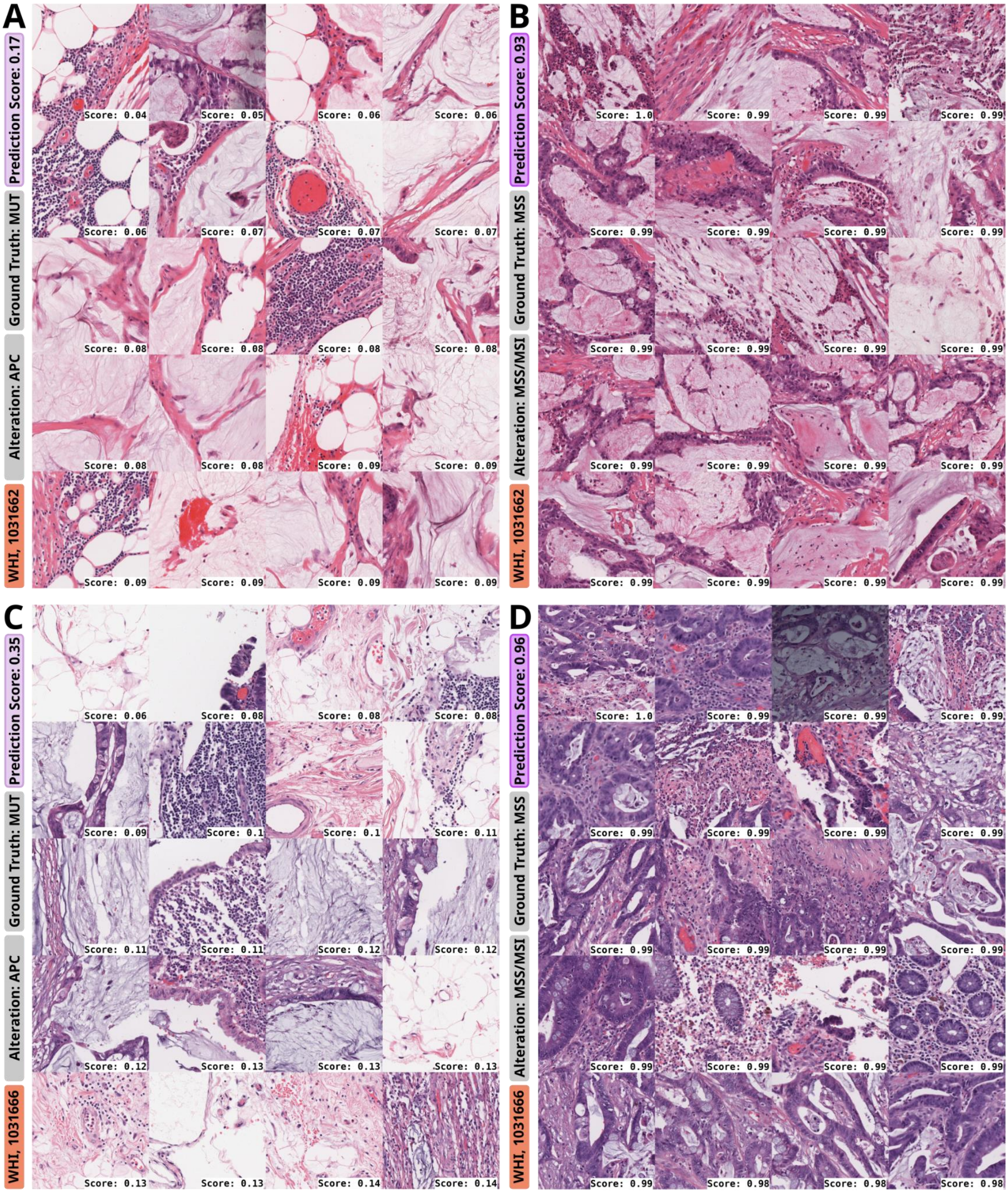

Fig. S20: **Top tiles for prediction of *APC* and MSI for two selected slides from Fig. S10.** Each row includes top tiles for one slide (heatmaps in Fig. S10), with tiles for *APC* in the left and MSS/MSI in the right column. A detailed pathological assessment is given in Tab. S19. Abbreviations: MSI: Microsatellite instability; MSS: Microsatellite stability; MUT: Mutated; WT: Wild type.

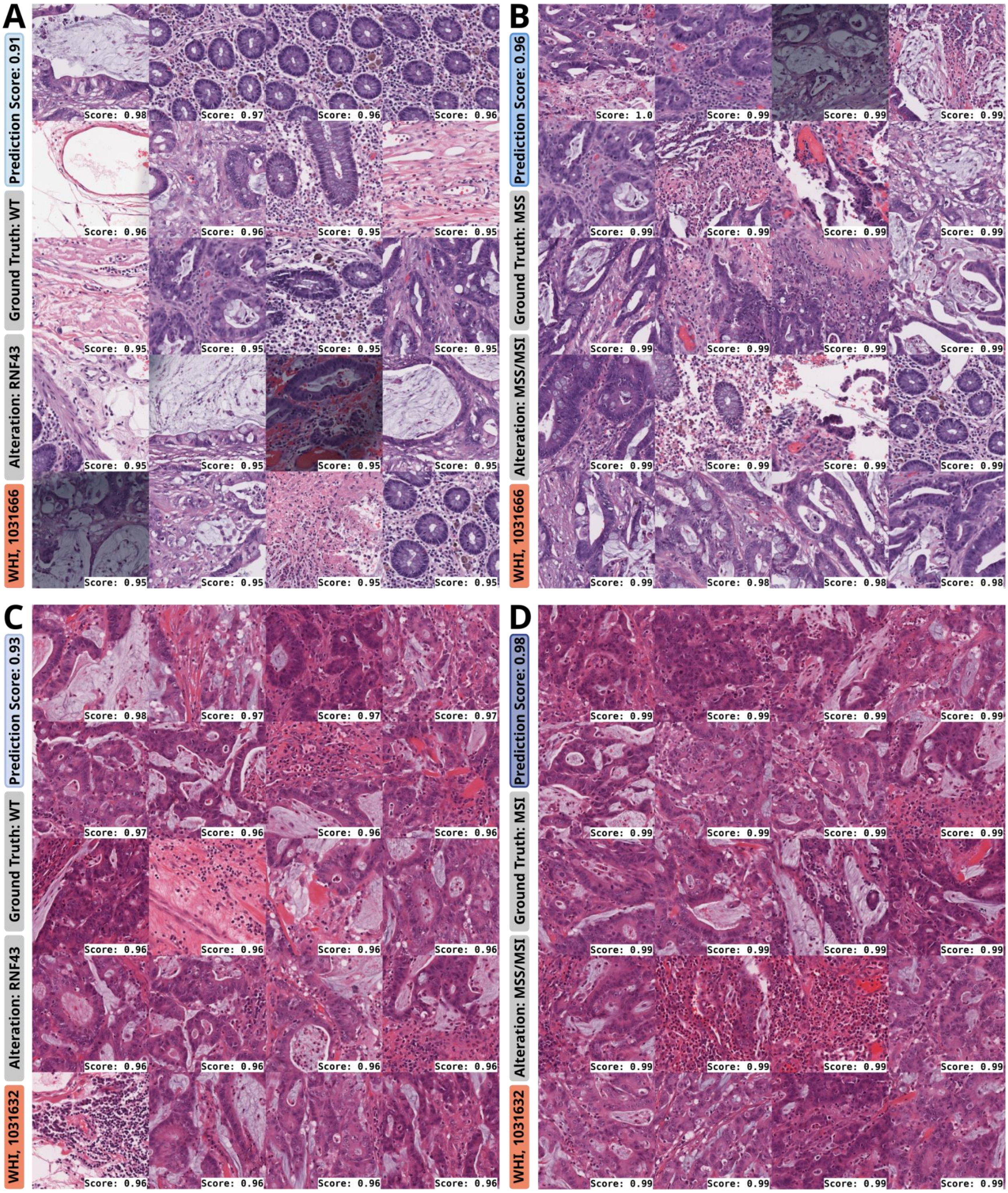

Fig. S21: **Top tiles for prediction of *RNF43* and MSI for two selected slides from Fig. S10.** Each row includes top tiles for one slide (heatmaps in Fig. S10), with tiles for *RNF43* in the left and MSS/MSI in the right column. A detailed pathological assessment is given in Tab. S19. Abbreviations: MSI: Microsatellite instability; MSS: Microsatellite stability; MUT: Mutated; WT: Wild type.

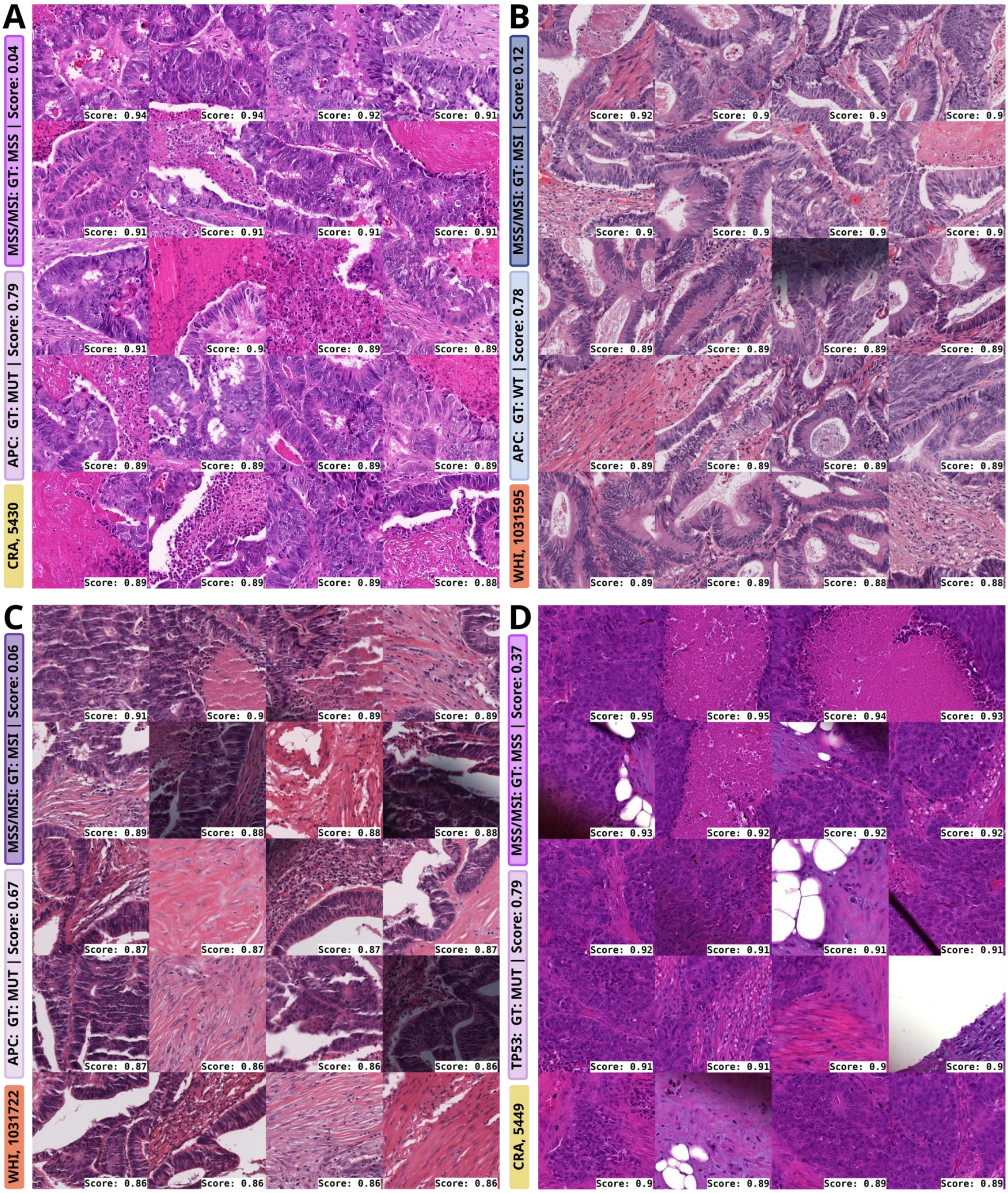

Fig. S22: **Top tiles for prediction of *APC* and *TP53* for selected slides showing tendencies of target specific morphology.** Prediction scores for *APC* and *TP53* seem to be driven by usual gland-forming adenocarcinoma (NOS, ‘not otherwise specified’) with cribriform architecture which can be broadly summarized as ‘MSS-like’ morphology. However, especially in **A** and **D**, extensive necrosis is highlighted. In prior studies, abnormal p53 expression as surrogate for *TP53* mutations was not associated with the extent of tumor necrosis in CRC [9] but in other entities such as lung cancer [10] Hence, for example, extensive necrosis could be considered a potentially interesting finding that requires further investigation in the context of the genetic alteration specificity (*TP53* and/or *APC*). However, the morphologic findings here are not distinct. The score for MSS/MSI and the genetic alteration is the prediction score assigned to the slide by the model. The individual tile prediction scores are given with the individual tiles. Abbreviations: GT: Ground Truth; MSI:

Microsatellite instability; MSS: Microsatellite stability; MUT: Mutated; NOS: Not otherwise specified; WT: Wild type.

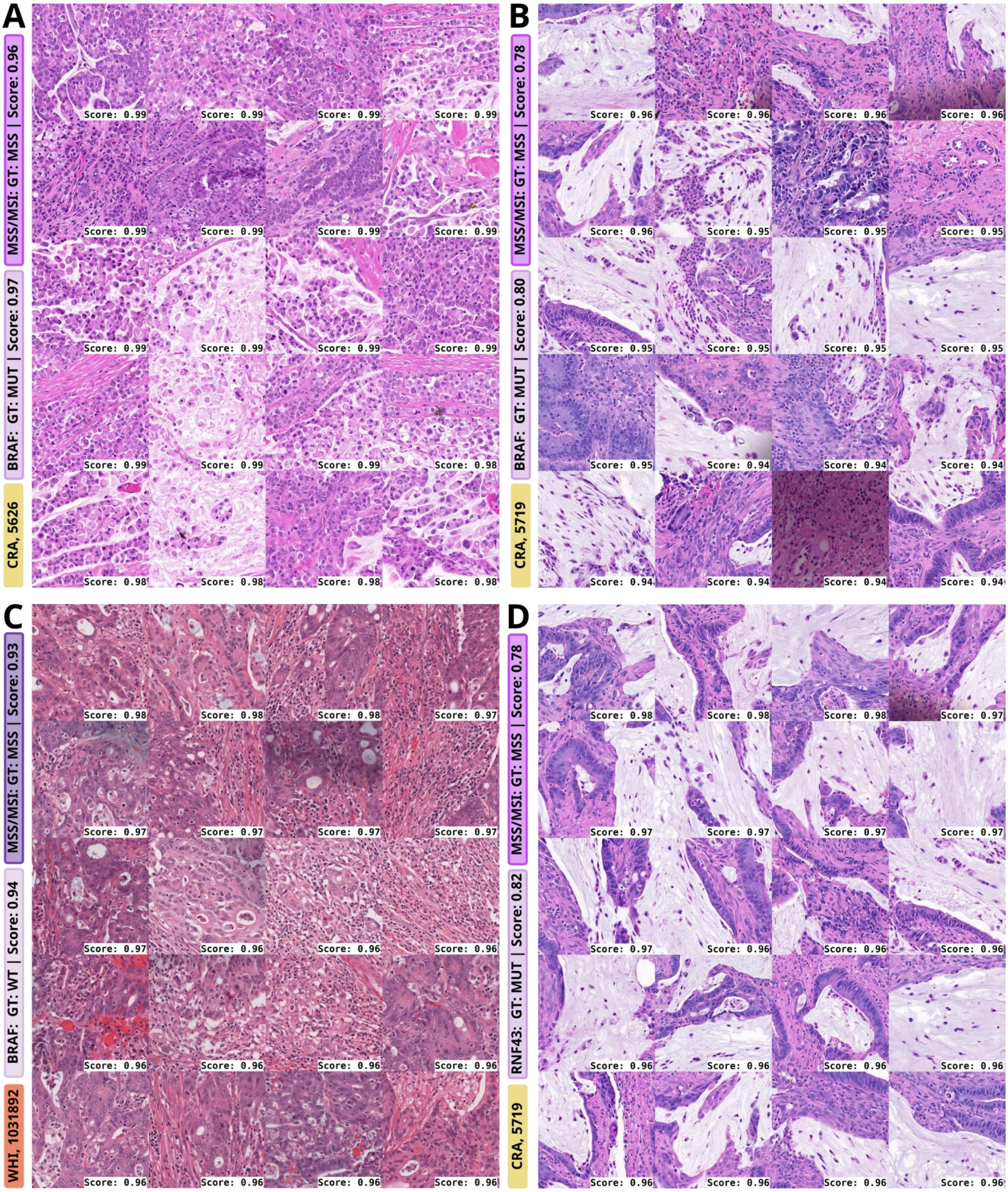

Fig. S23: **Top tiles for prediction of *BRAF* and *RNF43* for selected slides showing tendencies of target specific morphology.** Mucinous morphology and signet-ring cell differentiation displayed in **A** and **B** may be part of a *BRAF* specific morphology [11,12], potentially even beyond MSI, which is also associated with our findings. Moreover, poorly differentiated clusters and tumor budding are highlighted for *BRAF* prediction (**C**). Tumor budding was shown to be associated with *BRAF* mutations [13], and interestingly is inversely correlated with MSI [14] The prediction of *RNF43* MUT seems to be associated with mucinous histology, which is also a typical MSI-like morphology but also further associated with *RNF43* [15] Whether these morphologic findings are alteration specific beyond MSI requires further investigation. The score for MSS/MSI and the genetic alteration is the prediction score assigned to the slide by the model. The individual tile prediction scores are given with the individual tiles. Abbreviations: GT: Ground Truth; MSI: Microsatellite

instability; MSS: Microsatellite stability; MUT: Mutated; WT: Wild type.

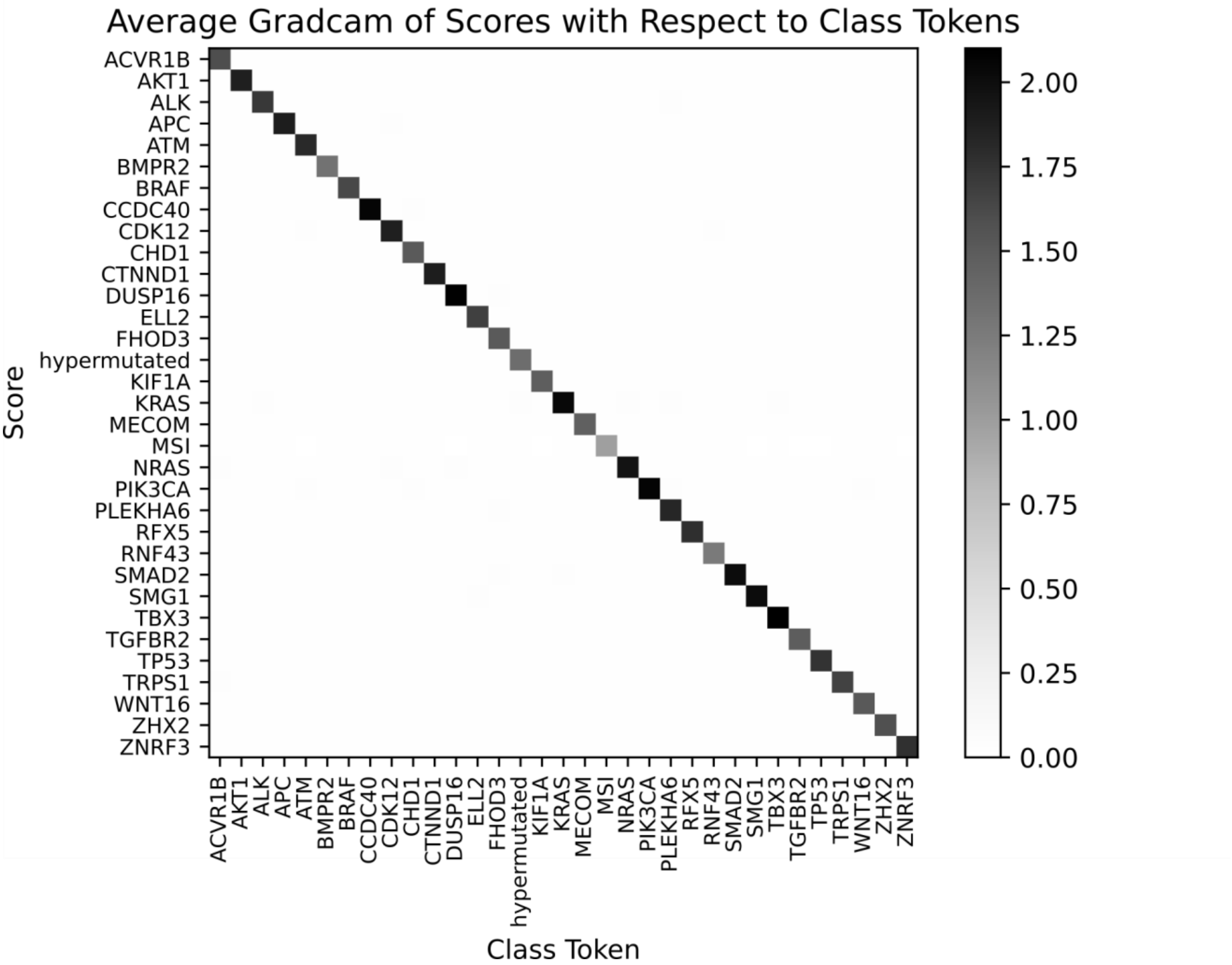

Fig. S24: **Average Grad-CAM of scores with respect to class tokens**: A heatmap illustrating the cross-correlation of Grad-CAM values between scores and class tokens across multiple genetic targets. The results demonstrate that the prediction of each target predominantly relies on its corresponding token in the decoder, indicating a clear token-specific attribution.

References

1. Murtagh F, Legendre P. Ward's Hierarchical Clustering Method: Clustering Criterion and Agglomerative Algorithm [Internet]. arXiv [stat.ML]. 2011. Available from: http://arxiv.org/abs/1111.6285

2. Scitovski R, Sabo K, Martínez-Álvarez F, Ungar Š. Cluster Analysis and Applications. Springer International Publishing; 2021. 10 p.

3. Murtagh F, Legendre P. Ward's hierarchical agglomerative clustering method: Which algorithms implement ward's criterion? J Classif. 2014 Oct 18;31(3):274–95.

4. Han J, Kamber M, Pei J. Data Mining: Concepts and Techniques. 3rd ed. Oxford, England: Morgan Kaufmann; 2011. 744 p. (The Morgan Kaufmann Series in Data Management Systems).

5. Hahsler M, Grün B, Hornik K. Arules- A computational environment for mining association rules and frequent item sets. J Stat Softw. 2005 Sep 29;14(15):1–25.

6. Piatetsky-Shapiro G. Discovery, Analysis, and Presentation of Strong Rules. Knowledge Discovery in Data-bases. 1991;229–48.

7. Brin S, Motwani R, Ullman JD, Tsur S. Dynamic itemset counting and implication rules for market basket data. SIGMOD Rec. 1997 Jun 1;26(2):255–64.

8. Yan X, Zhang C, Zhang S. CONFIDENCE METRICS FOR ASSOCIATION RULE MINING. Appl Artif Intell. 2009 Oct 16;23(8):713–37.

9. Pollheimer MJ, Kornprat P, Lindtner RA, Harbaum L, Schlemmer A, Rehak P, et al. Tumor necrosis is a new promising prognostic factor in colorectal cancer. Hum Pathol. 2010 Dec;41(12):1749–57.

10. Swinson DEB, Jones JL, Richardson D, Cox G, Edwards JG, O'Byrne KJ. Tumour necrosis is an independent prognostic marker in non-small cell lung cancer: correlation with biological variables. Lung Cancer. 2002 Sep;37(3):235–40.

11. Pai RK, Jayachandran P, Koong AC, Chang DT, Kwok S, Ma L, et al. BRAF-mutated, microsatellite-stable adenocarcinoma of the proximal colon: an aggressive adenocarcinoma with poor survival, mucinous differentiation, and adverse morphologic features. Am J Surg Pathol. 2012 May;36(5):744–52.

12. Yalcin S, Onguru O. BRAF mutation in colorectal carcinomas with signet ring cell component. Cancer Biol Med. 2017 Aug;14(3):287–92.

13. Trinh A, Lädrach C, Dawson HE, Ten Hoorn S, Kuppen PJK, Reimers MS, et al. Tumour budding is associated with the mesenchymal colon cancer subtype and RAS/RAF mutations: a study of 1320 colorectal cancers with Consensus Molecular Subgroup (CMS) data. Br J Cancer. 2018 Nov;119(10):1244–51.

14. Hatthakarnkul P, Quinn JA, Matly AAM, Ammar A, van Wyk HC, McMillan DC, et al. Systematic review of tumour budding and association with common mutations in patients with colorectal cancer. Crit Rev Oncol Hematol. 2021 Nov;167:103490.

15. Bugter JM, El Bouazzaoui L, Küçükköse E, Hong Y, Sprangers J, Jordens I, et al. RNF43 mutations facilitate mucinous colorectal cancer metastasis via formation of a tumour-intrinsic niche [Internet]. bioRxiv. 2022 [cited 2024 Feb 28]. p. 2022.12.22.521159. Available from: https://www.biorxiv.org/content/10.1101/2022.12.22.521159v1
